# Mitochondrial genome transfer drives metabolic reprogramming in adjacent colonic epithelial cells promoting TGFβ1-mediated tumor progression

**DOI:** 10.1038/s41467-024-48100-y

**Published:** 2024-04-30

**Authors:** Bingjie Guan, Youdong Liu, Bowen Xie, Senlin Zhao, Abudushalamu Yalikun, Weiwei Chen, Menghua Zhou, Qi Gu, Dongwang Yan

**Affiliations:** 1 Department of General Surgery, Shanghai General Hospital, Shanghai Jiao Tong University School of Medicine, Shanghai, China; 2 Department of Colorectal Surgery, Fudan University Shanghai Cancer Center, Shanghai, China; 3 Department of Oncology, Shanghai Medical College, Fudan University, Shanghai, China

**Keywords:** Colon cancer, Cancer microenvironment, Cancer metabolism

## Abstract

Although nontumor components play an essential role in colon cancer (CC) progression, the intercellular communication between CC cells and adjacent colonic epithelial cells (CECs) remains poorly understood. Here, we show that intact mitochondrial genome (mitochondrial DNA, mtDNA) is enriched in serum extracellular vesicles (EVs) from CC patients and positively correlated with tumor stage. Intriguingly, circular mtDNA transferred via tumor cell-derived EVs (EV-mtDNA) enhances mitochondrial respiration and reactive oxygen species (ROS) production in CECs. Moreover, the EV-mtDNA increases TGFβ1 expression in CECs, which in turn promotes tumor progression. Mechanistically, the intercellular mtDNA transfer activates the mitochondrial respiratory chain to induce the ROS-driven RelA nuclear translocation in CECs, thereby transcriptionally regulating TGFβ1 expression and promoting tumor progression via the TGFβ/Smad pathway. Hence, this study highlights EV-mtDNA as a major driver of paracrine metabolic crosstalk between CC cells and adjacent CECs, possibly identifying it as a potential biomarker and therapeutic target for CC.

## Abstract (web summary)

The interaction between colon cancer cells and colonic epithelial cells (CECs) is critical yet not well-known. Here, the authors show that tumor extracellular vesicles mediate mitochondrial DNA transfer to CECs, initiating mitochondrial activation and RelA-induced TGFβ1 expression, leading to tumor progression.

## Introduction

Colon cancer (CC) remains the second leading cause of cancer-related death worldwide^1^. The recent therapeutic progress made in microsatellite instability-high CC using immune checkpoint inhibitors shows the great importance of investigating the tumor microenvironment (TME)^2^. However, the prognosis of most patients with CC, especially those with advanced-stage disease, is relatively poor^3^. The discovery of novel molecular targets and underlying mechanisms of CC progression is therefore a pressing need.

Tumor progression is a complicated pathological process requiring adequate communication with other kinds of cells in the TME^4^. Increasing evidence suggests that nontumor cells educated by cancer cell-derived extracellular vesicles (EVs) play an essential role in tumor development^5^. As critical vehicles of intercellular communication, EVs derived from CC cells transfer numerous bioactive molecules, including proteins, nucleic acids, and metabolites, into recipient cells, reshaping immune networks, host-microbe homeostasis, and metabolic pathways in the TME to confer a survival advantage on cancer cells^5,6^.

Notably, during CC development, the major cell type surrounding the primary tumor is the normal colonic epithelial cell (CEC)^7^. Accumulating studies have highlighted crosstalk between cancer cells and neighboring normal epithelial cells, a phenomenon that is responsible for malignant progression. For example, healthy liver cells regulate the viability of liver cancer cells through the Hippo pathway^8^. Lung metastatic cancer cells drive adjacent alveolar epithelial cells to acquire stem cell-like features, which in turn promote the proliferation of the cancer cells^9^. However, the interactions between CC cells and adjacent normal CECs are poorly understood. A few investigations have suggested some unique phenotypes of normal tissues adjacent to the tumor (NAT) relative to tumor tissues and normal tissues not located near the tumor^10,11^. Unlike CC cells, NAT display markedly enhanced oxidative phosphorylation (OXPHOS) compared with healthy colonic mucosa^11^. However, the molecular mechanism underlying this phenomenon remains enigmatic.

The human mitochondrial genome is composed of a 16.6 kb circular double-stranded DNA molecule (mitochondrial DNA, mtDNA) that encodes key catalytic subunits essential for OXPHOS^12^. Of note, the mtDNA copy number is increased in most CC cells^13^. More interestingly, there is a positive correlation between circulating mtDNA levels and CC risk, indicating a potential role of mtDNA in the TME of CC^14^.

In this study, we report that intercellular transfer of complete circular mtDNA via cancer cell-derived EVs results in enhanced OXPHOS in adjacent normal CECs. Subsequently, the increase in reactive oxygen species (ROS) generated by OXPHOS activates the NF-κB signaling pathway, which drives *TGFβ1* transcription and protein secretion in normal CECs and turn promotes tumor progression.

## Results

### Complete circular mtDNA correlated with tumor malignancy is enriched in CC cell-derived EVs

**Fig. 1:**
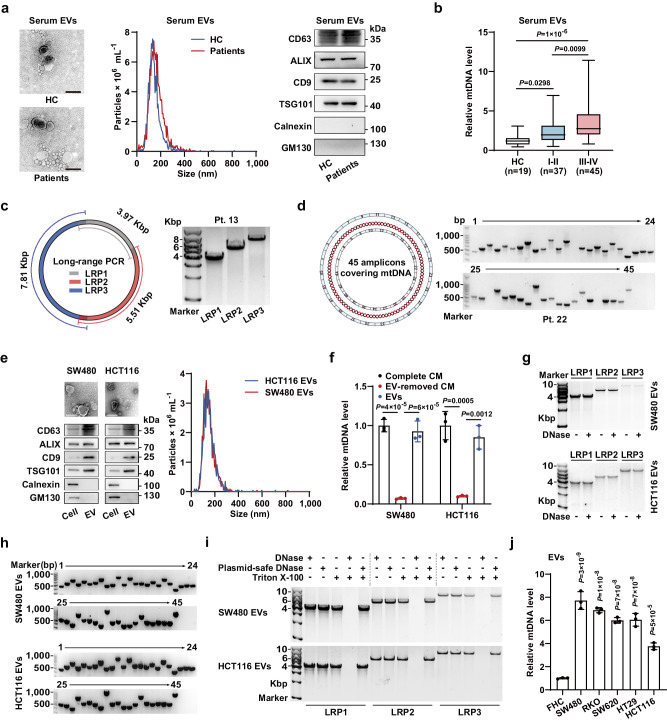
Complete circular mtDNA is enriched in CC cell-derived EVs. **a** Identification of EVs from the serum of healthy controls (HCs) or CC patients. The samples derive from the same experiment but different gels for CD63, GM130, and ALIX, another for Calnexin, TSG101, and another for CD9 were processed in parallel. Scale bar, 200 nm. **b** The relative mtDNA levels in serum EVs from HCs and patients with CC were measured by qPCR targeting the mitochondrial *ND1* (2 ng of DNA was used). **c** Schematic diagram (left) and representative agarose gel image (right) of long-range PCR with three amplicons covering the entire mtDNA obtained from serum EVs of patients (1 ng of DNA per reaction). **d** Schematic diagram (left) and representative gel image (right) of 45 overlapping amplicons encoding the whole mtDNA from serum EVs of patients (1 ng of DNA per PCR). **e** Identification of EVs from CC cells. The samples derive from the same experiment but different gels for CD63, GM130, and ALIX, another for Calnexin, TSG101, and another for CD9 were processed in parallel. Scale bar, 200 nm. **f** The mtDNA levels in SW480 and HCT116 cell-derived complete CM, EV-removed CM, and the corresponding isolated EVs were measured. *n* = 3 independent experiments. **g** Representative agarose gel image of long-range PCR using three amplicons covering the entire mtDNA from SW480 and HCT116 cell-derived EVs. **h** Representative gel image of 45 overlapping amplicons encoding the whole mtDNA from SW480 and HCT116 cell-derived EVs pretreated with DNase. **i** Representative agarose gel image of long-range PCR with three amplicons covering the whole mtDNA from SW480 and HCT116 cell-derived EVs pretreated as indicated. **j** The relative mtDNA levels in EVs derived from the human CC cells and FHC normal human CECs were measured. *n* = 3 independent experiments. Data are means ± SD. The boxplots indicate median (center), 25th and 75th percentiles (bounds of box), and 2.5th and 97.5th percentiles (whiskers). One-way ANOVA with Tukey’s multiple comparisons test (**f**, **j**). Kruskal–Wallis test with two-sided Dunn’s multiple comparisons test (**b**). Source data are provided as a Source Data file.

Considering the finding that a high level of circulating mtDNA is closely related to CC, we hypothesized that mtDNA secreted by CC cells might be packaged into circulating EVs. We isolated EVs from the serum of CC patients and healthy donors and then identified them by electron microscopy, nanoparticle tracking analysis, and immunoblotting for protein markers (Fig. 1a). Subsequently, qPCR analysis revealed higher levels of serum EV-carried mtDNA (EV-mtDNA) in CC patients, which was correlated with advanced tumor stage (Fig. 1b, Supplementary Fig. 1a and Supplementary Table 1).

Since DNA in EVs is often present as broken fragments^15^, we sought to determine whether the mtDNA genome carried in circulating EVs is complete. Long-range PCR was then performed with three reported amplicons covering the entire mtDNA genome^16^, showing that serum EV-mtDNA was the complete 16.6 kb circular dsDNA molecule (Fig. 1c and Supplementary Fig. 1b). This result was validated by PCR amplification of 45 overlapping amplicons encompassing the whole mtDNA genome (Fig. 1d and Supplementary Fig. 1c).

Moreover, we extracted EVs from conditioned media (CM) of five CC cell lines (SW480, HCT116, RKO, SW620, and HT29). Identification of these EVs was performed (Fig. 1e and Supplementary Fig. 1d–f). Notably, the mtDNA level in the EVs was nearly equal to that in complete CM, suggesting that EVs are major carriers of extracellular mtDNA (Fig. 1f and Supplementary Fig. 1g). The majority of EV-carried DNA is located on the outer surface of EVs^16^, however, the long-range PCR revealed the mtDNA content was similar between EVs with and without DNase treatment, indicating that EV-mtDNA is localized mainly inside EVs (Fig. 1g and Supplementary Fig. 1h). Additionally, Amplification of 45 overlapping amplicons demonstrated the entire mitochondrial genome within CC cell-derived EVs (Fig. 1h and Supplementary Fig. 1i).

We further characterized the isolated EVs by sucrose density ultracentrifugation. Of note, EV-mtDNA and protein markers were mainly detected within the small EV (sEV)-containing density range (F2-3, 1.13–1.19 g mL^−1^) instead of the large EV (lEV)-containing range^17^, and inhibition of sEV secretion by GW4869 sharply decreased the mtDNA content in the tumor CM (Supplementary Fig. 2a–f). These findings, together with the above results that the majority of the collected EVs were under 200 nm in size, indicate that mtDNA is primarily enriched in sEVs (exosomes often predominate) secreted by CC cells. We then employed plasmid-safe DNase, which selectively hydrolyzes linear dsDNA and not closed circular dsDNA. Notably, treatment with Triton X-100 and plasmid-safe DNase did not decrease the EV-mtDNA content, strongly supporting the finding that CC cell-derived EV-mtDNA was present as a complete circular dsDNA molecule and not as broken DNA fragments (Fig. 1i). The degradative activity of plasmid-safe DNase toward linear dsDNA was verified (Supplementary Fig. 2g). In addition, the EVs had lower concentrations of Histone 3 and calreticulin than parental cell lysates, implying that there was minimal to no cellular debris contamination of the EVs collected via this method (Supplementary Fig. 2h–l). Since lEVs may also carry mitochondrial components, we further compared the levels of mitochondrial constituents in lEVs and sEVs secreted by CC cells^18^. We verified that mtDNA was predominantly enriched in sEVs rather than lEVs and that both vesicle types contained negligible amounts of mitochondrial proteins, indicating that the selective enrichment of mtDNA in sEVs was not a random occurrence (Supplementary Fig. 2m–o). Notably, qPCR analysis showed that the EV-mtDNA level was significantly higher in EVs derived from CC cells than in EVs derived from the human normal CEC line FHC (Fig. 1j). Taken together, the above results demonstrate that complete circular mtDNA is enriched in CC cell-secreted sEVs and positively correlated with tumor stage.

### CECs display enhanced mitochondrial respiration upon EV-mediated communication with CC cells

**Fig. 2:**
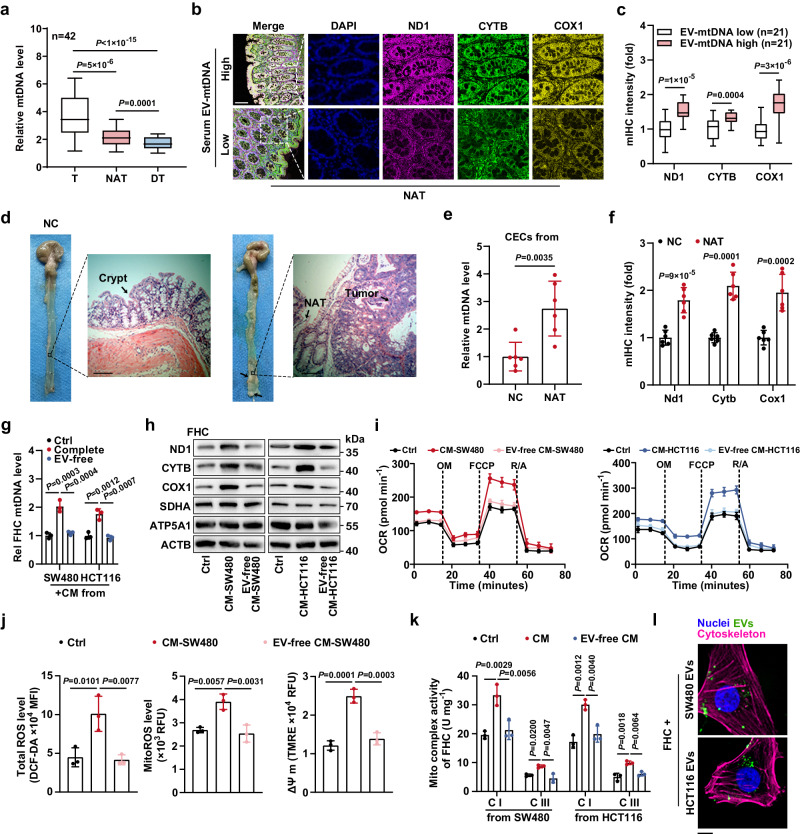
CECs exhibit enhanced OXPHOS upon communication with CC cells in an EV-dependent manner. **a** The mtDNA levels in tumor tissues (T), normal tissues adjacent to the tumor (NAT), and paired distant colonic tissues (DT) from 42 CC patients were measured. The boxplots indicate median (center), 25th and 75th percentiles (bounds of box), and 2.5th and 97.5th percentiles (whiskers). **b** Representative images of mIHC staining for the indicated proteins in sections from NAT of patients. Scale bar, 100 μm. **c** The coexpression of mitochondrial proteins was evaluated. The boxplots indicate median (center), 25th and 75th percentiles (bounds of box), and 2.5th and 97.5th percentiles (whiskers). **d** Representative gross view and HE images of normal colonic crypts and NAT from healthy mice (negative control, NC) and murine orthotopic CC model, respectively. Scale bar, 100 μm. **e** The mtDNA levels in CECs from NC mice or NAT were measured. *n* = 6 mice per group. **f** The coexpression of mitochondrial proteins was evaluated. *n* = 6 mice per group. Changes in the (**g**) mtDNA content, (**h**) expression levels of mitochondrial proteins, and (**i**) oxygen consumption rate (OCR) in FHC cells incubated with SW480 or HCT116 cell-derived complete CM or EV-removed CM. The samples derive from the same experiment but different gels for SDHA, COX1, and ND1, another for ACTB, ATP5A1, and another for CYTB were processed in parallel. Rel, relative. *n* = 3 independent experiments. **j** Changes in ROS levels and mitochondrial membrane potential (ΔΨ m) in FHC cells incubated with SW480 cell-derived complete CM or EV-removed CM. *n* = 3 independent experiments. **k** Changes in the activities of mitochondrial complexes I and III in FHC cells incubated with SW480 or HCT116 cell-derived complete CM or EV-free CM. *n* = 3 independent experiments. **l** Example images of CC cell-derived EV uptake by FHC cells. Scale bar, 10 μm. Data are means ± SD. Two-tailed t test (**c**, **e**, and **f**). One-way ANOVA with Tukey’s multiple comparisons test (**g**, **j**, and **k**). Friedman test with two-sided Dunn’s multiple comparisons test (**a**). Source data are provided as a Source Data file.

Next, we showed a marked increase in the mtDNA content in CC tissues relative to that in paired NAT or distant colonic tissues (DT, Fig. 2a). Intriguingly, NAT also exhibited an increase in mtDNA content and ROS levels compared with paired DT in CC patients, strongly suggesting that tumor tissues may influence their surroundings (Fig. 2a and Supplementary Fig. 3a). Then, multiplex immunohistochemistry (mIHC) showed that cytoplasmic coexpression of ND1, CYTB, and COX1, representative mtDNA-encoded respiratory chain subunits, was increased dramatically in NAT from patients with high levels of serum EV-mtDNA (Fig. 2b, c). As completely normal colonic mucosa from living individuals in good health is not clinically available due to constraints of medical ethics, we further compared the mtDNA content in normal colonic mucosa from healthy mice with that in NAT from an azoxymethane/dextran sodium sulfate (AOM/DSS)-induced murine orthotopic CC model, a tool extensively used for intestinal TME studies (Fig. 2d). As expected, the mtDNA copy number and ROS levels were significantly increased in CECs isolated from NAT in tumor-bearing mice relative to those isolated from normal control mice (Fig. 2e and Supplementary Fig. 3b). Coexpression of Nd1, Cytb, and Cox1 was also markedly upregulated in NAT (Fig. 2f and Supplementary Fig. 3c). Moreover, mtDNA was also enriched in murine EVs derived from tumor cells, which were isolated from the murine orthotopic CC model (Supplementary Fig. 3d). These results imply that tumor cells might induce mitochondrial metabolic remodeling in adjacent CECs, perhaps in an EV-dependent manner.

To test this hypothesis, we incubated FHC cells with CM from SW480 or HCT116 CC cells. CCK8 assays indicated that neither CM from SW480 cells (CM-SW480) nor CM from HCT116 cells (CM-HCT116) affected the proliferative potential of FHC cells (Supplementary Fig. 3e, f). Notably, complete CM from tumor cells markedly increased the mtDNA copy number in FHC cells, while removing EVs from the CM impaired the CM-induced increase in the mtDNA content in FHC cells (Fig. 2g). Moreover, complete CM from CC cells but not EV-removed CM induced the expression of mtDNA-encoded ND1, CYTB, and COX1 in FHC cells, although the expression of the nuclear-encoded mitochondrial proteins SDHA and ATP5A1 remained unchanged, which was additionally confirmed by qPCR, suggesting that CC cell-derived EVs mainly regulate the mitochondrial genome (Fig. 2h and Supplementary Fig. 3g). Next, we found that the basal and spare respiratory capacities of FHC cells were higher after stimulation by complete CM but not the CM devoid of EVs (pre-characterized as sEVs) (Fig. 2i and Supplementary Fig. 3h, i). Moreover, the total ROS and mitochondrial ROS (mitoROS) levels in recipient cells were concomitantly elevated in an EV-dependent manner, consistent with the increased mitochondrial membrane potential (ΔΨ m) (Fig. 2j and Supplementary Fig. 3j, k). Furthermore, CC cell-derived EVs markedly enhanced the activities of complexes, such as CI and CIII, in recipient cells (Fig. 2k and Supplementary Fig. 3l). In addition, uptake of tumor cell-derived EVs by FHC cells was directly visualized by confocal microscopy (Fig. 2l). Collectively, CC cell-derived EVs are taken up by CECs, consequently enhancing mitochondrial respiration in the recipient cells.

### Intercellular transfer of mtDNA via EVs drives OXPHOS upregulation in CECs

**Fig. 3:**
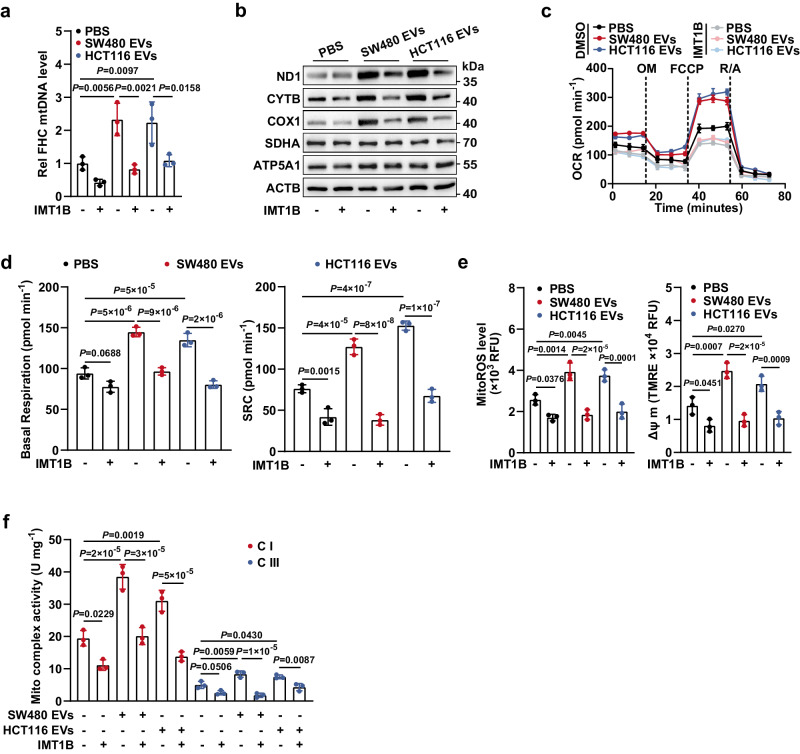
EV-induced elevation of mtDNA content is responsible for the enhanced OXPHOS in CECs. **a** FHC cells were educated with EVs derived from SW480 or HCT116 cells, in the presence or absence of IMT1B treatment (1 μM, 48 h). Then, the mtDNA content in FHC cells was determined. Rel, relative. *n* = 3 biological replicates. **b** Changes in expression levels of mtDNA-encoded proteins (ND1, CYTB, and COX1) and nuclear DNA-encoded mitochondrial proteins (SDHA and ATP5A1) in FHC cells. FHC cells were treated as described in **a**. The samples derive from the same experiment but different gels for SDHA, COX1, and ND1, another for ACTB, ATP5A1, and another for CYTB were processed in parallel. Changes in (**c**) OCR and (**d**) basal/spare respiratory capacities of FHC cells which were treated as described in **a**. SRC, spare respiratory capacity. *n* = 3 biological replicates. Changes in (**e**) mitochondrial ROS levels and ΔΨ m, and (**f**) activities of mitochondrial complex I and III in FHC cells. FHC cells were treated as described in **a**. *n* = 3 biological replicates. Data are means ± SD. One-way ANOVA with Tukey’s multiple comparisons test. Source data are provided as a Source Data file.

We then sought to determine whether the increase in the mtDNA copy number was required for the enhancement of mitochondrial respiration. To this end, FHC cells were incubated with SW480 or HCT116 cell-derived EVs and pretreated with or without IMT1B, which was rigorously indicated to be a specific inhibitor for reducing the mtDNA copy number^19^. qPCR analysis verified the efficiency of the IMT1B-induced decrease in the mtDNA content (Fig. 3a). Immunoblotting showed that mtDNA-encoded proteins ND1, CYTB, and COX1 but not the nuclear DNA-encoded proteins SDHA and ATP5A1, were significantly upregulated in recipient cells educated with CC cell-derived EVs, while treatment with IMT1B sharply reversed this phenomenon (Fig. 3b). Moreover, treatment with EVs also increased mitochondrial respiratory capacities, the level of mitochondrial ROS, ΔΨ m, and the activities of mitochondrial complexes in recipient cells, and these increases were totally offset by the IMT1B-mediated decrease in the mtDNA content, indicating that the elevated mtDNA content mediates the EV education-induced upregulation of OXPHOS in CECs (Fig. 3c–f).

**Fig. 4:**
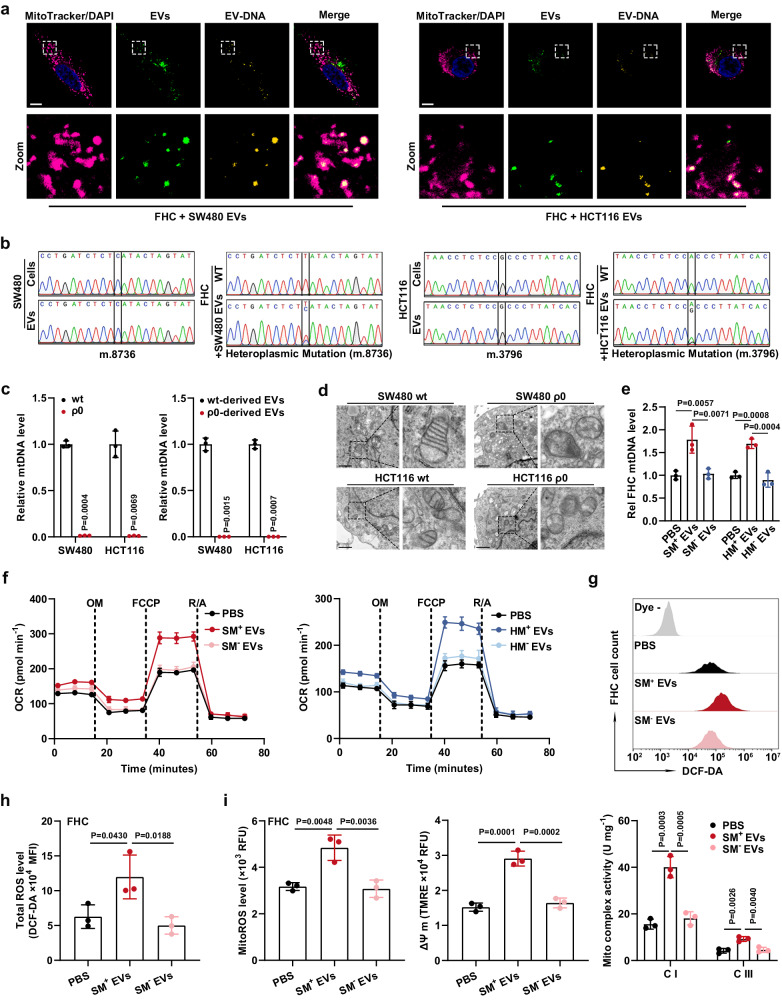
Transfer of EV-mtDNA leads to enhanced OXPHOS in CECs. **a** Confocal images of FHC cells incubated for 24 h with SW480- or HCT116-derived EVs. MitoTracker-labeled mitochondria (magenta), PKH67-labeled EVs (green), EtBr-labeled EV-DNA (yellow), and their co-localization (pale yellow). Scale bar, 10 μm. **b** Sanger sequencing was performed to detect point mutations in mtDNA purified from CC cells (SW480 and HCT116), their corresponding EVs, and FHC cells with or without tumor cell-derived EV education. **c** The relative mtDNA levels in SW480, SW480 ρ0, HCT116, and HCT116 ρ0 cells and their corresponding EVs were measured by qPCR. *n* = 3 biological replicates. **d** Mitochondrial structure in SW480, HCT116, and their corresponding ρ0 cells was observed using electron microscopy. Scale bar, 1 μm. **e**, **f** A subset of FHC cells was educated with mtDNA-sufficient EVs derived from wild-type SW480 cells (SM^+^ EVs) or HCT116 cells (HM^+^ EVs), and another subset of FHC cells was educated with mtDNA-depleted EVs derived from SW480 ρ0 cells (SM^−^ EVs) or HCT116 ρ0 cells (HM^−^ EVs). After incubation for 7 days, the (**e**) mtDNA copy number and (**f**) oxygen consumption rate (OCR) in these processed FHC cells were determined. Rel, relative. *n* = 3 independent experiments. **g** Representative flow cytometric plot and **h** statistical results of total ROS levels in FHC cells educated with SM^+^ EVs or SM^−^ EVs for 7 days. *n* = 3 biological replicates. **i** Changes in mitochondrial ROS levels, mitochondrial membrane potential (ΔΨ m), and the activities of mitochondrial complexes I and III in FHC cells educated with SM^+^ EVs or SM^−^ EVs for 7 days. *n* = 3 independent experiments. Data are means ± SD. Two-tailed t test (**c**). One-way ANOVA with Tukey’s multiple comparisons test (**e**, **h**, and **i**). Source data are provided as a Source Data file.

Notably, education with serum EVs derived from CC patients, instead of healthy individuals, significantly increased the mtDNA content in FHC cells, suggesting the potential mtDNA transfer by mtDNA-sufficient EVs (Supplementary Fig. 4a). In addition, the fold increase in the mtDNA level in recipient cells induced by tumor cell-derived EVs was not impaired by depletion of TFAM, a critical driver indispensable for mtDNA replication, excluding the possibility of endogenous induction (Supplementary Fig. 4b). EV tracking assays visually showed the transfer of EV-carried DNA into FHC cells (Supplementary Fig. 4c). More importantly, we observed the colocalization of EVs, EV-carried mtDNA, and the mitochondria of FHC cells, implying that EVs can fuse with recipient cells’ mitochondria, consequently transfer mtDNA into them (Fig. 4a). To further confirm this result, we examined mtDNA point mutations by amplifying mtDNA fragments, which were obtained from isolated mitochondria, and then performing Sanger sequencing. Intriguingly, only upon education with EVs derived from SW480 or HCT116 cells did mtDNA in FHC cells’ mitochondria exhibit the SW480 or HCT116 cell-type point mutations that were present in the CC cells and their corresponding EVs, providing strong evidence supporting the transfer of EV-mtDNA into mitochondria of CECs (Fig. 4b). Furthermore, FHC cells incubated with EVs derived from the murine CC cell line MC38 simultaneously exhibited intramitochondrial murine and human mtDNA, and the murine mtDNA fragments encoding Cox3, Cytb, and Nd2 were then verified by sequencing, further confirming the ability of CC cell-derived EVs to transfer mtDNA into mitochondria of CECs (Supplementary Fig. 4d, e). In addition, following coculture for indicated time periods, we did not observe the transfer of tumor cells’ mitochondria into recipient CECs, or increased mitochondrial mass/number in CECs, showing that EVs transport mtDNA, rather than the mitochondria themselves, into CECs (Supplementary Fig. 5a–e).

Next, we established mtDNA-depleted SW480 (SW480 ρ0) and HCT116 (HCT116 ρ0) cell lines to acquire EVs lacking mtDNA. The high efficiency of mtDNA depletion in both cell lines and their corresponding EVs was verified (Fig. 4c). Stable mtDNA deletion, resulting in a typical vacuolar structure in mitochondria, did not affect the cell proliferation, viability, and apoptosis, indicating that ρ0 cells retained their survival capacities (Fig. 4d and Supplementary Fig. 6a–g). Numerous ρ0 cell-derived EVs have also been identified (Supplementary Fig. 6h–j). We further investigated whether EV-transferred mtDNA can confer an enhanced mitochondrial respiratory phenotype on recipient cells that exhibited comparable uptake efficiency of EVs derived from ρ0 and wild-type CC cells (Supplementary Fig. 7a). Sanger sequencing assays demonstrated that ρ0 cell-derived EVs lost the ability to transfer detectable mtDNA into CECs (Supplementary Fig. 7b, c). Notably, the mtDNA copy number and the expression levels of mitochondrial ND1, CYTB, and COX1 were increased in recipient cells educated with mtDNA-rich EVs derived from wild-type SW480 cells (SM^+^ EVs) or HCT116 cells (HM^+^ EVs), but no effects were observed in FHC cells educated with mtDNA-deficient EVs derived from SW480 ρ0 cells (SM^−^ EVs) or HCT116 ρ0 cells (HM^−^ EVs) (Fig. 4e and Supplementary Fig. 7d). Moreover, phenotypic studies demonstrated significant increases in mitochondrial respiratory capacities, total and mitochondrial ROS levels, ΔΨ m, and the activities of mitochondrial complexes in recipient cells educated with mtDNA-rich EVs; however, depletion of mtDNA from EVs totally counteracted these changes, suggesting that mtDNA transfer via EVs is needed for the enhancement of mitochondrial function in CECs (Fig. 4f–i and Supplementary Fig. 7e–h).

In conclusion, these results indicate that exogenous mtDNA transfer is responsible for the EV-induced high-OXPHOS phenotype in CECs.

### EV-mtDNA-educated CECs contribute to tumor progression

**Fig. 5:**
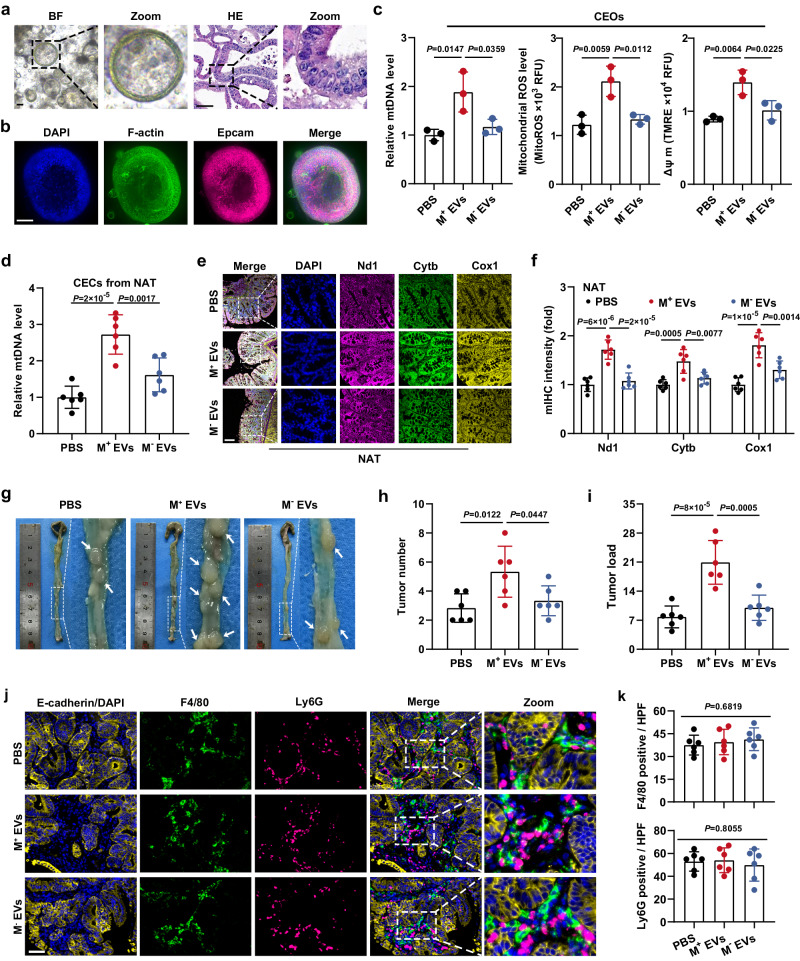
EV-mtDNA-educated CECs contribute to tumor progression. Murine colonic epithelial organoids (CEOs) were authenticated using (**a**) bright-field (BF) pictures, HE staining, and (**b**) immunofluorescence staining. Scale bar, 50 μm. **c** Changes in mtDNA content, mitochondrial ROS levels, and mitochondrial membrane potential (ΔΨ m) in CEOs incubated with murine mtDNA-enriched EVs (M^+^ EVs) or mtDNA-depleted EVs (M^−^ EVs). *n* = 3 independent experiments. **d** Determination of mtDNA content in CECs isolated from NAT in the murine orthotopic CC model treated with intraperitoneal injections of EVs derived from wild-type MC38 cells (M^+^ EVs) or mtDNA-depleted MC38 ρ0 cells (M^−^ EVs). *n* = 6 mice per group. **e** Representative images of mIHC staining for DAPI (blue), Nd1 (magenta), Cytb (green), and Cox1 (yellow) in sections from NAT in the murine orthotopic CC model treated with intraperitoneal injections of M^+^ EVs or M^−^ EVs. Scale bar, 100 μm. **f** The coexpression of Nd1, Cytb, and Cox1 was evaluated by quantification of the fluorescence intensity in **e**. *n* = 6 mice per group. **g** Representative gross view and statistical results of the (**h**) tumor number and (**i**) tumor load in the murine orthotopic CC model treated with intraperitoneal injections of M^+^ EVs or M^−^ EVs. *n* = 6 mice per group. **j** Representative images of mIHC staining for DAPI (blue), epithelial marker (E-cadherin, yellow), macrophage marker (F4/80, green), and neutrophil marker (Ly6G, magenta) in sections from tumors in the murine orthotopic CC model treated with M^+^ EVs or M^−^ EVs. **k** Statistical analysis of the number of F4/80-positive or Ly6G-positive cells per high power field (HPF) was then performed. Scale bar, 50 μm. *n* = 6 mice per group. Data are means ± SD. One-way ANOVA with Tukey’s multiple comparisons test. Source data are provided as a Source Data file.

Since organoids are frequently employed to imitate in vivo situations, we incubated murine colonic epithelial organoids (CEOs) with MC38-derived EVs. Organoid characterization was performed (Fig. 5a, b). Expectedly, CC cell-derived EVs dramatically increased the mtDNA content, mitochondrial proteins, ROS levels, and ΔΨ m in CEOs, which was significantly attenuated by EV-mtDNA depletion (Fig. 5c and Supplementary Fig. 8a), supporting the conclusion that EV-mtDNA transfer enhances OXPHOS in CECs. To further investigate whether CECs can take up tumor cell-derived EVs in vivo, we isolated primary CECs from mice receiving intraperitoneal injections of fluorescent DiD-labeled EVs derived from MC38 cells and clearly observed the uptake of exogenous EVs by these CECs by confocal microscopy (Supplementary Fig. 8b). Consistent with the in vitro results, in the AOM/DSS-induced murine orthotopic CC model, intraperitoneal injections of EVs derived from wild-type MC38 cells (M^+^ EVs) into tumor-bearing mice dramatically increased the mtDNA content and coexpression of Nd1, Cytb, and Cox1 in NAT, while injection of mtDNA-depleted EVs (M^−^ EVs) derived from MC38-ρ0 cells strongly attenuated this effect (Fig. 5d–f). However, no change occurred in the mtDNA copy number and the encoded protein levels extracted from tumor tissues (Supplementary Fig. 8c, d). Notably, tumor-bearing mice injected with M^+^ EVs but not those injected with M^−^ EVs exhibited an increased tumor number and tumor burden, implying that CECs educated with EV-mtDNA might in turn promote tumor progression (Fig. 5g–i).

It is necessary to point out that the severity of colonic inflammation evaluated by colon length, pathological scores, and the levels of pro-inflammatory cytokines was not affected by EV administration (Supplementary Fig. 8e–g). Meanwhile, macrophage and neutrophil infiltration in the tumors, as well as the content of colonic EVs, were unchanged upon EV injection (Fig. 5j, k and Supplementary Fig. 8h). These results suggest that colonic inflammation is not involved in the tumor-promoting effects of EVs used in moderation. Moreover, the experimental dose of EVs had no obvious pathological effect on other major organs of mice (Supplementary Fig. 9). The metabolic effects of EVs on extra-intestinal organs are unclear, but we speculate that they have minimal impact on OXPHOS in organs with intrinsic robust mitochondrial metabolism, such as the liver and brain. Moreover, the pathological environment surrounding the tumor may contribute to the regulatory function of EVs. However, these speculations warrant further research.

Next, another two human CC cell lines, RKO and HT29, were cocultured with FHC cells. The phenotypic assays suggested that FHC cells educated by mtDNA-sufficient EVs had the most pronounced promotive effects on the proliferation, migration, and invasion abilities of CC cells, which were weakened by EV-mtDNA depletion (Supplementary Fig. 10a–h). The same results were observed in SW480 and HCT116 cells, indicating a widespread phenomenon (Supplementary Fig. 11a–h). Thus, we conclude that CECs educated with EV-mtDNA promote malignant phenotypes in CC cells.

### TGFβ1 upregulation mediated by EV-mtDNA education is responsible for the CEC-mediated increase in tumor malignancy

**Fig. 6:**
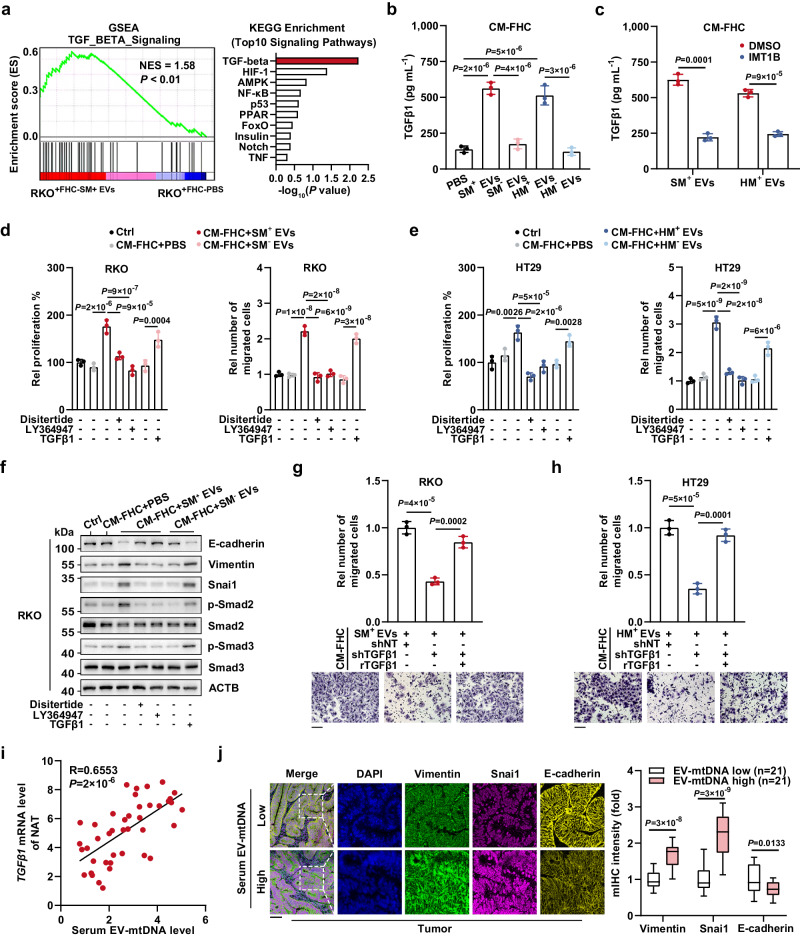
TGFβ1 upregulation by EV-mtDNA education results in CEC-enhanced tumor malignancy. **a** A 0.4-μm Transwell membrane was used to coculture RKO cells with FHC cells prestimulated with SM^+^ EVs or PBS. Then, the RKO cells were processed for transcriptome sequencing. GSEA using a two-sided permutation test and KEGG pathway enrichment analysis using a two-sided hypergeometric test were performed. **b** ELISAs were performed to quantify TGFβ1 in CM from FHC. *n* = 3 independent experiments. **c** TGFβ1 in CM from EV-educated FHC cells with or without IMT1B treatment (1 μM, 48 h) was quantified. *n* = 3 independent experiments. **d** RKO and **e** HT29 were incubated with CM from FHC cells pretreated with mtDNA-rich or mtDNA-depleted EVs and treated with disitertide (10 μM), LY364947 (1 μM), or TGFβ1 (1 ng mL^−1^). Then, the proliferation and migration abilities were determined. Rel, relative. *n* = 3 independent experiments. **f** The levels of phosphorylated Smad2/3 and EMT markers were examined. The samples derive from the same experiment but different gels for E-cadherin, Vimentin, and Snai1, another for ACTB, p-Smad2, another for Smad2, p-Smad3, and another for Smad3 were processed in parallel. **g**, **h** FHC cells expressing shNT or shTGFβ1 were infected with lentivirus expressing rTGFβ1, followed by education with SM^+^ EVs or HM^+^ EVs. Then, (**g**) RKO and (**h**) HT29 cells were incubated with CM from the processed FHC cells for 48 h. A Transwell migration assay was performed. Rel, relative. Scale bar, 100 μm. *n* = 3 independent experiments. **i** The correlation between the serum EV-mtDNA and *TGFβ1* mRNA levels in NAT from CC patients (*n* = 42) was analyzed using two-sided Pearson correlation analysis. **j** Representative images of mIHC staining in sections of tumor tissues from CC patients. Scale bar, 100 μm. The coexpression of EMT markers was evaluated. Data are means ± SD. The boxplots indicate median (center), 25th and 75th percentiles (bounds of box), and 2.5th and 97.5th percentiles (whiskers). Two-tailed t test (**c**, **j**). One-way ANOVA with Tukey’s multiple comparisons test (**b**, **d**, **e**, **g**, and **h**). Source data are provided as a Source Data file.

Next, RKO cells cocultured with FHC cells prestimulated with SM^+^ EVs (treated) or PBS (control) were processed for high-throughput transcriptome sequencing. GSEA of hallmark gene sets and KEGG pathway enrichment analyses showed significant enrichment of the TGFβ signaling pathway in the treated groups (Fig. 6a, Supplementary Data 1 and Supplementary Data 2). This was further confirmed by qPCR analysis of several TGFβ-related genes upon indicated treatment (Supplementary Fig. 12a). Given that the expression of TGFβ1, a major member of the TGFβ family, is positively correlated with tumor progression^20^, we hypothesized that EV-mtDNA education might upregulate TGFβ1 expression in CECs, subsequently activating the TGFβ signaling pathway in CC cells. In support of this hypothesis, the ELISA results demonstrated that education with mtDNA-rich EVs markedly increased the level of TGFβ1 in CM from FHC cells (CM-FHC) and that this increase was fully reversed by the depletion of mtDNA in EVs or by the IMT1B-mediated decrease in mtDNA in FHC cells (Fig. 6b, c). Moreover, treatment with CM from FHC cells incubated with mtDNA-rich EVs greatly promoted tumor cell proliferation, migration, and invasion, while the TGFβ1 inhibitors disitertide and LY364947 dramatically diminished this effect. Besides, exogenous TGFβ1 administration contributed to enhanced tumor malignancy (Fig. 6d, e and Supplementary Fig. 12b–e). Luciferase reporter assays confirmed the activation of the TGFβ signaling pathway in CC cells stimulated by exogenous TGFβ1 or incubated with CM from FHC cells pretreated with mtDNA-rich EVs, and this activation was abrogated by TGFβ pathway inhibitors or depletion of EV-mtDNA (Supplementary Fig. 12f, g). In accordance with the previous studies that showed the TGFβ/Smad signaling pathway as a key regulator of epithelial-mesenchymal transition (EMT)^21^, we found that the CM from FHC cells educated by EV-mtDNA induced a TGFβ1-dependent EMT phenotype in tumor cells, characterized by increased levels of phosphorylated Smad2/3, Vimentin, and Snai1 and decreased level of E-cadherin (Fig. 6f and Supplementary Fig. 12h).

We further knocked down TGFβ1 expression in FHC cells with a shRNA targeting TGFβ1 (shTGFβ1) and reconstituted TGFβ1 expression with shRNA-resistant TGFβ1 (rTGFβ1) (Supplementary Fig. 13a). Notably, upon intercellular communication through CM-FHC, TGFβ1 depletion in EV-educated FHC cells significantly inhibited tumor cell proliferation, migration, and invasion, while re-expression of rTGFβ1 in FHC cells fully restored the enhanced malignancy of CC cells (Fig. 6g, h and Supplementary Fig. 13b–f). TGFβ1 is secreted as an inactive complex and converted to an active form by THBS1 or ITGAV^22^. Our immunofluorescence assays revealed considerable amounts of THBS1 (cytosolic) and ITGAV (membrane) in FHC cells, implying that CECs have the potential to activate TGFβ1 (Supplementary Fig. 13g). Of note, exogenous TGFβ1 and CM from FHC cells pretreated with mtDNA-rich EVs synergistically promoted tumor progression (Supplementary Fig. 14a–d).

Furthermore, the clinical data of recruited CC patients showed that the serum EV-mtDNA level was positively correlated with the *TGFβ1* mRNA level in NAT and that tumor tissues from patients with high levels of serum EV-mtDNA exhibited an enhanced EMT phenotype (Fig. 6i, j). Collectively, these findings indicate that EV-mtDNA induces upregulation of TGFβ1 in CECs, thereby promoting EMT and the malignant progression of CC cells.

### ROS elevation induced by EV-mtDNA transfer promotes *TGFβ1* transcription via the NF-κB pathway in CECs

**Fig. 7:**
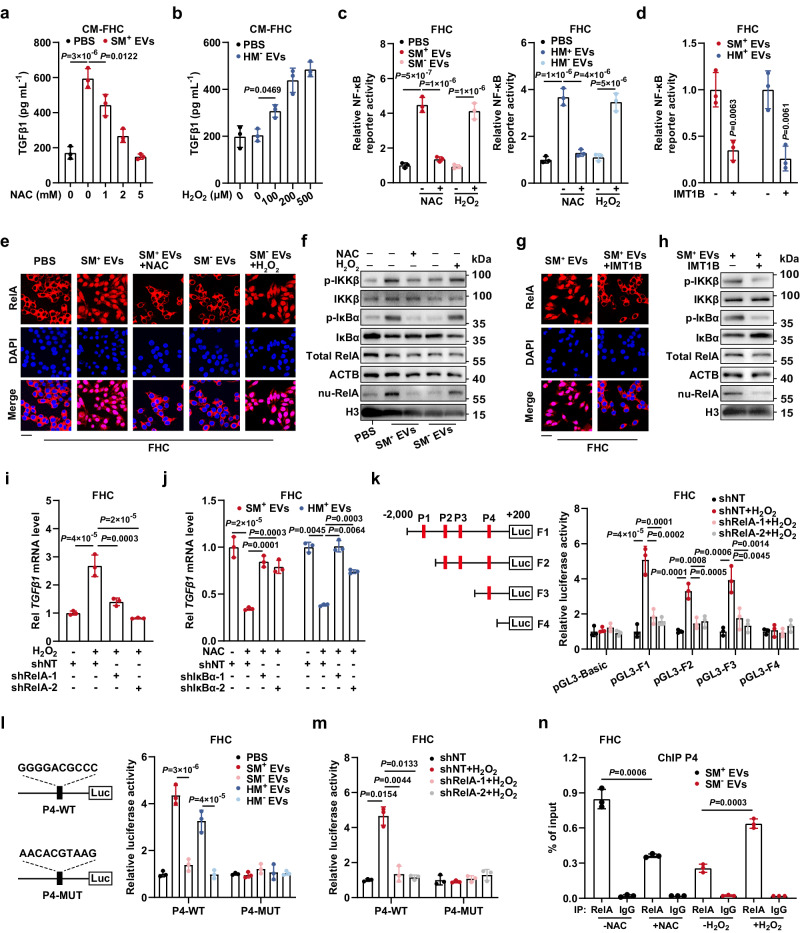
EV-mtDNA transfer promotes *TGFβ1* transcription via ROS-mediated activation of the NF-κB pathway. **a** ELISAs were performed to quantify TGFβ1 in CM from FHC. *n* = 3 independent experiments. **b** TGFβ1 in CM from FHC in the presence or absence of H_2_O_2_ stimulation was quantified. *n* = 3 independent experiments. **c** NF-κB reporter activity was measured. Subsets of FHC cells were treated with NAC (5 mM) or H_2_O_2_ (500 μM). *n* = 3 independent experiments. **d** NF-κB reporter activity in FHC with or without IMT1B treatment (1 μM) was measured. *n* = 3 independent experiments. The RelA activation was evaluated by (**e**) immunofluorescence and (**f**) immunoblotting. Scale bar, 30 μm. The RelA activation was evaluated by (**g**) immunofluorescence and (**h**) immunoblotting. Scale bar, 30 μm. **i** FHC cells expressing shNT or shRelA were stimulated with H_2_O_2_. *TGFβ1* mRNA levels were then determined. Rel, relative. *n* = 3 independent experiments. **j** FHC cells expressing shNT or shIκBα were treated with NAC. Subsequently, *TGFβ1* mRNA levels were determined. Rel, relative. *n* = 3 independent experiments. **k** The binding site truncation mutants inserted into the pGL3 vector were transfected into FHC cells. The luciferase activity was monitored. *n* = 3 independent experiments. **l** The luciferase activity of reporters containing the wild-type or mutated P4 binding site was determined. *n* = 3 independent experiments. **m** Luciferase reporter plasmids containing the wild-type or mutated P4 binding site were transfected into FHC cells. Then, luciferase activity was measured. *n* = 3 independent experiments. **n** ChIP assays were performed to evaluate RelA enrichment on P4. *n* = 3 independent experiments. As shown in **f** and **h**, the total protein samples derive from the same experiment but different gels for IKKβ, p-IκBα, and another for p-IKKβ, total RelA, ACTB, and IκBα were processed in parallel. The nuclear protein samples for detecting nu-RelA and H3 were processed on the same gel. Data are means ± SD. Two-tailed t test (**d**, **n**). One-way ANOVA with Tukey’s (**a**–**c**, **i**–**l**) or Games-Howell’s (**j**, **m**) multiple comparisons test (two-sided). Source data are provided as a Source Data file.

Following our discovery that EV-mtDNA transfer increases ROS production and TGFβ1 expression in recipient cells, we next investigated whether the elevated ROS was responsible for the upregulation of TGFβ1. Notably, the ROS scavenger N-acetyl-cysteine (NAC) counteracted the TGFβ1 upregulation induced by mtDNA-rich EVs, exogenous H_2_O_2_ raised the TGFβ1 level after the failure of mtDNA-depleted EVs to do so, and both effects were dose-dependent (Fig. 7a, b and Supplementary Fig. 15a). Luciferase reporter assays were then performed to monitor the activation of ROS-related signaling pathways according to previous reports^23^. The responsiveness of the luciferase reporters was verified in 293 T cells (Supplementary Fig. 15b). In recipient cells, mtDNA-sufficient EVs increased the activity of NF-κB and JNK, but mtDNA deficiency in EVs attenuated the activity of only NF-κB (Fig. 7c and Supplementary Fig. 15c, d). Moreover, the JNK inhibitor SP600125 showed no effect on TGFβ1 expression (Supplementary Fig. 15e). Additionally, NAC and IMT1B impeded NF-κB reporter activation induced by EV-mtDNA transfer, whereas exogenous H_2_O_2_ restored NF-κB activity (Fig. 7c, d). Consistent with these results, in FHC cells, the increased phosphorylation of IKKβ and IκBα and nuclear translocation of RelA caused by EV-mtDNA transfer were abolished by treatment with either NAC or IMT1B but were restored by H_2_O_2_ stimulation, revealing that the increase in ROS induced by EV-transferred mtDNA leads to NF-κB activation in CECs (Fig. 7e–h and Supplementary Fig. 15f–i).

We further examined whether activation of the NF-κB pathway mediates TGFβ1 upregulation. TCGA and GTEx database analyses via the GEPIA portal identified a strong positive correlation between *RelA* and *TGFβ1* in both NAT and completely normal colonic mucosa (Supplementary Fig. 16a). Importantly, the NF-κB inhibitor JSH-23 greatly impeded the H_2_O_2_-induced upregulation of TGFβ1 protein and mRNA expression (Supplementary Fig. 16b). The gradient inhibitory effect of JSH-23 on the NF-κB pathway was confirmed (Supplementary Fig. 16c). Furthermore, RelA depletion by shRNA abolished the H_2_O_2_-induced increases in TGFβ1 protein and mRNA expression, and knockdown of IκBα (an endogenous inhibitor of NF-κB) restored TGFβ1 expression impaired by NAC treatment in FHC cells, highlighting that NF-κB activation mediates ROS-induced TGFβ1 upregulation (Fig. 7i, j and Supplementary Fig. 16d, e).

Next, we observed a prominent interaction between RelA and the region upstream of the *TGFβ1* gene in colonic cells through Cistrome DB, indicating the potential role of RelA in the transcriptional regulation of *TGFβ1* (Supplementary Fig. 16f). Based on the four predicted RelA binding sites in the *TGFβ1* promoter identified via the AliBaba2.1 portal, we designed a series of truncated mutants of the *TGFβ1* promoter, which were inserted into the pGL3 vector, and determined their responsive activity in FHC cells under H_2_O_2_-stimulating conditions. All of the recombinant reporters except pGL3-F4 showed significant responsiveness to H_2_O_2_, and RelA depletion fully attenuated the increases in activity, suggesting that P4 is the major RelA binding site in the *TGFβ1* promoter (Fig. 7k). Furthermore, we constructed luciferase reporters containing the wild-type sequence of P4 (P4-WT) or the mutated sequence of P4 (P4-MUT). As expected, P4-WT exhibited markedly increased activity in FHC cells educated with mtDNA-rich EVs or treated with H_2_O_2_, while both EV-mtDNA depletion and RelA knockdown reversed this phenomenon. Notably, no change was observed in P4-MUT (Fig. 7l, m and Supplementary Fig. 16g). Chromatin immunoprecipitation (ChIP) assays further confirmed that EV-mtDNA transfer contributed to RelA enrichment on P4 in a ROS-dependent manner (Fig. 7n and Supplementary Fig. 16h). Taken together, these findings indicate that the EV-mtDNA transfer-induced increase in ROS fosters nuclear translocation of RelA, thereby directly promoting *TGFβ1* transcription in CECs.

**Fig. 8:**
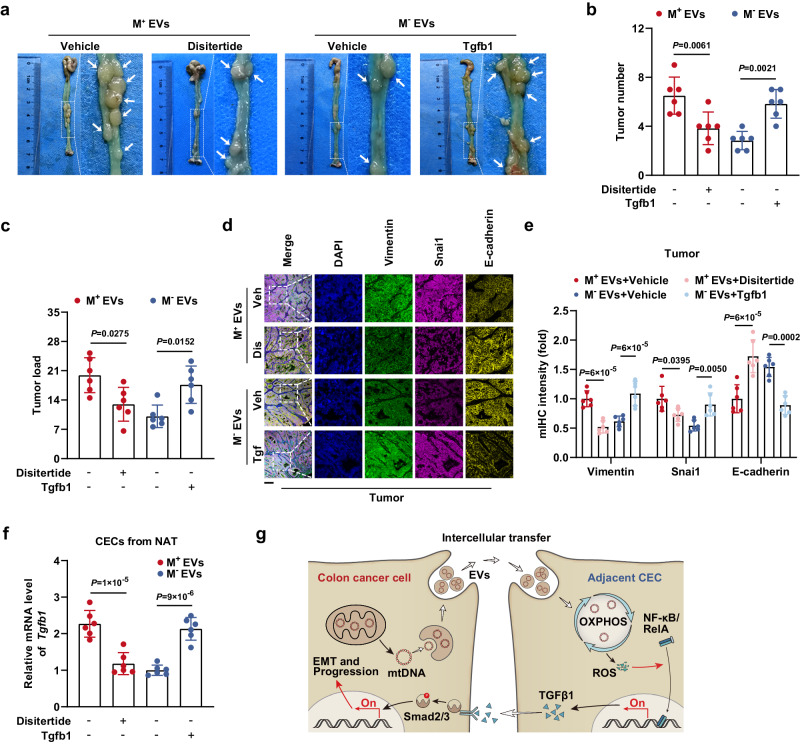
TGFβ1 expression driven by EV-mtDNA transfer promotes tumor progression in vivo. **a** Representative gross view and statistical results of (**b**) tumor number and (**c**) tumor load in mice in the disitertide- or Tgfb1-treated murine orthotopic CC model, which received intraperitoneal injections of M^+^ EVs or M^−^ EVs. *n* = 6 mice per group. **d** Representative images of mIHC staining for DAPI (blue), Vimentin (green), Snai1 (magenta), and E-cadherin (yellow) in sections from tumor tissues in the murine orthotopic CC model treated as indicated. Veh Vehicle, Dis Disitertide, Tgf Tgfb1. Scale bar, 100 μm. **e** The coexpression of E-cadherin, Vimentin, and Snai1 was then evaluated by quantification of the fluorescence intensity. *n* = 6 mice per group. **f** The *Tgfb1* mRNA levels in CECs isolated from NAT in the murine orthotopic CC model treated as indicated were measured. *n* = 6 mice per group. **g** Schematic model showing the mechanism by which the crosstalk between CC cells and normal CECs contributes to tumor progression. Data are means ± SD. One-way ANOVA with Tukey’s multiple comparisons test. Source data are provided as a Source Data file.

Consistent with the in vitro findings, the results of in vivo studies indicated that large decreases in the tumor number, tumor burden, and EMT phenotype were caused by the TGFβ1 inhibitor disitertide in M^+^ EV-educated mice and that significant increases in the tumor number, tumor burden, and EMT phenotype resulted from exogenous Tgfb1 administration in mice treated with M^−^ EVs (Fig. 8a–e). Additionally, as the malignant tumor phenotype was attenuated by disitertide and enhanced by Tgfb1, CECs from NAT exhibited corresponding decreases and increases, respectively, in the mtDNA copy number; coexpression of Nd1, Cytb, and Cox1; mitochondrial ROS level; and endogenous *Tgfb1* mRNA content (Fig. 8f and Supplementary Fig. 17a–c). These results demonstrated the existence of a positive feedback loop in the crosstalk between CC cells and adjacent CECs via EV-mtDNA and TGFβ1 that subsequently accelerates tumor progression (Fig. 8g).

## Discussion

Recent findings have highlighted molecular changes in tumor-adjacent “normal” tissues in several types of cancer. For instance, the tissues surrounding endometrial cancer express higher levels of tumor-promoting proteins than the matched tumors do^24^. In hepatocellular carcinoma, peritumoral liver tissues were found to exhibit a specific metabolic phenotype by proteomic analysis^25^. Our studies show that mtDNA content and ROS levels are increased in NAT relative to DT or completely normal colonic mucosa. Subsequently, functional assays revealed enhanced OXPHOS in CECs upon communication with CC cells. Mechanistically, the transfer of mtDNA via tumor-derived EVs upregulates the expression of mitochondrially encoded proteins, instead of nuclear-encoded components, leading to an increase in ROS levels, ΔΨ m, and the activities of mitochondrial complexes, the main generator of intracellular ROS^26^. One reasonable explanation for this is that enzymatic reactions during OXPHOS, without stimulation, are usually unsaturated, thus providing a considerable reserve capacity for respiratory chain enzymes^27^. When several core subunits, frequently encoded by mtDNA^28^, are elevated, the catalytic reserve capacity of other enzymes will adapt to the increased metabolic flux, raising the total respiratory level. One additional explanation is that the assembly of mitochondrial complexes is a multistage process: mtDNA-encoded and nuclear-encoded subunits initially merge into various intermediates with distinct proportions, and then these intermediates either assemble into complexes or participate in the formation of supercomplexes, which regulate the activity of individual complexes^28^. When the proportion of mtDNA-encoded and nuclear-encoded subunits changes, the assembly efficacy of intermediates may enhance, thereby affecting the formation and function of complexes. Another potential synergetic mechanism is that the activity of nuclear-encoded subunits could vary via post-translational modifications (PTMs). For instance, acetylating the nuclear-encoded complex II subunit SDHA modifies its activity^29^. EV-altered mitochondrial metabolism may change the levels of various metabolic intermediates, which could modulate protein activity via PTMs, such as acetylation and lactylation, influencing other modifications, such as SUMOylation^30^. Thus, the nuclear-encoded subunits with PTMs may collaborate with mtDNA-encoded counterparts to boost OXPHOS. These claims require further studies.

Considering ROS might trigger cell death under oxidative stress^31^, we evaluated the effect of CC cell-derived EVs on the viability of recipient cells and found that tumor cell-derived EVs did not influence the proliferative potential of CECs, suggesting that the ROS elevation caused by mtDNA transfer does not exceed a tolerable threshold in recipient cells. Besides mtDNA, several other OXPHOS-related molecules have been identified to be transferred by EVs. For instance, EV-mediated TFAM mRNA transfer in recipient cells prevented inflammation and mitochondrial damage^32^. Trophoblast-derived EVs rewired glucose metabolism in NK cells to maintain pregnancy via HLA-E^33^. We have previously reported that let-7a secreted via EVs regulates OXPHOS in CC cells^34^. This study reveals the metabolic crosstalk between CC cells and CECs occurring via EV-mtDNA transfer, adding to our knowledge of metabolic reprogramming within the intestinal TME.

We also identified EV-mtDNA-mediated CEC remodeling as a potential driver of CC progression in tumor-bearing mice. Follow-up studies demonstrated increased proliferation, migration, and invasion of CC cells cocultured with CECs prestimulated with EV-mtDNA, suggesting that CECs contribute to CC progression by secreting soluble factors. TGFβ1, a cancer-associated cytokine, has a dual role in tumorigenesis and tumor development. Several studies have revealed that TGFβ1 causes cell cycle arrest, which lowers cancer cell proliferation^35^. However, TGFβ1 is also thought to play an oncogenic role in a few types of tumors, such as gastric cancer and melanoma^36,37^. Our findings show that EV-mtDNA-educated CECs express higher levels of TGFβ1, which enhances EMT and malignant phenotypes in CC cells via the TGFβ/Smad signaling pathway, indicating that suppression of CEC-produced TGFβ1 may therapeutically help to inhibit CC cell proliferation and progression.

We further found that the increased TGFβ1 expression in CECs caused by the transfer of EV-mtDNA required ROS-mediated activation of the NF-κB pathway. The results of luciferase reporter assays and ChIP assays further confirmed the transcriptional activation of *TGFβ1* following the nuclear translocation of RelA. Although the duration of inducible NF-κB activation is strictly controlled under most conditions, constitutive activation of the NF-κB pathway is frequently observed during tumor development^38^. Here, we provide a possible explanation for this phenomenon: the elevated baseline level of intracellular ROS in CECs educated with tumor-derived EVs may contribute to constitutive NF-κB activation, thereby resulting in persistent upregulation of TGFβ1 expression.

Some intriguing issues call for further investigation. MtDNA is enriched in CC cell-derived EVs, but the mechanism by which mtDNA is selectively released into EVs remains obscure. We have demonstrated that EVs can deliver mtDNA to recipient mitochondria, which might involve membrane fusion between EVs and mitochondria, allowing EV cargo to enter the organelles. However, the detailed mechanisms of EV-organelle fusion, especially with mitochondria, remain elusive and warrant further investigation. Existing methods are still challenging to remove mtDNA from cells fully. Hence, more effective ρ0 cell construction technologies are needed for future mtDNA studies. Moreover, some kinds of cells that are critically dependent on mitochondrial energy supply or highly susceptible to mtDNA content changes are unlikely to survive as ρ0 cells^39^, necessitating the development of novel research methods for these cell types.

## Methods

### Cell culture

Human embryonic kidney cell line 293T (CL-0005), human CC cell lines SW480 (CL-0223B), HCT116 (CL-0096), RKO (CL-0196), HT29 (CL-0118), and SW620 (CL-0225B) were provided by Procell (Wuhan, China). Human normal colonic epithelial cells FHC and murine CC cells MC38 were kindly gifted from Dr. Jikun Li (Shanghai Jiao Tong University School of Medicine). Cells were cultured in DMEM supplemented with 10% FBS and antibiotics, at 37 °C and 5% CO_2_. The tumor cell lines devoid of mtDNA (ρ0) were established and cultured as described previously^40^. Briefly, parental cells were grown in a medium supplemented with 100 ng mL^−1^ EtBr, 50 µg mL^−1^ uridine, and 1 mM pyruvate for 3 months, followed by verification of mtDNA depletion using qPCR and electron microscopy. All cell lines were authenticated by STR profiling and routinely tested negative for mycoplasma contamination.

### Clinical samples

Serum and tissue samples from CC patients (female 32, male 50) ranging from 36 to 97 years old or healthy donors (female 8, male 11) ranging from 34 to 68 years old were obtained at Shanghai General Hospital. The gender was determined by self-reporting. Tumor tissue samples (T), Normal adjacent tissue samples (NAT, 2 to 5 cm from tumor margin), and distant colonic tissue samples (DT, >5 cm from tumor margin) were confirmed by two experienced pathologists^41,42^. This study was approved by the Ethics Committee of Shanghai General Hospital (2020SQ150). All participants provided informed consent, allowing for the use of their samples and the disclosure of information identifying individuals, such as exact age, sex, and medical center. More detailed clinical information on the participants is listed in Supplementary Table 1. This study did not provide participants with additional compensation.

### Plasmid transfection

Non-target control shRNA (shNT) and shRNA targeting TFAM, RelA, IκBα, and TGFβ1 were cloned into the pGMLV-hU6 lentiviral vector (Genomeditech, Shanghai, China). The shNT was generated with the control oligonucleotide 5’-TTCTCCGAACGTGTCACGT-3’. TFAM shRNA-1 target sequence: 5’-GTAAGTTCTTACCTTCGATTT-3’; TFAM shRNA-2 target sequence: 5’-GGCGGAGTGGCAGGTATATAA-3’; RelA shRNA-1 target sequence: 5’-CACCATCAACTATGATGAGTT-3’; RelA shRNA-2 target sequence: 5’-CGGATTGAGGAGAAACGTAAA-3’; IκBα shRNA-1 target sequence: 5’-GCCACACGTGTCTACACTTAG-3’; IκBα shRNA-2 target sequence: 5’-GATCACCAACCAGCCAGAAAT-3’; TGFβ1 shRNA target sequence: 5’-CAAGCAGAGTACACACAGCAT-3’. Using the pGMLV/hygro (+) vector, rTGFβ1 contains synonymous mutations of C372T, G375A, G378A, T381C, A384G, and A388T. After antibiotic selection for 14 days, the transfection efficiency of lentiviruses generated from 293T cells was validated by immunoblotting or ELISA.

### Isolation and identification of EVs

First, FBS was depleted of EVs by ultracentrifugation at 120,000 × *g* for 18 h (Optima L-100 XP; Beckman Coulter, Indianapolis, IN, USA). Cells were cultured in a medium supplemented with 10% EV-free FBS for 48 h. Then, the collected culture medium was sequentially centrifugated at 300 × *g* for 10 min, 2000 × *g* for 20 min, and 10,000 × *g* for 40 min to remove cell debris and shedding vesicles. Afterward, the harvested supernatant was filtrated through a 0.22-μm filter (Millipore, Billerica, MA, USA), followed by ultracentrifugation at 120,000 × *g* for 2 h at 4 °C. The pelleted EVs were washed in PBS and purified by ultracentrifugation at 120,000 × *g* for another 2 h. Serum EVs were isolated via differential ultracentrifugation in the same way.

An aliquot of EVs was fixed overnight with 2.5% glutaraldehyde and photographed using an H7500 transmission electron microscope (Hitachi, Tokyo, Japan). Another aliquot was enumerated by nanoparticle tracking analysis (NTA) on a ZetaView PMX110 (Particle Metrix, Meerbusch, Germany). Assessment of EV protein markers (CD63, ALIX, CD9, and TSG101) was performed by immunoblotting, whereas the endoplasmic reticulum marker Calnexin and the Golgi marker GM130 were included as negative controls.

### Characterization of EVs using sucrose gradient centrifugation

Isolated EVs were resuspended using sucrose stock solution to a final sucrose concentration of 82%, and placed at the bottom of an 82–10% sucrose gradient. Following ultracentrifugation at 100,000 × *g* for 16 h, six fractions (F1, 10–16%; F2, 22–28%; F3, 34–40%; F4, 46–52%; F5, 58–64%; F6, 70–82%) were collected, diluted in PBS, and centrifuged at 100,000 × *g* for 1 h. Pellets were then processed for immunoblotting and PCR analysis by volume normalization.

### Immunoblotting and ELISA

Total protein was obtained by RIPA buffer (New Cell & Molecular Biotech, Suzhou, China) and nuclear protein was extracted using the Nucleoprotein Extraction Kit (Sangon Biotech, Shanghai, China). Blots were incubated overnight at 4 °C with respective primary antibodies listed in Supplementary Table 2, followed by incubation with horseradish peroxidase (HRP)-conjugated secondary antibodies (1:5000; Affinity Biosciences, OH, USA) for 1 h at room temperature, then developed with high-sensitivity ECL (Vazyme, Nanjing, China). The protein bands were visualized using a Tanon 5200 Chemiluminescent Imaging System (Tanon, Shanghai, China). ACTB or histone H3 were used as loading controls. The validation information of antibodies is provided in the Reporting Summary. ELISA assays of human TGFβ1 and mouse IL1β/IL6/TNFα/IL17A (Multi Sciences, Hangzhou, China) were performed as per the manufacturer’s protocol using a Varioskan Multimode Microplate Reader (Thermo Fisher Scientific, Waltham, MA, USA).

### Real-time RT-PCR

RNA was extracted using TRIzol (Invitrogen, Camarillo, CA, USA) and reverse transcribed into cDNA with HiScript II Q RT SuperMix (Vazyme). Real-time RT-PCR was carried out on a QuantStudio 6 Flex Real-Time PCR System (Applied Biosystems, Foster City, CA, USA) using ChamQ Universal SYBR qPCR Master Mix (Vazyme). Primer sequences are available in Supplementary Table 3. Human ACTB and murine Actb served as internal controls.

### mIHC

Sections prepared from paraffin-embedded tissues were processed for mIHC staining with a PANO 4-plex IHC Kit (Panovue, Beijing, China) following the manufacturer’s instructions. Antigen retrieval was performed by microwaving the sections in 10 mM citrate buffer (pH 6.0). Then, endogenous peroxidase was inactivated by immersion of the sections in 3% hydrogen peroxide for 15 min. For each round of staining, the tissue sections were blocked in 10% normal goat serum for 30 min and then incubated with diluted primary antibodies (anti-Vimentin, 1:1,000; anti-Snai1, 1:500; anti-E-cadherin, 1:2,000; anti-ND1, 1:1,000; anti-COX1, 1:2,000; anti-CYTB, 1:1,000; anti-F4/80, 1:20,000; anti-Ly6G, 1:5,000) for 1 h at room temperature. The secondary antibodies and TSA system were provided in the kit. Finally, the slides were imaged by an SP8 confocal microscope (Leica Microsystems, Buffalo Grove, IL, USA) and analyzed by ImageJ v1.52n (National Institutes of Health, Bethesda, MD, USA).

### ChIP

ChIP assays were conducted according to the instructions of SimpleChIP Enzymatic Chromatin IP Kit (Cell Signaling Technology, Boston, MA, USA) with slight modifications. The ChIP-validated rabbit antibody against RelA was used for each assay, and normal rabbit IgG was used as a nonspecific control. RelA enrichment on the *TGFβ1* promoter was measured by real-time quantitative PCR, and 1% input was used for the qPCR reaction. ChIP primer sequences are given in Supplementary Table 3.

### Identification of mtDNA

Genomic DNA from cells or EV-DNA were isolated with FastPure Blood/Cell/Tissue/Bacteria DNA Isolation Mini Kit (Vazyme) or phenol/chloroform (Thermo Fisher Scientific), respectively. For long-range PCR, we used G5 High-Fidelity DNA Polymerase (EnzyArtisan, Shanghai, China) with 68 °C annealing temperature. A portion of the PCR products were then visualized by 0.8% agarose gel electrophoresis, the others were Sanger sequenced by Tsingke Biotechnology (Beijing, China) and analyzed using SnapGene v4.1.9 (GSL Biotech, Chicago, IL, USA). Moreover, after standard PCR using an annealing temperature of 56 °C with 2× Speeding Taq PCR Mix (EnzyArtisan), the 45 overlapping amplicons covering the entire mtDNA were resolved on 2% agarose gels. Each PCR was performed for 35 cycles on a ProFlex PCR System (Applied Biosystems). The PCR products were visualized using a Tanon 1600 Gel Imaging System (Tanon). For mtDNA quantification by real-time qPCR, we selected human *ND1* or murine *Nd4* as target mitochondrial genes, and nuclear genes (*ACTB* for human, *Rps18* for murine) as normalized controls. Primer sequences are provided in Supplementary Table 3 and Supplementary Table 4.

### Cell proliferation and viability analysis

Three thousand cells per well were seeded in 96-well plates and allowed to grow for indicated days. At the experimental endpoint, cells were incubated with a medium premixed with 10% CCK8 (Dojindo, Kumamoto, Japan) at 37 °C for 2 h, followed by monitoring absorbance at 450 nm. EdU assays were performed with a Cell-Light EdU Apollo567 In Vitro Kit (RiboBio, Guangzhou, China) according to the manufacturer’s instructions. The EdU incorporation rate, as captured by a DMi8 inverted microscope (Leica), reflected cell proliferation ability. Alternatively, SRB assays were used to determine cell proliferation: In a 96-well plate, seeded cells were fixed with 10% trichloroacetic acid overnight at 4 °C and stained with 0.4% SRB for 20 min at room temperature. After washing the plates with 1% acetic acid and solubilizing the residual dye with 10 mM Tris base solution for 15 min, the absorbance was monitored at 510 nm. We performed cell viability assays using the CellTiter-Glo 2.0 Assay Kit (Promega, Madison, WI, USA) and normalized the results by the cellular protein contents measured in parallel. The absorbance and chemiluminescence intensity were quantified using a Varioskan Multimode Microplate Reader (Thermo Fisher Scientific). Experiments were repeated independently three times.

### Cell apoptosis analysis

The cells were exposed to either vehicles or apoptosis inducers (1:1,000, Beyotime, Shanghai, China). The activity of Caspase3 was then probed by employing the Caspase3 Activity Assay Kit (Abcam, Cambridge, UK) on a Varioskan Multimode Microplate Reader (Thermo Fisher Scientific) after 6 h, and immunoblotting was conducted to gauge the levels of cleaved Caspase3 and cleaved PARP. Subsequently, the percentage of apoptotic cells was quantified by applying the TUNEL BrightGreen Apoptosis Detection Kit (Vazyme) on a DMi8 inverted microscope (Leica) after 12 h.

### Transwell migration and invasion assays

FHC cell-conditioned media were harvested by incubating with a serum-free medium for 2 days. To activate latent TGFβ1, transient acidification of conditioned media was performed^43^. Following incubation with conditioned media, CC cells (8 × 10^4^ cells per well) resuspended in serum-free medium were seeded into upper chambers containing 8.0-μm Transwell filters (Corning, NY, USA), with or without Matrigel (BD Biosciences, San Jose, CA, USA). Meanwhile, The lower chamber contained medium supplemented with 20% FBS. After 24 h, cells were fixed with 4% paraformaldehyde and stained with 0.4% crystal violet. Non-migrated or non-invaded cells were scraped off with cotton swabs before cell counting under a DMi8 inverted microscope (Leica). In parallel, equal amounts of cells were seeded onto filter-free plates to count the cell number after the same cell culture period, for further normalization of the relative migrated or invaded cells, avoiding the potential interference by cell proliferation. Each experiment was repeated three times.

### Immunofluorescence

Cells were pre-seeded onto poly-lysine-coated coverslips (WHB Scientific, Shanghai, China) in 12-well plates before fixation by 4% paraformaldehyde, with or without permeabilization by 0.2% Triton X-100 for 10 min. After blocking with 10% normal goat serum for 30 min, The coverslips were inverted onto a drop of primary antibody (anti-RelA, 1:800; anti-THBS1, 1:100; anti-ITGAV, 1:500) overnight at 4 °C. The next day, cells were incubated with CY3-conjugated goat anti-rabbit secondary antibody (1:200; Affinity Biosciences) for 1 h at room temperature while avoiding light, before being counterstained with DAPI (Servicebio, Wuhan, China). Fluorescence was observed by confocal microscopy (Leica).

### EV tracking assay

After the addition of EVs labeled with PKH67 (Umibio, Shanghai, China) for 24 h, recipient cells were fixed with 4% paraformaldehyde for 20 min and subsequently stained with iFluor 555-Phalloidin (MKBio, Shanghai, China) for 90 min according to the manufacturer’s protocol. The cells were then stained with DAPI. To visualize EV-mtDNA, EVs were labeled with EtBr (Sangon Biotech) before being added to the cell culture medium. To label mitochondria, recipient cells were incubated for 30 min with MitoTracker Red CMXRos (Beyotime). The cytoskeleton (magenta) or mitochondria (magenta), internalized EVs (green), EV-mtDNA (far-red), and nuclei (blue) were observed by confocal microscopy (Leica).

### Mitochondrial function assays

Recipient cells were incubated in the presence of tumor conditioned media (diluted 1:1 in serum-free media), or added with tumor cell-derived EVs at a dose of 3 × 10^3^ particles/cell every two days. Seven days later, the mitochondrial activity was detected using a Seahorse analyzer (Seahorse Bioscience, Billerica, MA, USA)^44^. Each well in the assay plates was verified to be sub-confluent and to have a comparable cell density on the day of assay, and the results were normalized by cell number. FHC cells seeded in an XF96 plate were sequentially treated with oligomycin, FCCP, and rotenone/antimycin A, which were added at 1 μM, 0.5 μM, and 0.5 μM respectively. Mitochondrial membrane potential was evaluated using a TMRE Mitochondrial Membrane Potential Assay Kit (BioVision, Milpitas, CA, USA). Respiratory complex activities were determined using the Mitochondrial Complex Activity Assay Kit (Solarbio, Beijing, China) based on the manufacturer’s instructions. The absorbance and fluorescence intensity was quantified using a Varioskan Multimode Microplate Reader (Thermo Fisher Scientific).

### Intracellular ROS measurement

Total ROS was measured with a ROS Assay Kit (Beyotime) on an Accuri C6 flow cytometer (BD Biosciences). Flow cytometry data in FCS format, generated by BD Accuri C6 Software v1.0.264.21 (BD Biosciences), were analyzed utilizing FlowJo v10.0.7 (BD Biosciences). Flow cytometric analyses were performed utilizing a uniform gating strategy, details of which are graphically delineated in the Supplementary Information. In parallel, mitochondrial ROS was detected by a Cell Meter Fluorimetric Mitochondrial Superoxide Activity Assay Kit Optimized for Microplate Reader (AAT Bioquest, Sunnyvale, CA, USA) according to the kit instructions. ROS levels in colon tissue were detected using the Tissue Reactive Oxygen Species Detection Kit (BestBio, Shanghai, China). A Varioskan Multimode Microplate Reader (Thermo Fisher Scientific) was used to measure the fluorescence intensity.

### Mitochondrial transfer assays

Mitochondria in SW480 and HCT116 cells were labeled using CellLight Mitochondria-RFP (Thermo Fisher Scientific) or MitoBright LT Green (Dojindo) as per the manufacturer’s protocol. FHC cells were then co-cultured with the labeled CC cells through the Transwell system for four days, followed by the detection of fluorescence signals in each cell line with a DMi8 inverted microscope (Leica). In parallel, FHC cells educated with EVs for the indicated time periods were harvested for assessment of mitochondrial mass using MitoTracker Green FM (Invitrogen) on a Varioskan Multimode Microplate Reader (Thermo Fisher Scientific).

### Dual luciferase assay

A series of Cignal Reporter Assay Kits (Qiagen, Germantown, MD, USA) were used for the assessment of the activation of signal transduction pathways as per the manufacturer’s protocol. Luciferase vectors containing wild-type or mutant-type *TGFβ1* promoter were transfected into indicated cells using Lipofectamine 3000 (Invitrogen). The luciferase activities were determined by a Dual Luciferase Reporter Assay System (Promega). The signals were detected using a Varioskan Multimode Microplate Reader (Thermo Fisher Scientific).

### RNA-seq

Total RNA was isolated using TRIzol (Invitrogen). Library preparation and sequencing were performed by Shanghai Personal Biotechnology (Shanghai, China). A NovaSeq 6000 platform (Illumina, San Diego, CA, USA) generated the raw data, which were then filtered with fastp v0.22.0 (OpenGene, GitHub). The filtered reads were mapped to the reference genome via HISAT2 v2.1.0 (Johns Hopkins University, Baltimore, MD, USA). Gene expression levels were calculated by HTSeq v0.9.1 (Python Package Index). Then difference expression of genes was analyzed by DESeq v1.38.3 (Fred Hutchinson Cancer Research Center, Seattle, WA, USA). ClusterProfiler v4.6.0 (Southern Medical University, Guangzhou, China) was used to carry out the enrichment analysis of the KEGG pathway. GSEA v4.1.0 (Broad Institute, Cambridge, MA, USA) was used for GSEA enrichment analysis.

### Organoid culture

Murine colonic epithelial organoids were cultured as described previously^45^. Briefly, colonic tissues were finely chopped and incubated with a dissociation solution at 4 °C for 30 min. After vigorous shaking and settling, the supernatant was moved and centrifuged for 5 min at 4 °C. The pellet was washed once with culture medium. Then, 500 crypts were embedded in 40 µL Matrigel and plated in a 24-well plate. The plate was incubated at 37 °C for 10 min, followed by the addition of 500 µL culture medium per well. For the initial three days, the medium was supplemented with 10 μg mL^−1^ each of metronidazole and ciprofloxacin.

The harvested organoids were fixed at 4 °C for 24 h using 4% paraformaldehyde, then dehydrated, embedded in paraffin blocks, cut into slices, and processed with standard HE-staining protocols. For immunofluorescence staining, organoids were fixed with 4% paraformaldehyde for 20 min at 4 °C, permeabilized with 0.2% Triton X-100 for 20 min at room temperature, and blocked with 1% BSA for 30 min, followed by incubation with the primary antibody overnight at 4 °C. Organoids were then stained for 1 h at room temperature using Alexa Fluor 488 Phalloidin (Thermo Fisher Scientific), secondary antibody, and DAPI. After incubation with indicated EVs for seven days, organoids were obtained for analysis of mtDNA content and levels of mitochondrial proteins, ROS, and membrane potential as described above.

### Establishment and treatment of murine orthotopic CC models

All animal experiments were approved by the Shanghai General Hospital, Shanghai Jiao Tong University School of Medicine Animal Care and Use Committee (IACUC No. 2022AW015). The ethics committee mandates that the maximal tumor size must not exceed 15 mm in diameter. We confirm that the maximal tumor size was not surpassed during our study. Euthanasia was administered to the mice upon reaching the experimental endpoint to obtain tissue specimens for subsequent analysis. Mice housed in SPF conditions were maintained in a temperature-regulated facility at 22 °C with 50–60% humidity, adhering to a 12-hour light/dark cycle. They had ad libitum access to Picolab Rodent Diet 20 (product code #5053). Sex was not considered in this study. Male mice were used due to the consistency of their hormone levels, which could reduce variability in experimental results.

The orthotopic CC model was established essentially as previously described^46^. Briefly, 6- to 8-week-old male *C57BL/6J* mice (GemPharmatech, Nanjing, China), assigned to groups randomly, received a single intraperitoneal injection of azoxymethane (AOM, 10 mg kg^−1^ body weight). Meanwhile, mice were given 2.5% dextran sulfate sodium (DSS) in drinking water for 5 days, followed by 14 days of regular water. This cycle was repeated three times in total. EV administration was initiated at day 30 post injection of AOM. Mice intraperitoneally received 3 × 10^9^ EVs in a 50-µL volume of PBS, once every 3 days for a total of 60 days. Control mice were administered the same volume of sterile PBS. At the same time, a subset of mice received intraperitoneal injection of disitertide (100 μg/mouse) or exogenous Tgfb1 (200 ng/mouse). All mice were sacrificed at 60 days after the first EV injection. Tumor load was calculated by adding the diameters of all tumors in a given mouse^47^. Colon inflammation scores were calculated as previously described^48^.

In parallel, fluorescent dye-labeled EVs were injected into another subset of mice in the same way mentioned above, to confirm exogenous EV uptake by mouse CECs. In brief, EVs were labeled with DiD (1:100, Beyotime) for 30 min at 37 °C and then washed by ultracentrifugation. For control, an identical volume of DiD working solution without EVs was treated in the same manner. After resuspension in PBS, EVs (3 × 10^9^ particles/mouse) along with control samples (only DiD) were intraperitoneally injected into mice once every 3 days for a total duration of 60 days. At the endpoint of the experiment, mouse CECs were isolated and stained with iFluor 555-Phalloidin. Exogenous EVs (far-red) and cytoskeleton (red) were observed by confocal microscopy (Leica). Given the potential toxicity of DiD, DiD-labeled EVs were strictly used for EV uptake studies and were not used for phenotypic or functional studies.

### Isolation of primary mouse CECs

Isolation of CECs was performed as described^49^. In short, colons were cut open longitudinally, flushed using PBS, and cut into 2-3 mm pieces, followed by incubation in HBSS containing 30 mM EDTA for 100 min at 4 °C. After immersion in fresh HBSS, pieces of colons were rocked carefully for 5 min and filtrated through a 40-μm cell strainer (BD Biosciences). Then, Crypts collected by centrifugation were digested using TrypLE Express (Invitrogen) for 8 min at 37 °C. Dissociated CECs were further purified through a 20-μm cell strainer (BD Biosciences) and washed with culture medium several times.

### Statistics and reproducibility

The two-tailed Student’s *t* test was used for two-group comparisons, while a one-way ANOVA test followed by Tukey or Games-Howell (when variances were not homogeneous) post-hoc test was employed for multiple group comparisons. To compare the proliferative abilities between wild-type and ρ0 cells, we conducted a two-way ANOVA. Comparison of mtDNA content among tumor, NAT, and paired distant colonic mucosa obtained from CC patients was made by Friedman test. The Chi-square test was utilized to compare categorical variables. Pearson correlation analysis was performed to evaluate correlations between two variables. Data are presented as means ± SD of at least three independent experiments. The boxplots indicate median (center), 25th and 75th percentiles (bounds of box), and 2.5th and 97.5th percentiles (whiskers). *P* < 0.05 was considered significant. No statistical method was used to predetermine sample size. No data were excluded from the analyses. The experimental procedures were conducted following a randomized design. The investigators were not blinded to allocation during experiments and outcome assessment. In the representative experiments presented, each experiment was repeated independently three times with similar results. Statistical analyses were performed using SPSS Statistics v25.0 (IBM, Chicago, IL, USA) and GraphPad Prism v8.4.3 (GraphPad Software, San Diego, CA, USA).

### Reporting summary

Further information on research design is available in the Nature Portfolio Reporting Summary linked to this article.

### Supplementary information

Supplementary Information

Peer Review File

Description of Additional Supplementary Files

Supplementary Data 1

Supplementary Data 2

Reporting Summary

### Source data

Source Data

## Data Availability

The RNA-seq data generated in this study have been deposited in the GEO database under accession code GSE233326. The correlation analyses for *RelA* and *TGFβ1* expression were conducted using data from the TCGA-COAD dataset obtained from the TCGA database, as well as the Colon-Sigmoid and Colon-Transverse datasets from the GTEx database via GEPIA, an interactive web server accessible at http://gepia.cancer-pku.cn. The interaction analysis between transcription factors and the *TGFβ1* promoter utilized publicly accessible platforms: Cistrome DB and AliBaba2.1 (http://gene-regulation.com/pub/programs/alibaba2/index.html). Users do not need to specify any particular dataset when using these two platforms. Instead, the platforms perform automatic analyses by synthesizing characteristics from the entire database. Source data are provided with this paper. Uncropped gels are presented in the Source Data file. Source data are provided with this paper.
