# Supplementary material for: Mitochondrial genome transfer drives metabolic reprogramming in adjacent colonic epithelial cells promoting TGFβ1-mediated tumor progression
Source: Nat Commun. 2024 Apr 30;15:3653. doi: 10.1038/s41467-024-48100-y (PMC11061154; doi:10.1038/s41467-024-48100-y)
Supplement: Supplementary file 1 — Supplementary Information [file 41467_2024_48100_MOESM1_ESM.pdf]

Supplementary information for:

Mitochondrial genome transfer drives metabolic reprogramming in adjacent colonic epithelial cells promoting TGFβ1-mediated tumor progression

Bingjie Guan, Youdong Liu, Bowen Xie, Senlin Zhao, Abudushalamu Yalikun, Weiwei Chen, Menghua Zhou, Qi Gu, and Dongwang Yan

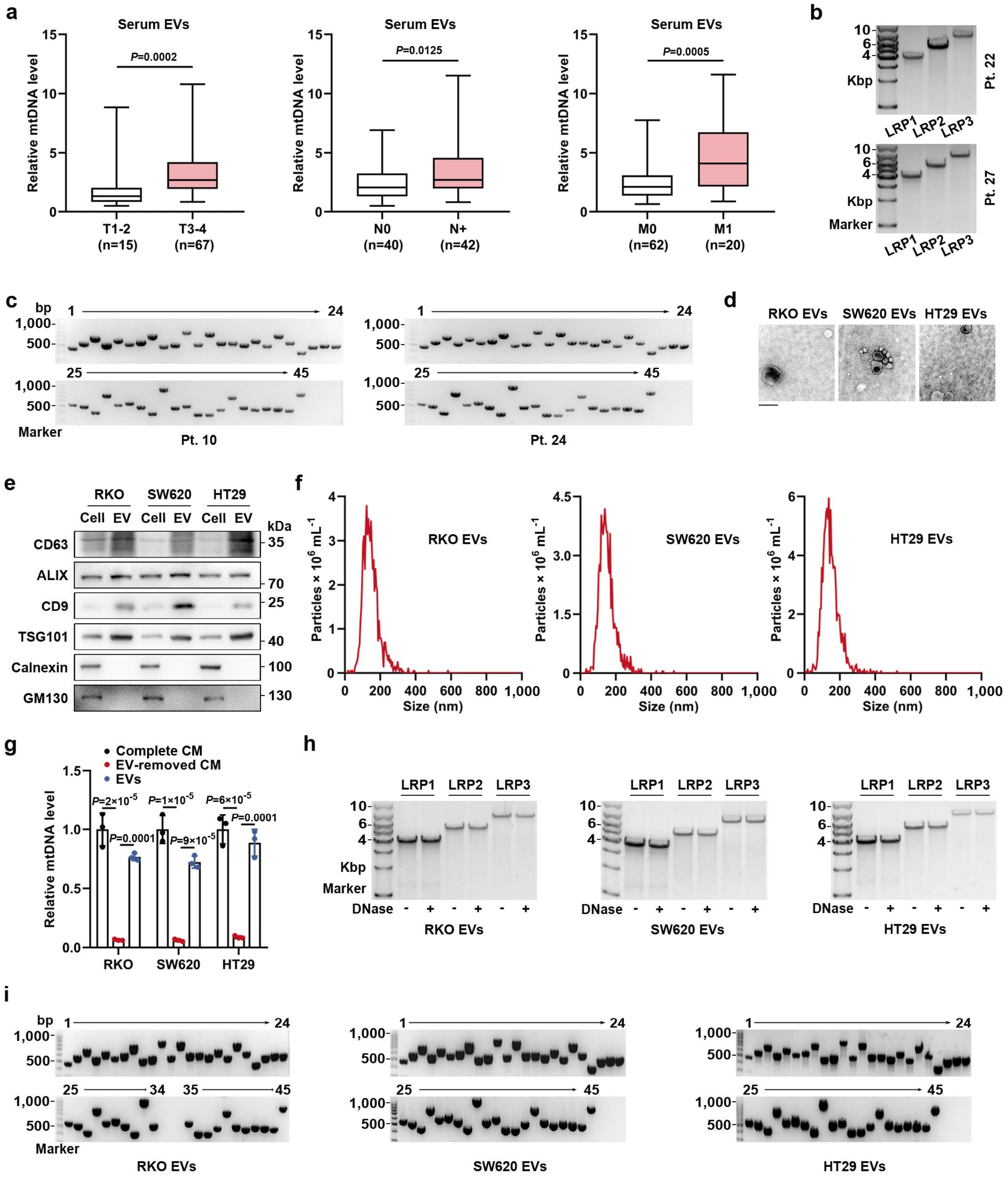

Supplementary Fig. 1. Complete mtDNA is abundant in EVs derived from CC cells. a The relative mtDNA levels in serum EVs from CC patients with different TNM stages were determined by qPCR targeting the ND1 gene. **b** Representative agarose gel electrophoresis pictures of long-range PCR with three amplicons covering the entire mtDNA obtained from serum EVs of patient 22 and patient 27. **c** Representative gel electrophoresis images of 45

overlapping amplicons encoding the whole mtDNA isolated from serum EVs of patient 10 and patient 24. **d-f** Identification of EVs derived from RKO, SW620, or HT29 cells via (**d**) electron microscopy, (**e**) immunoblotting, and (**f**) nanoparticle tracking analysis. The samples derive from the same experiment but different gels for GM130, ALIX, another for Calnexin, TSG101, and CD9, and another for CD63 were processed in parallel. Scale bar, 200 nm. **g** The levels of mtDNA in CC cell-derived total CM, EV-removed CM, and corresponding isolated EVs were examined by qPCR. n = 3 independent experiments. **h** Representative agarose gel electrophoresis pictures of long-range PCR using three amplicons covering the entire mtDNA isolated from CC cell-derived EVs pre-treated with or without DNase. **i** Representative gel electrophoresis images of 45 overlapping amplicons encoding the whole mtDNA purified from CC cell-derived EVs. Data are means ± SD. The boxplots indicate median (center), 25th and 75th percentiles (bounds of box), and 2.5th and 97.5th percentiles (whiskers). Two-sided Mann-Whitney test (**a**). One-way ANOVA with Tukey's multiple comparisons test (**g**). Source data are provided as a Source Data file.

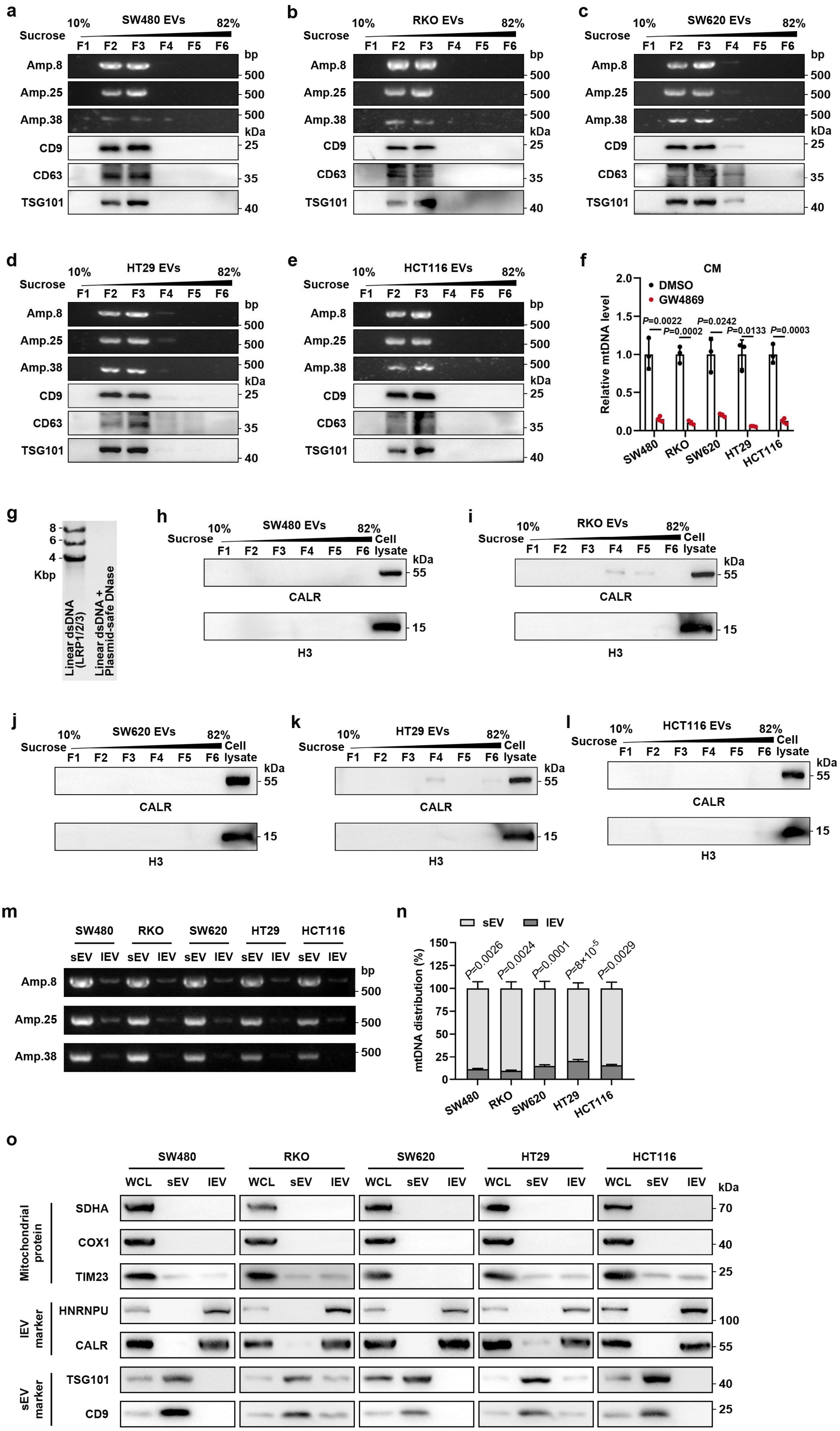
a
SW480 EVs
Sucrose 10% 82%
F1 F2 F3 F4 F5 F6
Amp.8
Amp.25
Amp.38
CD9
CD63
TSG101
bp 500 500 500
kDa 25 35 40
b
RKO EVs
c
SW620 EVs
d
HT29 EVs
e
HCT116 EVs
f
CM
DMSO
GW4869
Relative mtDNA level
2.0 1.5 1.0 0.5 0.0
P=0.0022
P=0.0002
P=0.0242
P=0.0133
P=0.0003
SW480 RKO SW620 HT29 HCT116
g
8 6 4
Kbp
Linear dsDNA (LRP1/2/3)
Linear dsDNA + Plasmid-safe DNase
h
SW480 EVs
Cell lysate
CALR
H3
kDa 55 15
i
RKO EVs
j
SW620 EVs
k
HT29 EVs
l
HCT116 EVs
m
SW480 RKO SW620 HT29 HCT116
sEV lEV
Amp.8
Amp.25
Amp.38
bp 500
n
sEV lEV
mtDNA distribution (%)
150 125 100 75 50 25 0
P=0.0026
P=0.0024
P=0.0001
P=8×10^-5
P=0.0029
o
WCL sEV lEV
Mitochondrial protein
SDHA
COX1
TIM23
lEV marker
HNRNPU
CALR
sEV marker
TSG101
CD9
kDa 70 40 25 100 55 40 25

Supplementary Fig. 2. Characterization of the CC cell-derived EVs. a-e EVs derived from CC cells were separated on a sucrose gradient. Immunoblotting and gel electrophoresis analysis were used to determine the relative levels of sEV markers and mtDNA among the fractions. The samples were derived from the same experiment and processed on the same gel. **f** qPCR analysis was used to evaluate the relative levels of mtDNA secreted by DMSO- or GW4869-treated CC cells. n = 3 independent experiments. **g** The linear products of long-range PCR were used to verify the degradative activity of plasmid-safe DNase toward linear dsDNA. **h-l** Immunoblotting of EV proteins in a sucrose gradient for contamination markers. The samples were derived from the same experiment and processed on the same gel. **m, n** The mtDNA content in sEVs and lEVs was compared by (**m**) gel electrophoresis and (**n**) qPCR analysis. n = 3 independent experiments. **o** Immunoblotting was employed to detect the indicated proteins in sEVs and lEVs. The samples derive from the same experiment but different gels for SDHA, COX1, and TIM23, another for HNRNPU, CALR, TSG101, and CD9 were processed in parallel. WCL, whole cell lysate. Data are means ± SD. Two-tailed t test (**f, n**). Source data are provided as a Source Data file.

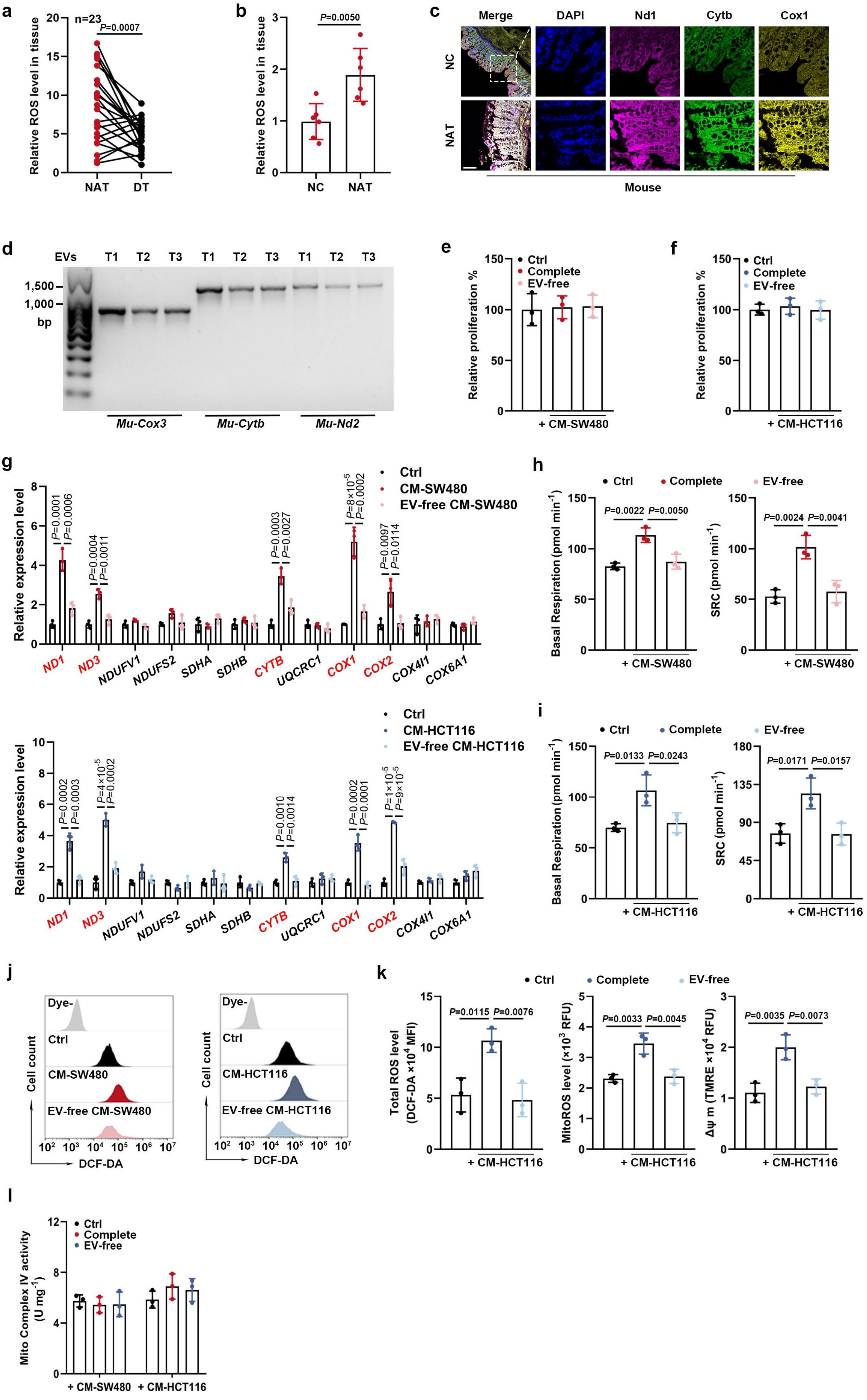
a
n=23
P=0.0007
Relative ROS level in tissue
NAT
DT
b
P=0.0050
Relative ROS level in tissue
NC
NAT
c
Merge
DAPI
Nd1
Cytb
Cox1
NC
NAT
Mouse
d
EVs
T1
T2
T3
1,500
1,000
bp
Mu-Cox3
Mu-Cytb
Mu-Nd2
e
Ctrl
Complete
EV-free
Relative proliferation %
+ CM-SW480
f
Ctrl
Complete
EV-free
Relative proliferation %
+ CM-HCT116
g
Ctrl
CM-SW480
EV-free CM-SW480
Relative expression level
P=0.0001
P=0.0006
P=0.0004
P=0.0011
P=0.0003
P=0.0027
P=8×10^-5
P=0.0002
P=0.0097
P=0.0114
ND1
ND3
NDUFV1
NDUFS2
SDHA
SDHB
CYTB
UQCRC1
COX1
COX2
COX4I1
COX6A1
Ctrl
CM-HCT116
EV-free CM-HCT116
Relative expression level
P=0.0002
P=0.0003
P=4×10^-5
P=0.0002
P=0.0010
P=0.0014
P=0.0002
P=0.0001
P=1×10^-5
P=9×10^-5
ND1
ND3
NDUFV1
NDUFS2
SDHA
SDHB
CYTB
UQCRC1
COX1
COX2
COX4I1
COX6A1
h
Ctrl
Complete
EV-free
Basal Respiration (pmol min^-1)
P=0.0022
P=0.0050
+ CM-SW480
SRC (pmol min^-1)
P=0.0024
P=0.0041
+ CM-SW480
i
Ctrl
Complete
EV-free
Basal Respiration (pmol min^-1)
P=0.0133
P=0.0243
+ CM-HCT116
SRC (pmol min^-1)
P=0.0171
P=0.0157
+ CM-HCT116
j
Dye-
Ctrl
CM-SW480
EV-free CM-SW480
Cell count
DCF-DA
Dye-
Ctrl
CM-HCT116
EV-free CM-HCT116
Cell count
DCF-DA
k
Ctrl
Complete
EV-free
Total ROS level (DCF-DA ×10^4 MFI)
P=0.0115
P=0.0076
+ CM-HCT116
MitoROS level (×10^3 RFU)
P=0.0033
P=0.0045
+ CM-HCT116
Δψ m (TMRE ×10^4 RFU)
P=0.0035
P=0.0073
+ CM-HCT116
l
Ctrl
Complete
EV-free
Mito Complex IV activity (U mg^-1)
+ CM-SW480
+ CM-HCT116

Supplementary Fig. 3. CECs display increased OXPHOS upon EV-dependent communication with CC cells. a Comparison of ROS levels in NAT and paired DT samples obtained from CC patients. **b** ROS levels in normal colonic mucosa or NAT acquired from healthy mice (negative control, NC) or the AOM/DSS-induced murine orthotopic CC model. n = 6 mice per group. **c** Representative images of multiplex immunohistochemistry (mIHC) staining for DAPI (blue), Nd1 (magenta), Cytb (green), and Cox1 (yellow) in sections from normal colonic mucosa or NAT acquired from healthy mice or the AOM/DSS-induced murine orthotopic CC model. Scale bar, 100 μm. **d** The murine mitochondrial genes in EVs derived from primary murine CC cells were determined. **e, f** Changes in proliferative potential of FHC cells incubated with (**e**) SW480 or (**f**) HCT116 cell-derived complete CM or EV-removed CM. n = 3 independent experiments. **g** Changes in mRNA expression levels of mtDNA-encoded (red) and nuclear-encoded (black) mitochondrial subunits in FHC cells incubated with SW480 or HCT116 cell-derived complete CM or EV-removed CM. n = 3 independent experiments. **h, i** Changes in basal and spare respiratory capacities of FHC cells incubated with (**h**) SW480 or (**i**) HCT116 cell-derived complete CM or EV-removed CM. SRC, spare respiratory capacity. n = 3 independent experiments. **j** Representative flow cytometric plots of total ROS levels in FHC cells educated with SW480 or HCT116 cell-derived complete CM or EV-free CM. **k** Changes in total/mitochondrial ROS levels and mitochondrial membrane potential (ΔΨ m) in FHC cells incubated with HCT116-derived complete CM or EV-free CM. n = 3 independent experiments. **l** Changes in activities of mitochondrial complex IV in FHC cells incubated with SW480 or HCT116 cell-derived complete CM or EV-removed CM. n = 3 independent experiments. Data are means ± SD. Paired t test (**a**). Two-tailed t test (**b**). One-way ANOVA with Tukey's multiple comparisons test (**e-i**, **k,** and **l**). Source data are provided as a Source Data file.

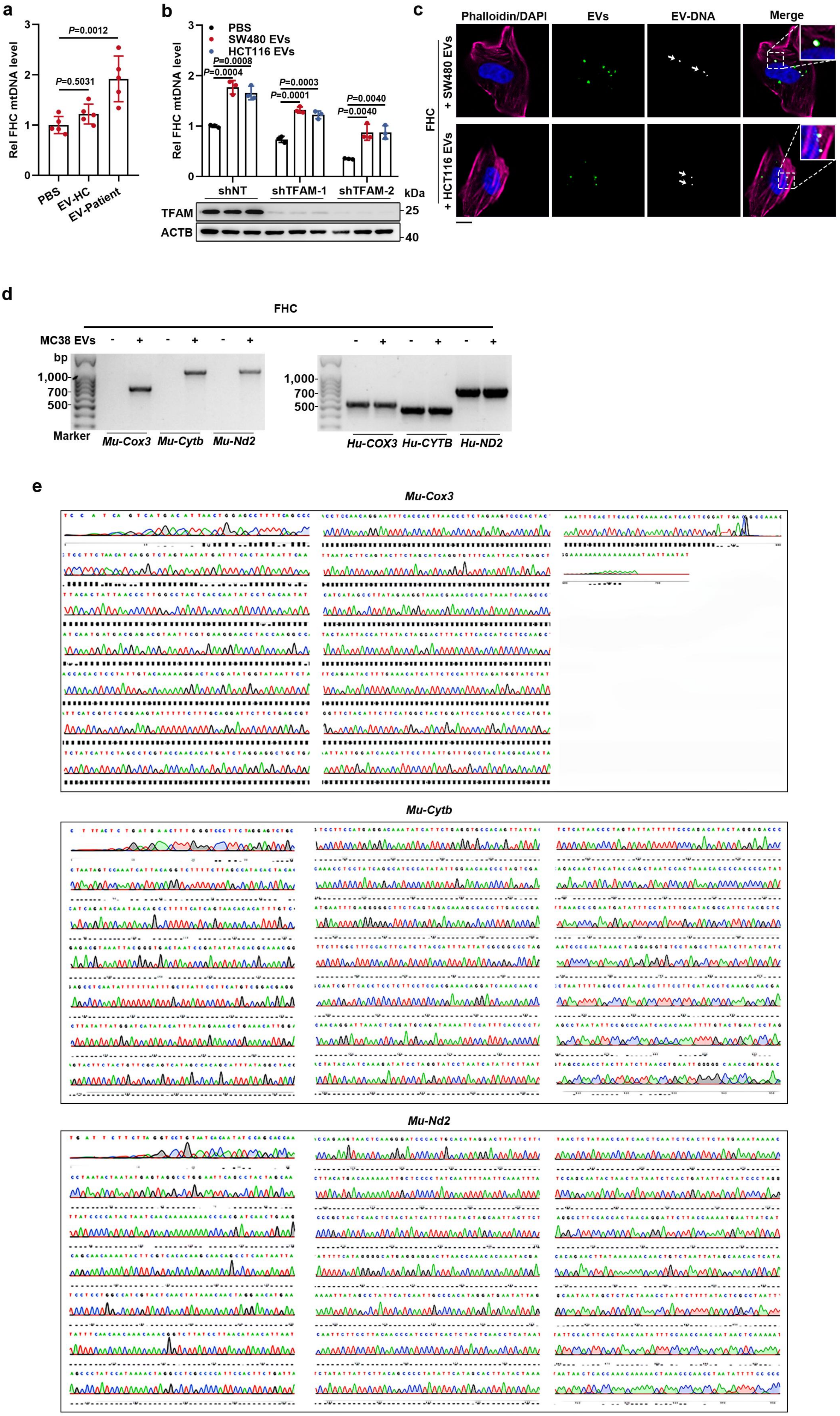
a
Rel FHC mtDNA level
P=0.0012
P=0.5031
PBS
EV-HC
EV-Patient
b
PBS
SW480 EVs
HCT116 EVs
Rel FHC mtDNA level
P=0.0008
P=0.0004
P=0.0003
P=0.0001
P=0.0040
P=0.0040
shNT
shTFAM-1
shTFAM-2
kDa
TFAM
25
ACTB
40
c
Phalloidin/DAPI
EVs
EV-DNA
Merge
+ SW480 EVs
+ HCT116 EVs
FHC
d
FHC
MC38 EVs
bp
1,000
700
500
Marker
Mu-Cox3
Mu-Cytb
Mu-Nd2
Hu-COX3
Hu-CYTB
Hu-ND2
e
Mu-Cox3
Mu-Cytb
Mu-Nd2

Supplementary Fig. 4. Transfer of exogenous mtDNA into CECs via CC cell-derived EVs. **a** FHC cells were educated with EVs derived from healthy control (HC) or CC patient serum, followed by determination of the intracellular mtDNA levels. Rel, relative. n = 5 independent experiments. **b** FHC cells stably expressing shNT or shTFAM were educated with EVs derived from SW480 or HCT116, followed by detection of the intracellular mtDNA content. Rel, relative. The samples were derived from the same experiment and processed on the same gel. n = 3 biological replicates. **c** Transfer of EV-DNA derived from SW480 or HCT116 cells into FHC cells was examined by confocal microscopy. Phalloidin (magenta)/DAPI (blue), EVs (green), EV-DNA (white). Scale bar, 10 μm. **d** The murine and human mitochondrial genes in FHC cells were determined, in the presence or absence of education with MC38-derived EVs. **e** After education with MC38-derived EVs, the murine mitochondrial genes observed in FHC cells were further confirmed by Sanger sequencing. Data are means ± SD. One-way ANOVA with Tukey's multiple comparisons test. Source data are provided as a Source Data file.

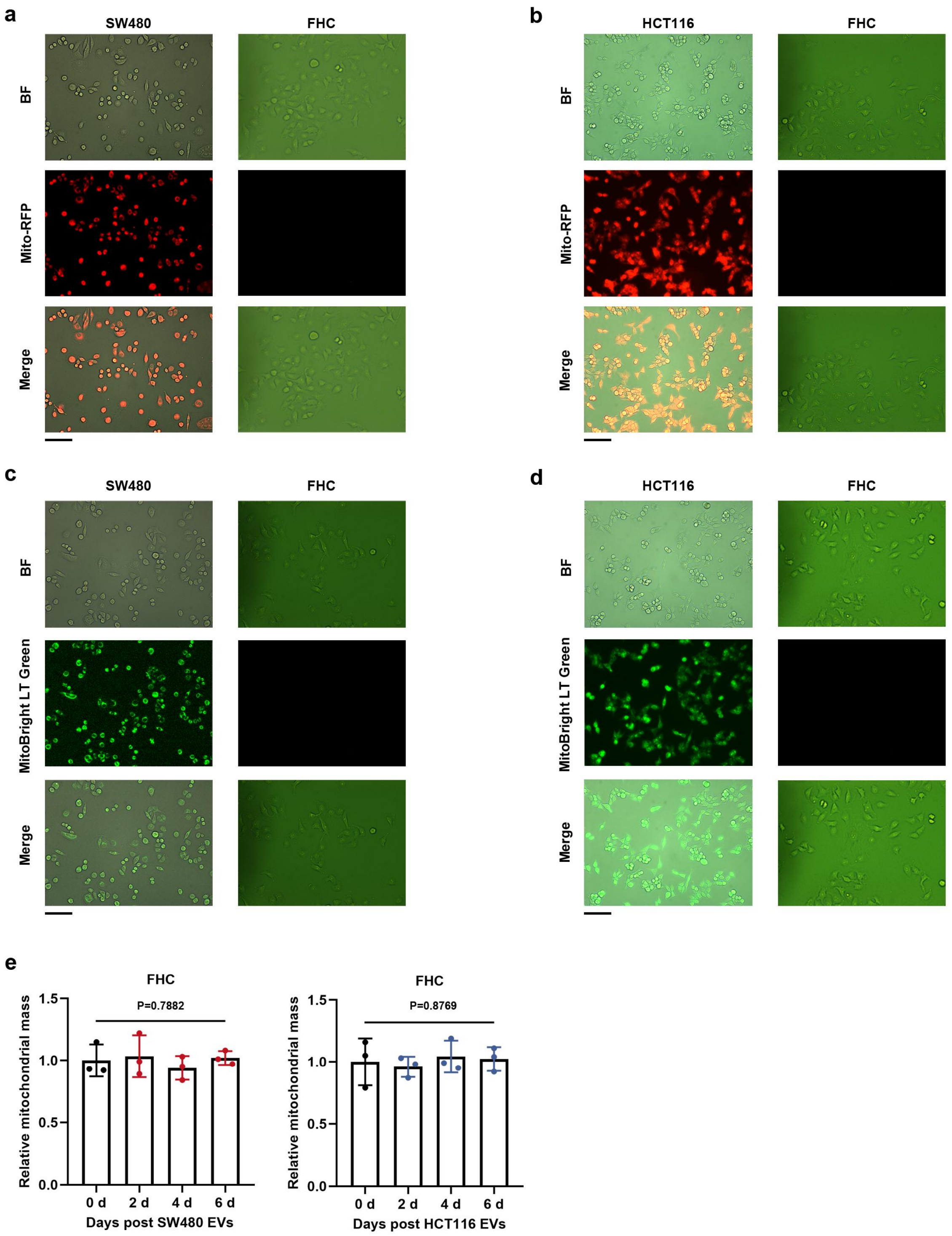

Supplementary Fig. 5. Tumor cells were tested for transport of intact mitochondria to CECs. a, b FHC cells were co-cultured with Mito-RFP-labelled (**a**) SW480 or (**b**) HCT116 cells for four days. Mitochondrial fluorescence was then measured in each cell line. Scale bar, 100 μm. **c, d** FHC cells were co-cultured with MitoBrightLT Green-labelled (**c**) SW480 or (**d**)

HCT116 cells for four days, followed by the detection of mitochondrial fluorescence in each cell line. Scale bar, 100 μm. **e** Determination of mitochondrial mass levels in FHC cells educated with EVs derived from CC cell lines. n = 3 independent experiments. Data are means ± SD. One-way ANOVA with Tukey's multiple comparisons test. Source data are provided as a Source Data file.

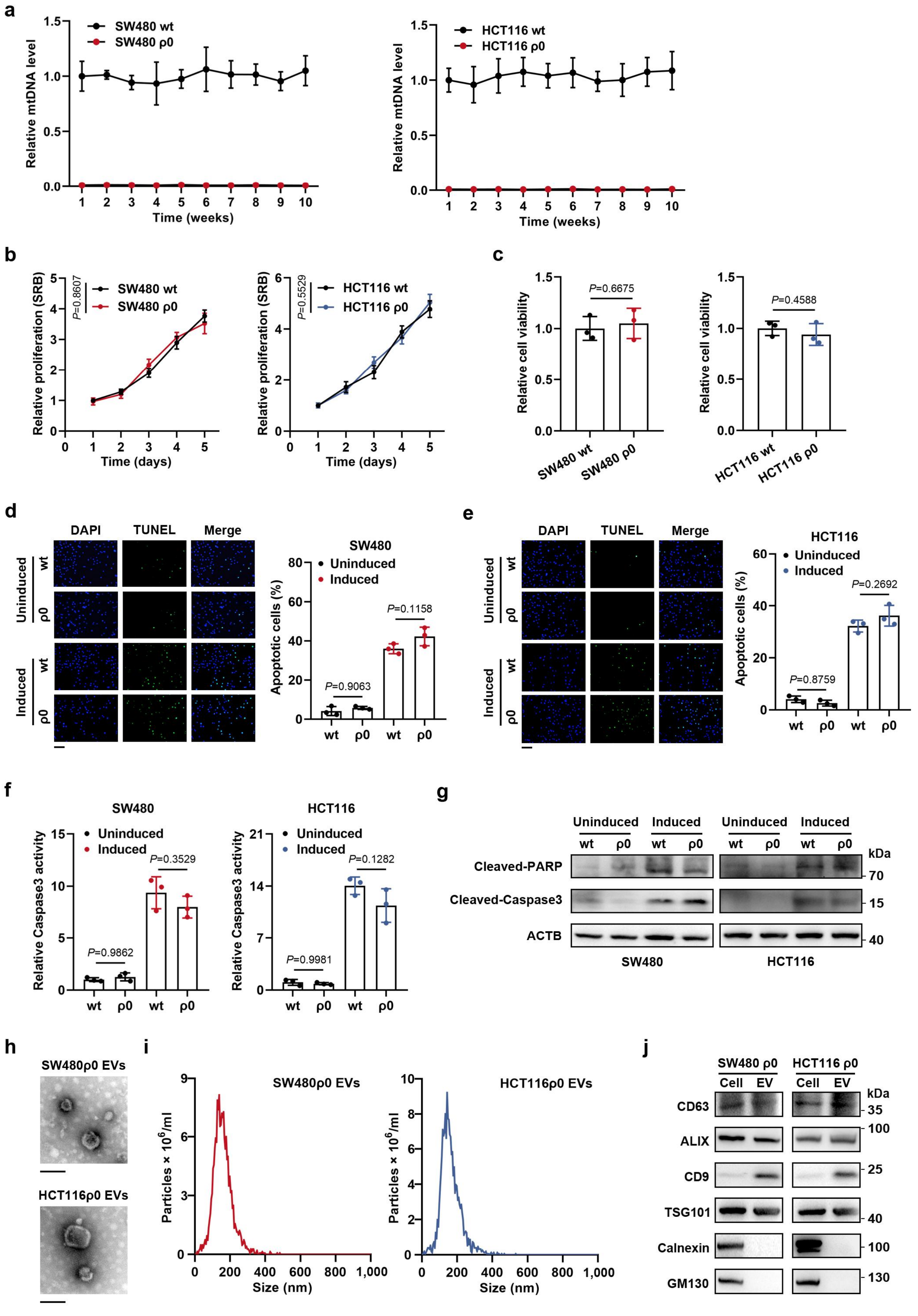
a
SW480 wt
SW480 ρ0
Relative mtDNA level
Time (weeks)
HCT116 wt
HCT116 ρ0
b
Relative proliferation (SRB)
P=0.8607
P=0.5529
Time (days)
c
Relative cell viability
P=0.6675
P=0.4588
SW480 wt
SW480 ρ0
HCT116 wt
HCT116 ρ0
d
DAPI
TUNEL
Merge
Uninduced
Induced
wt
ρ0
SW480
Apoptotic cells (%)
P=0.9063
P=0.1158
e
HCT116
P=0.8759
P=0.2692
f
Relative Caspase3 activity
P=0.9862
P=0.3529
P=0.9981
P=0.1282
g
Cleaved-PARP
Cleaved-Caspase3
ACTB
kDa
70
15
40
h
SW480ρ0 EVs
HCT116ρ0 EVs
i
Particles × 10^6/ml
Size (nm)
j
SW480 ρ0
HCT116 ρ0
Cell
EV
CD63
ALIX
CD9
TSG101
Calnexin
GM130
35
100
25
40
130

Supplementary Fig. 6. Identification of ρ0 cells and their derived EVs. a Detection of the stability of mtDNA content in wild-type or mtDNA-depleted SW480 and HCT116 cells. n = 3 independent experiments. **b, c** Assessment of (**b**) proliferation potential and (**c**) cell viability of wild-type SW480, HCT116, and their ρ0 cells. n = 3 independent experiments. **d-g** The indicated cells were treated with either vehicles or apoptosis inducers. The following measurements were conducted to examine cell apoptosis: (**d, e**) apoptotic cell ratio, (**f**) Caspase3 activity, and (**g**) apoptosis-related protein levels. The samples were derived from the same experiment and processed on the same gel. Scale bar, 100 μm. n = 3 independent experiments. **h-j** Identification of EVs derived from ρ0 cells via (**h**) electron microscopy, (**i**) nanoparticle tracking analysis, and (**j**) immunoblotting. The samples derive from the same experiment but different gels for TSG101, CD9, another for Calnexin, CD63, and another for GM130, ALIX were processed in parallel. Scale bar, 100 nm. Data are means ± SD. Two-sided two-way ANOVA (**b**). Two-tailed t test (**c**). One-way ANOVA with Tukey's multiple comparisons test (**d-f**). Source data are provided as a Source Data file.

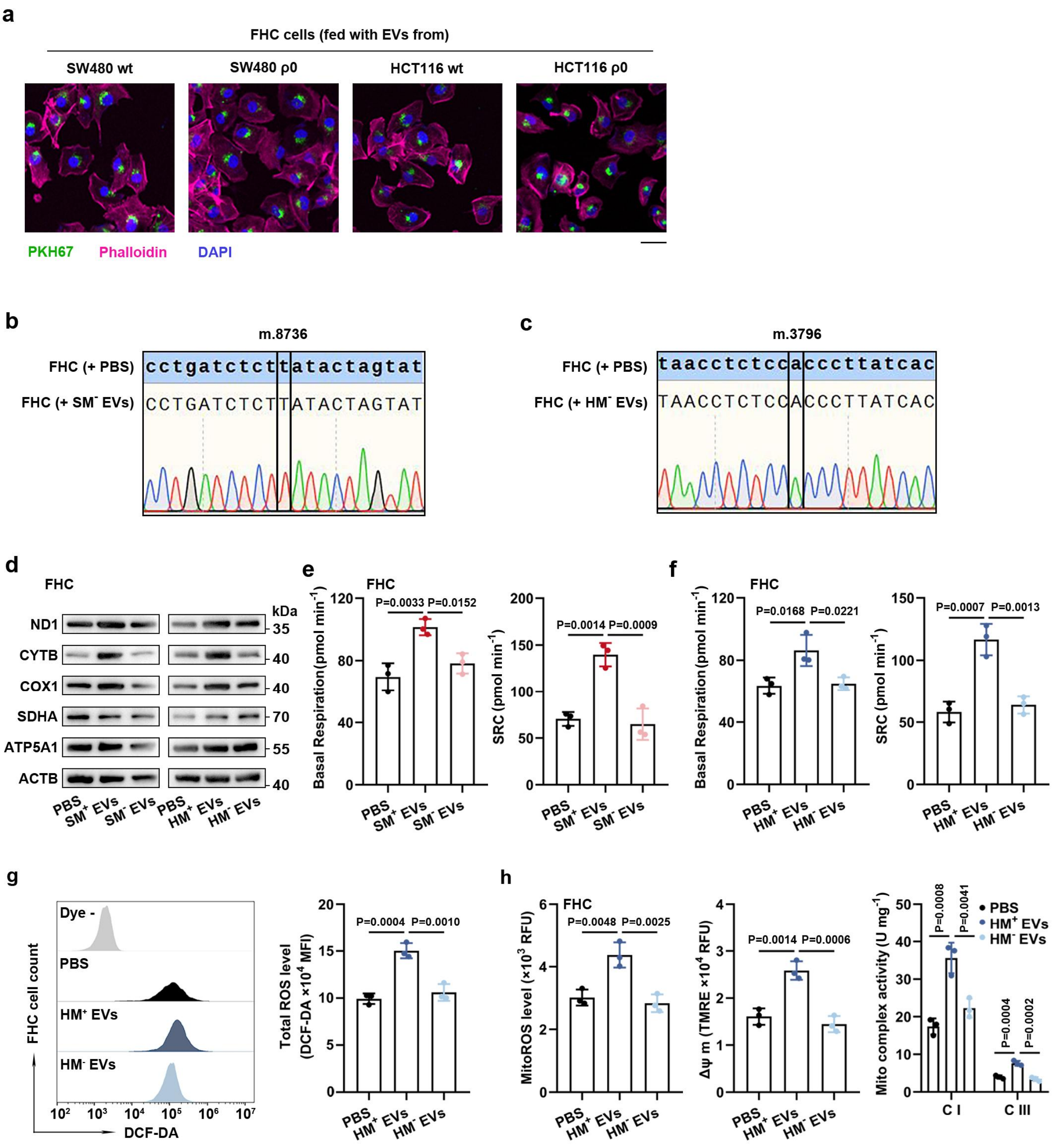

Supplementary Fig. 7. EV-mtDNA transfer results in improved OXPHOS in CECs. a Detection of uptake efficiency of EVs derived from ρ0 or wild-type CC cells by FHC cells. Scale bar, 40 μm. **b, c** Sanger sequencing was performed to detect CC cell-type point mutations in mtDNA purified from FHC cells educated with (**b**) SW480 ρ0- or (**c**) HCT116 ρ0-derived EVs. **d** Changes in expression levels of mtDNA-encoded proteins (ND1, CYTB, and COX1) and nuclear DNA-encoded mitochondrial proteins (SDHA and ATP5A1) in FHC cells educated with mtDNA-sufficient EVs or mtDNA-depleted EVs. The samples derive

from the same experiment but different gels for SDHA, COX1, and ND1, another for ACTB, ATP5A1, and another for CYTB were processed in parallel. **e, f** Changes in basal and spare respiratory capacities of FHC cells. A fraction of FHC cells were educated with (**e**) SM^{+} EVs or SM^{-} EVs, and another subset of FHC cells were educated with (**f**) HM^{+} EVs or HM^{-} EVs. SRC, spare respiratory capacity. n = 3 independent experiments. **g, h** Changes in (**g**) total ROS levels, (**h**) mitochondrial ROS levels, $\Delta\Psi$ m, and activities of mitochondrial complex I and III in FHC cells educated with HM^{+} EVs or HM^{-} EVs for 7 days. n = 3 independent experiments. Data are means ± SD. One-way ANOVA with Tukey's multiple comparisons test. Source data are provided as a Source Data file.

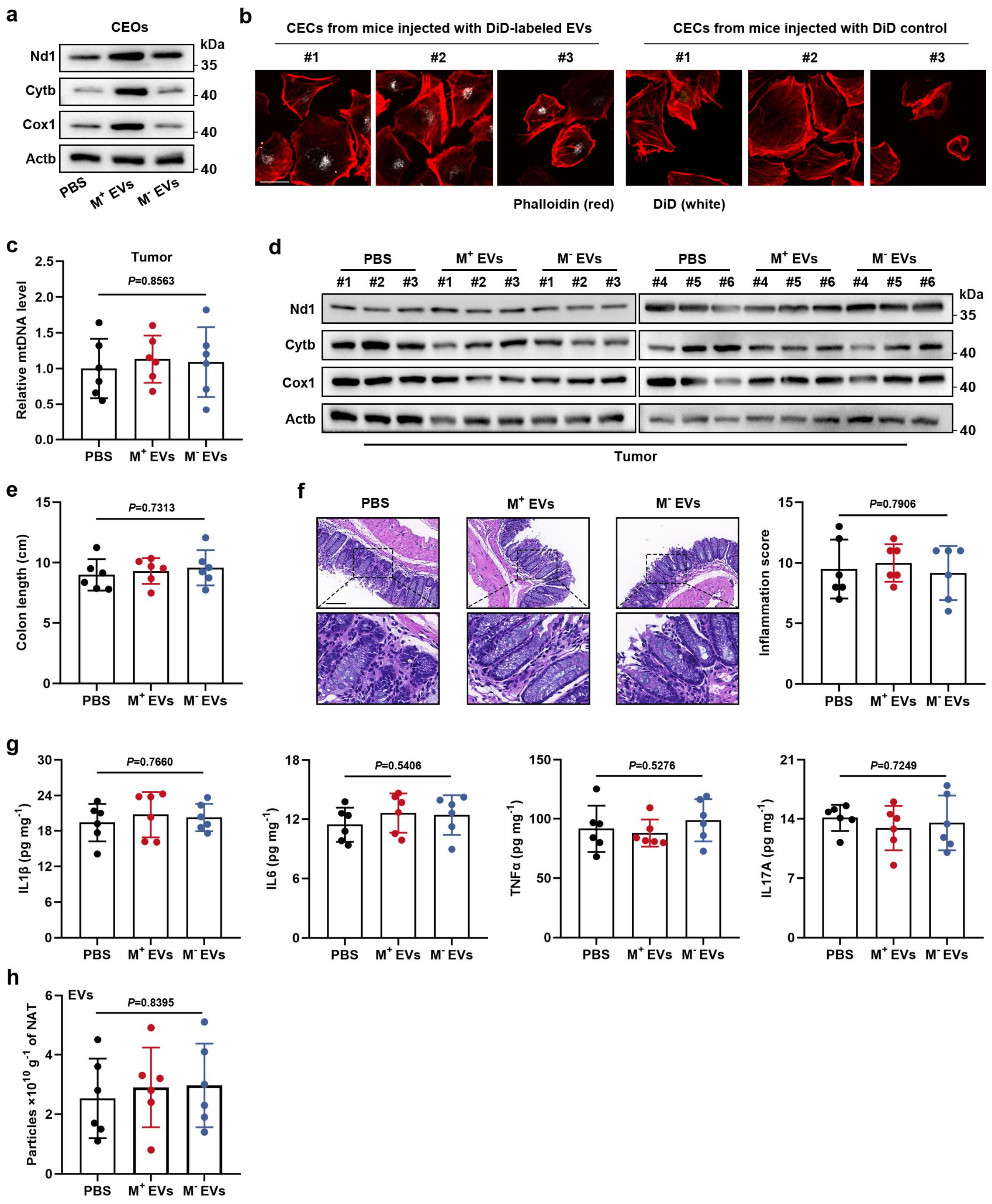

Supplementary Fig. 8. Exogenous EVs exhibit no significant effect on the mtDNA content of tumors and the level of intestinal inflammation in vivo. a Changes in expression levels of mtDNA-encoded proteins in murine colonic epithelial organoids (CEOs) educated with mtDNA-sufficient EVs or mtDNA-depleted EVs. The samples derive from the same experiment but different gels for Cox1, Nd1, another for Actb, and another for Cytb

were processed in parallel. **b** The uptake of MC38 cell-derived DiD-labelled EVs by CECs from murine colon tissues was detected by confocal microscopy. Scale bar, 30 μm. **c, d** qPCR and immunoblotting analysis of (**c**) mtDNA content and (**d**) levels of mtDNA-encoded proteins in tumors from EV-treated murine orthotopic CC model. The samples derive from the same experiment but different gels for Cox1, another for Cytb, and another for Actb, Nd1 were processed in parallel. n = 6 mice per group. **e-g** Inflammation severity of mice treated with mtDNA-sufficient or mtDNA-depleted EVs: (**e**) colon length, (**f**) colonic inflammation score, and (**g**) levels of proinflammatory cytokines in colonic tissue lysates. Scale bar, 100 μm. n = 6 mice per group. **h** EV content in NAT from tumor-bearing mice was detected. n = 6 mice per group. Data are means ± SD. One-way ANOVA with Tukey's multiple comparisons test. Source data are provided as a Source Data file.

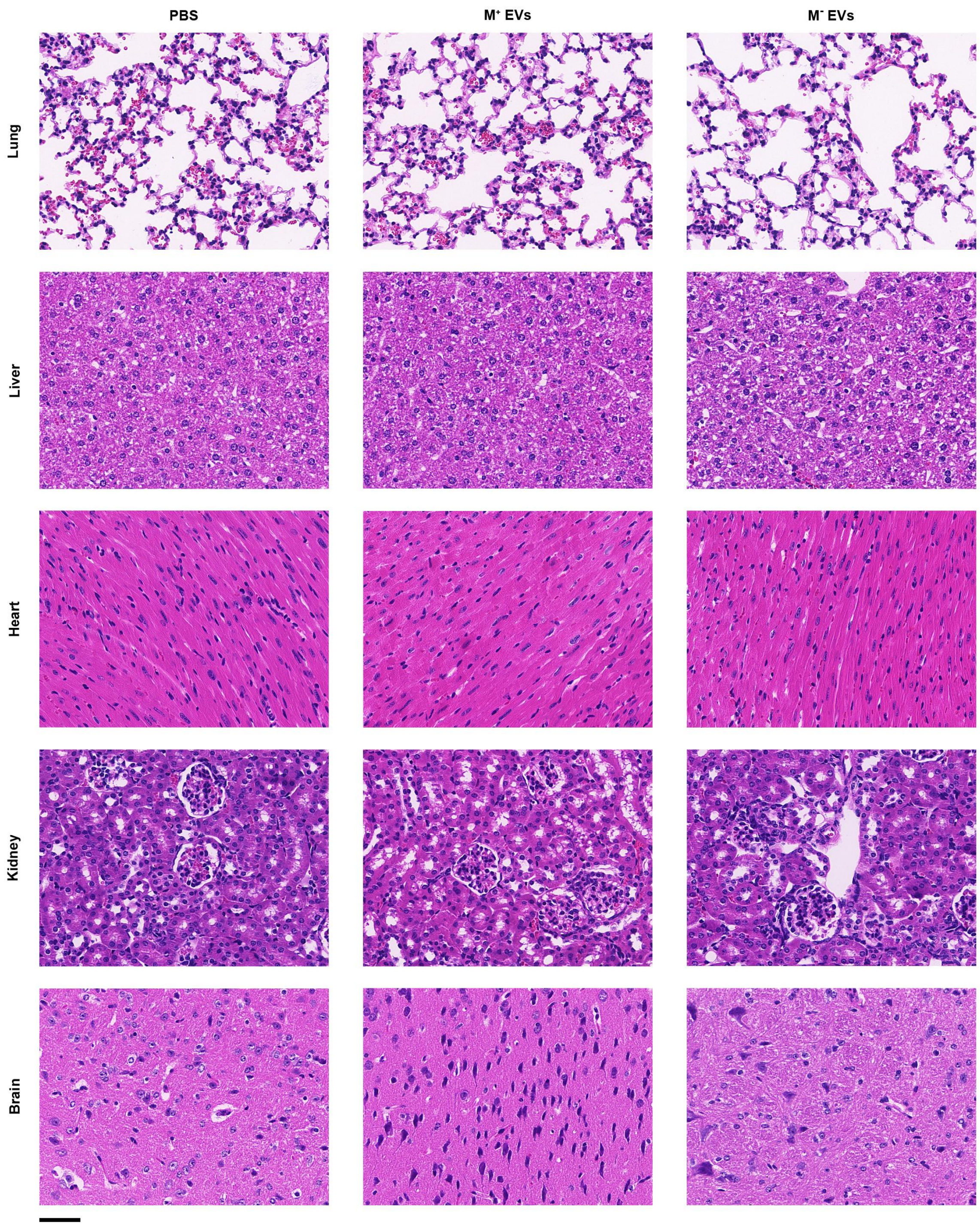

Supplementary Fig. 9. Exogenous CC cell-derived EVs have no significant effect on extra-intestinal organs. HE staining was performed for histopathological analysis of lung, liver, heart, kidney, and brain sampled from mice in each group. Scale bar, 50 μm. Source data are provided as a Source Data file.

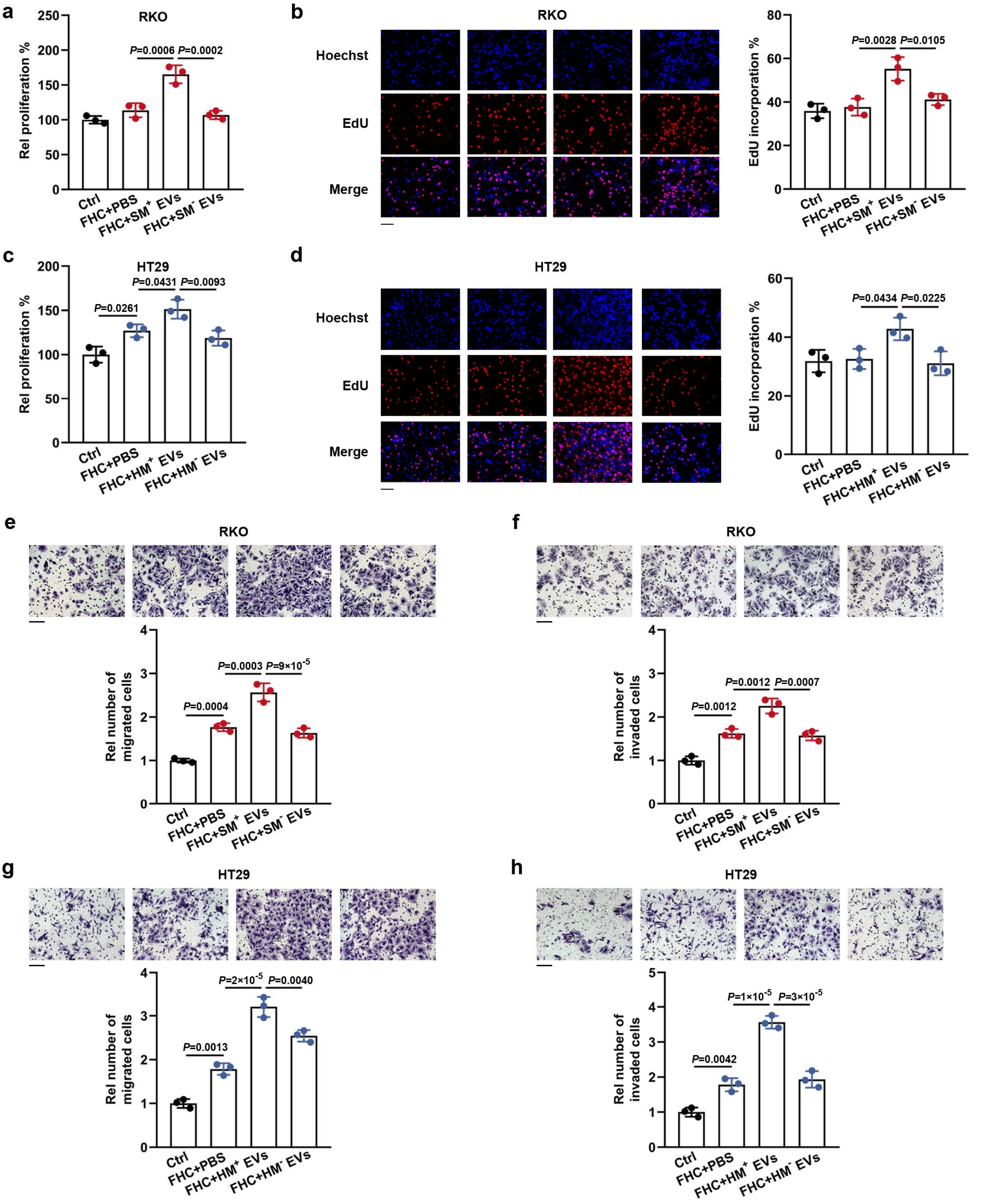

Supplementary Fig. 10. CECs educated with EV-mtDNA promote tumor progression in return. a, b The effect of FHC cells pre-educated with SM^+ EVs or SM^- EVs on the proliferation of RKO cells was detected by (**a**) CCK8 and (**b**) EdU assays. Rel, relative. Scale bar, 100 μm. n = 3 independent experiments. **c, d** The effect of FHC cells pre-educated with HM^+ EVs or HM^- EVs on the proliferation of HT29 cells was assessed by (**c**) CCK8 and (**d**)

EdU assays. Rel, relative. Scale bar, 100 µm. n = 3 independent experiments. **e, f** The roles of FHC cells pre-educated with SM^+ EVs or SM^- EVs in (**e**) migration and (**f**) invasion of RKO cells were determined by Transwell assays. Rel, relative. Scale bar, 100 µm. n = 3 independent experiments. **g, h** The roles of FHC cells pre-educated with HM^+ EVs or HM^- EVs in (**g**) migration and (**h**) invasion of HT29 cells were also examined by Transwell assays. Rel, relative. Scale bar, 100 µm. n = 3 independent experiments. Data are means ± SD. One-way ANOVA with Tukey's multiple comparisons test. Source data are provided as a Source Data file.

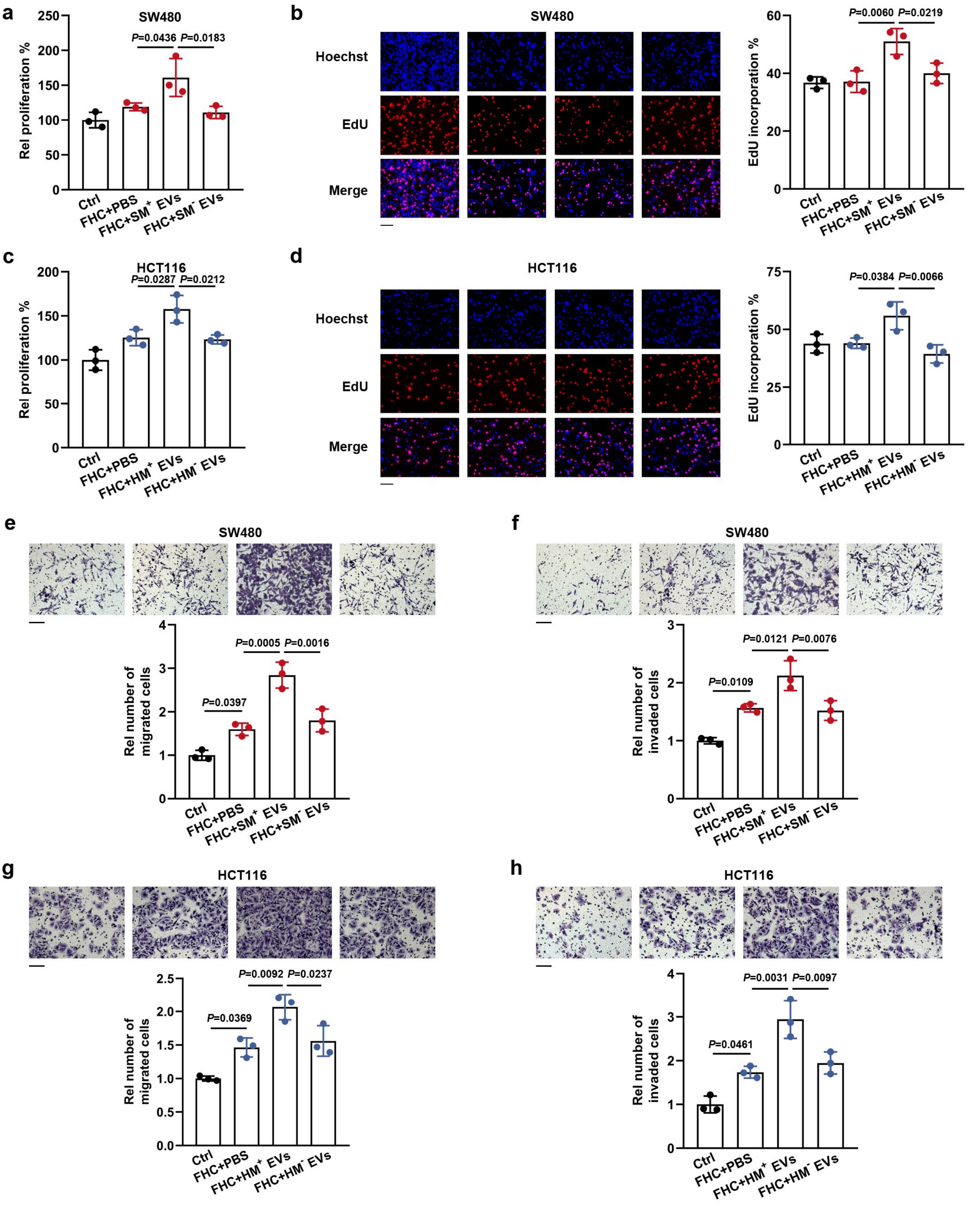

Supplementary Fig. 11. EV-mtDNA-educated CECs accelerate tumor progression. a, b The effect of FHC cells pre-educated with SM^+ EVs or SM^- EVs on the proliferation of SW480 cells was detected by (**a**) CCK8 and (**b**) EdU assays. Rel, relative. Scale bar, 100 μm. n = 3 independent experiments. **c, d** The effect of FHC cells pre-educated with HM^+ EVs or HM^- EVs on the proliferation of HCT116 cells was assessed by (**c**) CCK8 and (**d**) EdU assays.

Rel, relative. Scale bar, 100 μm. n = 3 independent experiments. **e, f** The roles of FHC cells pre-educated with SM^{+} EVs or SM^{-} EVs in (**e**) migration and (**f**) invasion of SW480 cells were determined by Transwell assays. Rel, relative. Scale bar, 100 μm. n = 3 independent experiments. **g, h** The roles of FHC cells pre-educated with HM^{+} EVs or HM^{-} EVs in (**g**) migration and (**h**) invasion of HCT116 cells were also examined by Transwell assays. Rel, relative. Scale bar, 100 μm. n = 3 independent experiments. Data are means ± SD. One-way ANOVA with Tukey's multiple comparisons test. Source data are provided as a Source Data file.

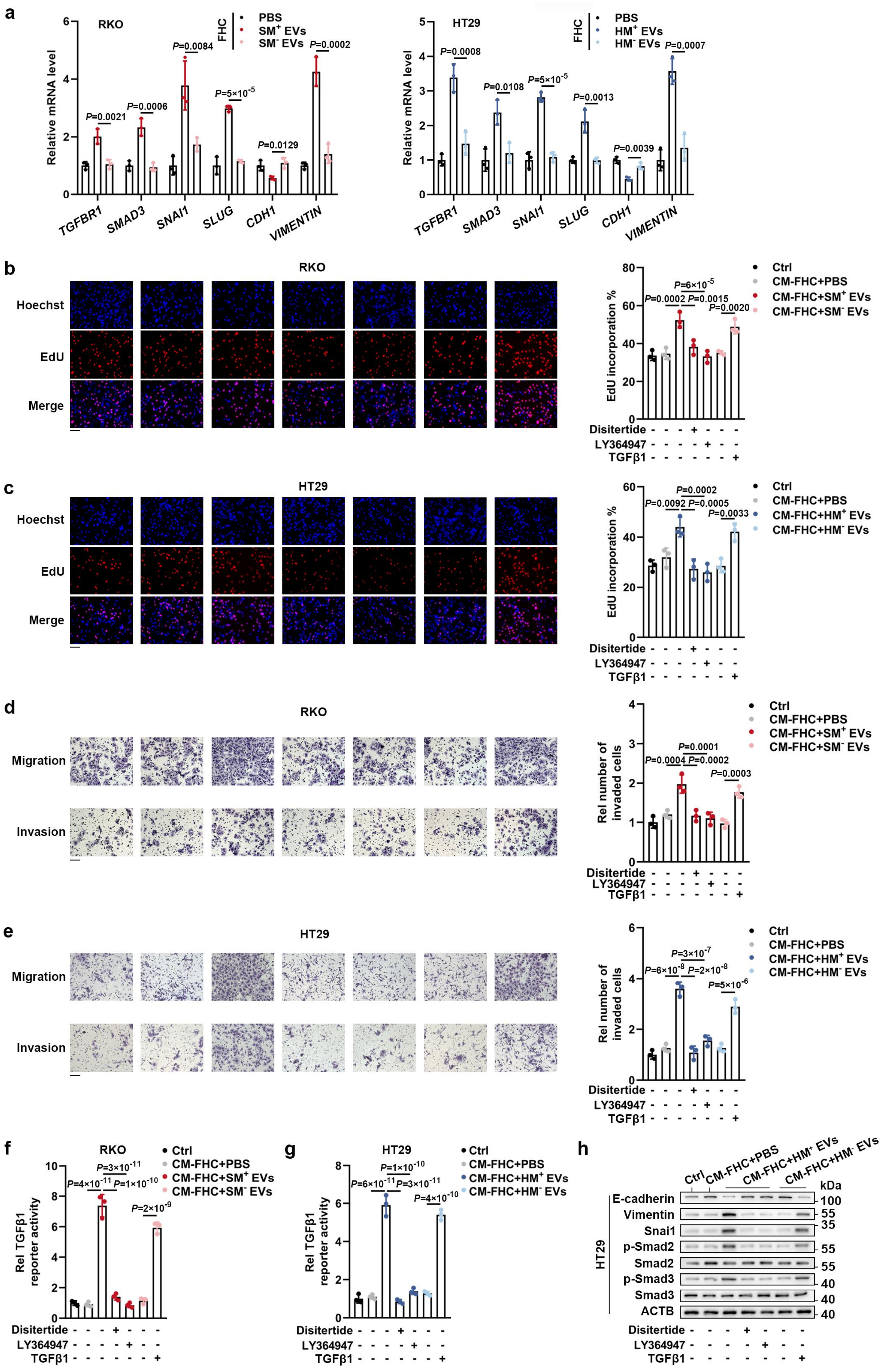
a
RKO
HT29
FHC
PBS
SM+ EVs
SM- EVs
HM+ EVs
HM- EVs
Relative mRNA level
P=0.0021
P=0.0006
P=0.0084
P=5×10-5
P=0.0129
P=0.0002
P=0.0008
P=0.0108
P=5×10-5
P=0.0013
P=0.0039
P=0.0007
TGFBR1
SMAD3
SNAI1
SLUG
CDH1
VIMENTIN
b
c
Hoechst
EdU
Merge
Ctrl
CM-FHC+PBS
CM-FHC+SM+ EVs
CM-FHC+SM- EVs
CM-FHC+HM+ EVs
CM-FHC+HM- EVs
EdU incorporation %
P=6×10-5
P=0.0002
P=0.0015
P=0.0020
P=0.0002
P=0.0092
P=0.0005
P=0.0033
Disitertide
LY364947
TGFβ1
d
e
Migration
Invasion
Rel number of invaded cells
P=0.0001
P=0.0004
P=0.0002
P=0.0003
P=3×10-7
P=6×10-8
P=2×10-8
P=5×10-6
f
g
h
Rel TGFβ1 reporter activity
P=3×10-11
P=4×10-11
P=1×10-10
P=2×10-9
P=1×10-10
P=6×10-11
P=3×10-11
P=4×10-10
E-cadherin
Vimentin
Snai1
p-Smad2
Smad2
p-Smad3
Smad3
ACTB
kDa
100
55
35
55
55
40
40
40

Supplementary Fig. 12. TGFβ1 upregulation in CECs increases tumor malignancy. a RKO and HT29 cells were co-cultured with FHC cells pre-educated with mtDNA-sufficient or mtDNA-depleted EVs. The expression levels of TGFβ1-related genes in tumor cells were then determined by qPCR analysis. n = 3 independent experiments. **b, c** (**b**) RKO and (**c**) HT29 cells were incubated with CM from FHC cells pre-treated with mtDNA-rich or mtDNA-depleted EVs, for 48 h. Meanwhile, a subset of RKO and HT29 cells were treated with disitertide (10 μM), LY364947 (1 μM), or TGFβ1 (1 ng mL^{-1}). Then, the proliferative abilities of these cells were detected by EdU assays. Scale bar, 100 μm. n = 3 independent experiments. **d, e** The migration and invasion abilities of (**d**) RKO and (**e**) HT29 were evaluated by Transwell assays. RKO and HT29 cells were treated as described in (**b, c**). Rel, relative. Scale bar, 100 μm. n = 3 independent experiments. **f, g** TGFβ1 reporter plasmids were transfected into CC cells. Then, the luciferase activities in (**f**) RKO and (**g**) HT29 cells were monitored. CC cells were treated as described in (**b, c**). Rel, relative. n = 3 independent experiments. **h** The levels of phosphorylated Smad2/3 and EMT markers (E-cadherin, Vimentin, Snai1) in HT29 cells were detected by immunoblotting. The samples derive from the same experiment but different gels for E-cadherin, Vimentin, and Snai1, another for ACTB, p-Smad2, another for Smad2, p-Smad3, and another for Smad3 were processed in parallel. HT29 cells were treated as described in (**c**). Data are means ± SD. One-way ANOVA with Tukey's multiple comparisons test. Source data are provided as a Source Data file.

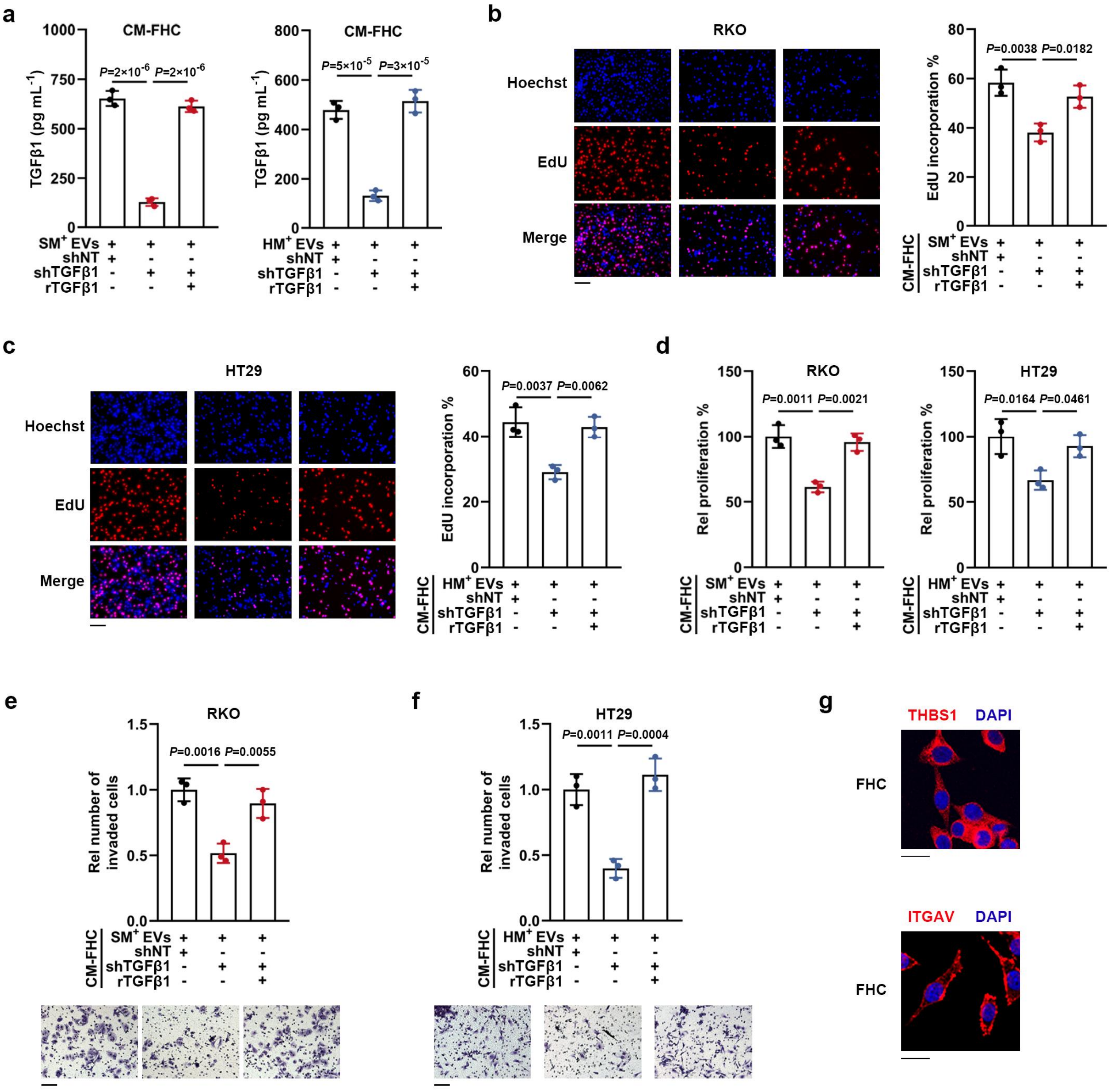

Supplementary Fig. 13. TGFβ1 increase caused by EV-mtDNA transfer into CECs is responsible for the enhanced malignant phenotype in CC cells. a FHC cells expressing shNT or shTGFβ1 were infected with the lentivirus expressing rTGFβ1, followed by education with SM$^+$ EVs (left) or HM$^+$ EVs (right). ELISA assays were performed to detect TGFβ1 levels in CM from FHC cells. n = 3 independent experiments. **b, c** The CM from FHC cells treated as described in (**a**) was used to incubate (**b**) RKO and (**c**) HT29 for 48 h. EdU assays were performed. Scale bar, 100 μm. n = 3 independent experiments. **d** The proliferative abilities of RKO and HT29 cells were also detected by CCK8 assays. Rel, relative. n = 3 independent experiments. **e, f** Transwell invasion assays were performed to

determine the invasion abilities of (**e**) RKO and (**f**) HT29. The CC cells were treated as described in (**b, c**). Rel, relative. Scale bar, 100 μm. n = 3 independent experiments. **g** Immunofluorescence assays were performed to observe the expression and localization of THBS1 and ITGAV in FHC cells. Scale bar, 20 μm. Data are means ± SD. One-way ANOVA with Tukey's multiple comparisons test. Source data are provided as a Source Data file.

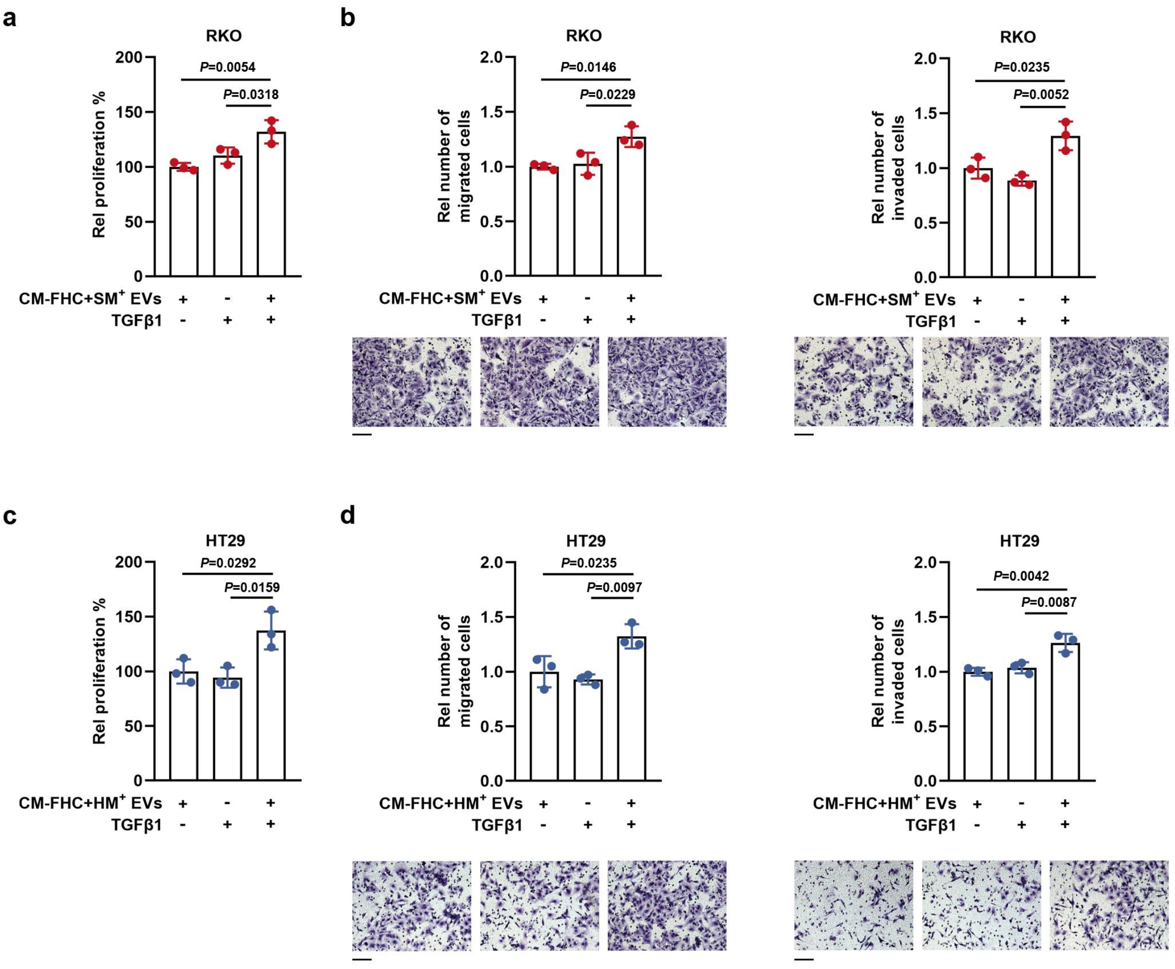

Supplementary Fig. 14. Exogenous TGFβ1 and EV-mtDNA-educated CECs synergistically promote tumor progression. a, b The effect of exogenous TGFβ1 and EV-mtDNA-educated CECs on proliferation, migration, and invasion of RKO cells was detected by (**a**) CCK8 and (**b**) Transwell assays. Rel, relative. Scale bar, 100 µm. n = 3 independent experiments. **c, d** The effect of exogenous TGFβ1 and EV-mtDNA-educated CECs on proliferation, migration, and invasion of HT29 cells was measured by (**c**) CCK8 and (**d**) Transwell assays. Rel, relative. Scale bar, 100 µm. n = 3 independent experiments. Data are means ± SD. One-way ANOVA with Tukey's multiple comparisons test. Source data are provided as a Source Data file.

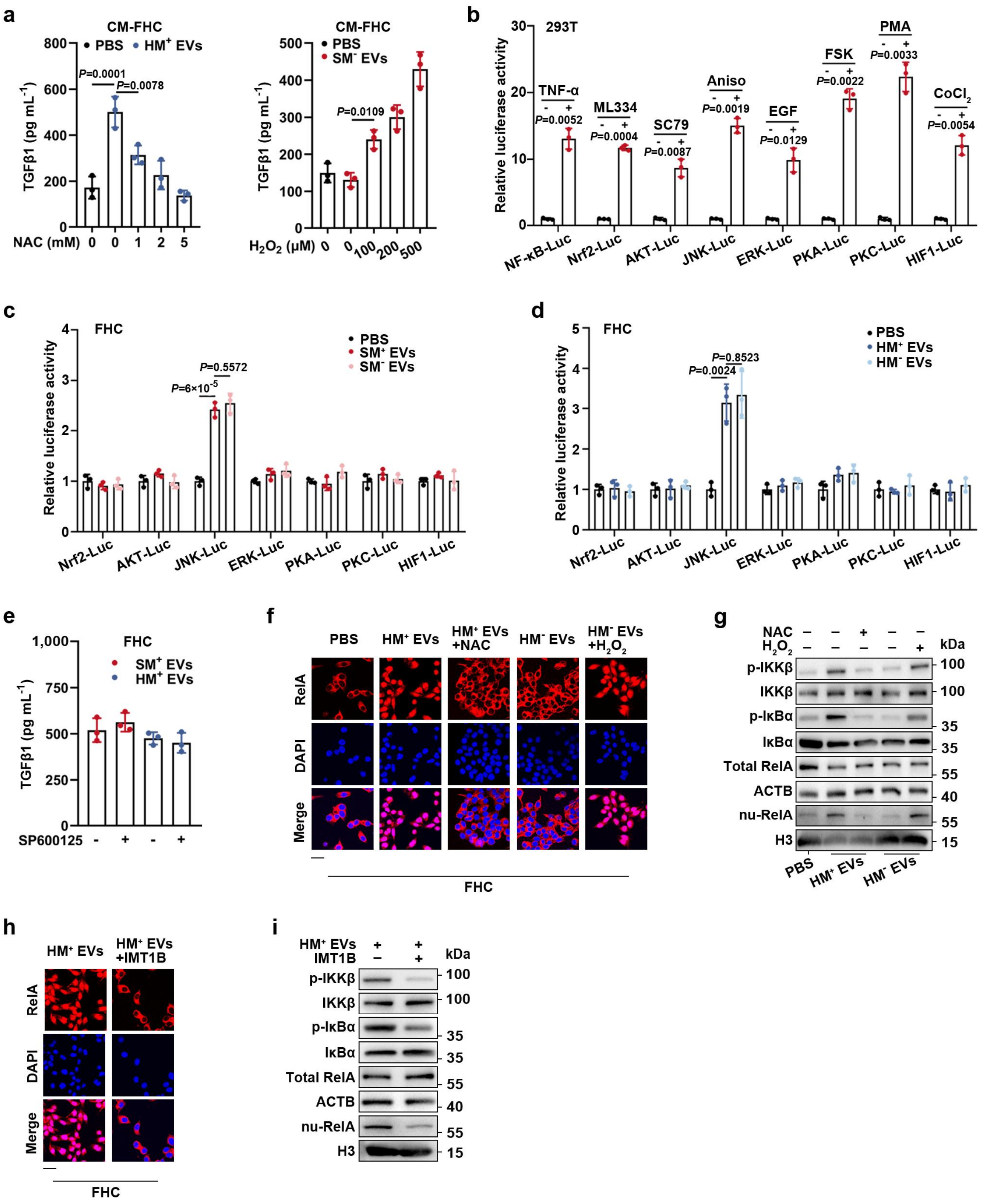

Supplementary Fig. 15. Increased ROS induced by EV-mtDNA upregulates TGFβ1 expression and activates NF-κB pathway. a ELISA assays were performed to determine TGFβ1 levels in CM from HM$^+$ EV-educated FHC cells with or without NAC treatment (left). TGFβ1 levels in CM from SM$^-$ EV-educated FHC cells in the presence or absence of H_2O_2 stimulation were also detected by ELISA (right). n = 3 independent experiments. **b** The responsiveness of indicated luciferase reporters were verified in 293T cells, which were

stimulated with TNF-α (30 min, 10 ng mL^{-1}), ML334 (12 h, 100 μM), SC79 (24 h, 10 μM), Anisomycin (24 h, 10 μM), EGF (24 h, 50 ng mL^{-1}), Forskolin (6 h, 20 μM), PMA (12 h, 30 nM), or $CoCl_2$ (8 h, 150 μM). n = 3 independent experiments. **c, d** The luciferase activities were determined in FHC cells educated with EVs derived from (**c**) wild-type/ρ0 SW480, or (**d**) wild-type/ρ0 HCT116. n = 3 independent experiments. **e** Detection of TGFβ1 levels in CM from EV-educated FHC cells in the presence or absence of SP600125 treatment (10 μM, 24 h). n = 3 independent experiments. **f, g** A subset of FHC cells educated with HM^+ EVs were treated with NAC (5 mM), and a portion of FHC cells educated with HM^- EVs were stimulated with H_2O_2 (500 μM). The levels of phosphorylated IKKβ and IκBα, and nuclear translocation of RelA in FHC cells were detected by (**f**) immunofluorescence and (**g**) immunoblotting. Scale bar, 30 μm. **h, i** The levels of phosphorylated IKKβ and IκBα, and nuclear translocation of RelA in FHC cells were determined by (**h**) immunofluorescence and (**i**) immunoblotting. FHC cells were educated with HM^+ EVs, followed by stimulation with or without IMT1B (1 μM). Scale bar, 30 μm. As shown in (**g, i**), the total protein samples derive from the same experiment but different gels for IKKβ, p-IκBα, and another for p-IKKβ, total RelA, ACTB, and IκBα were processed in parallel. The nuclear protein samples for detecting nu-RelA and H3 were processed on the same gel. Data are means ± SD. Two-tailed t test (**b, e**). One-way ANOVA with Tukey's multiple comparisons test (**a, c,** and **d**). Source data are provided as a Source Data file.

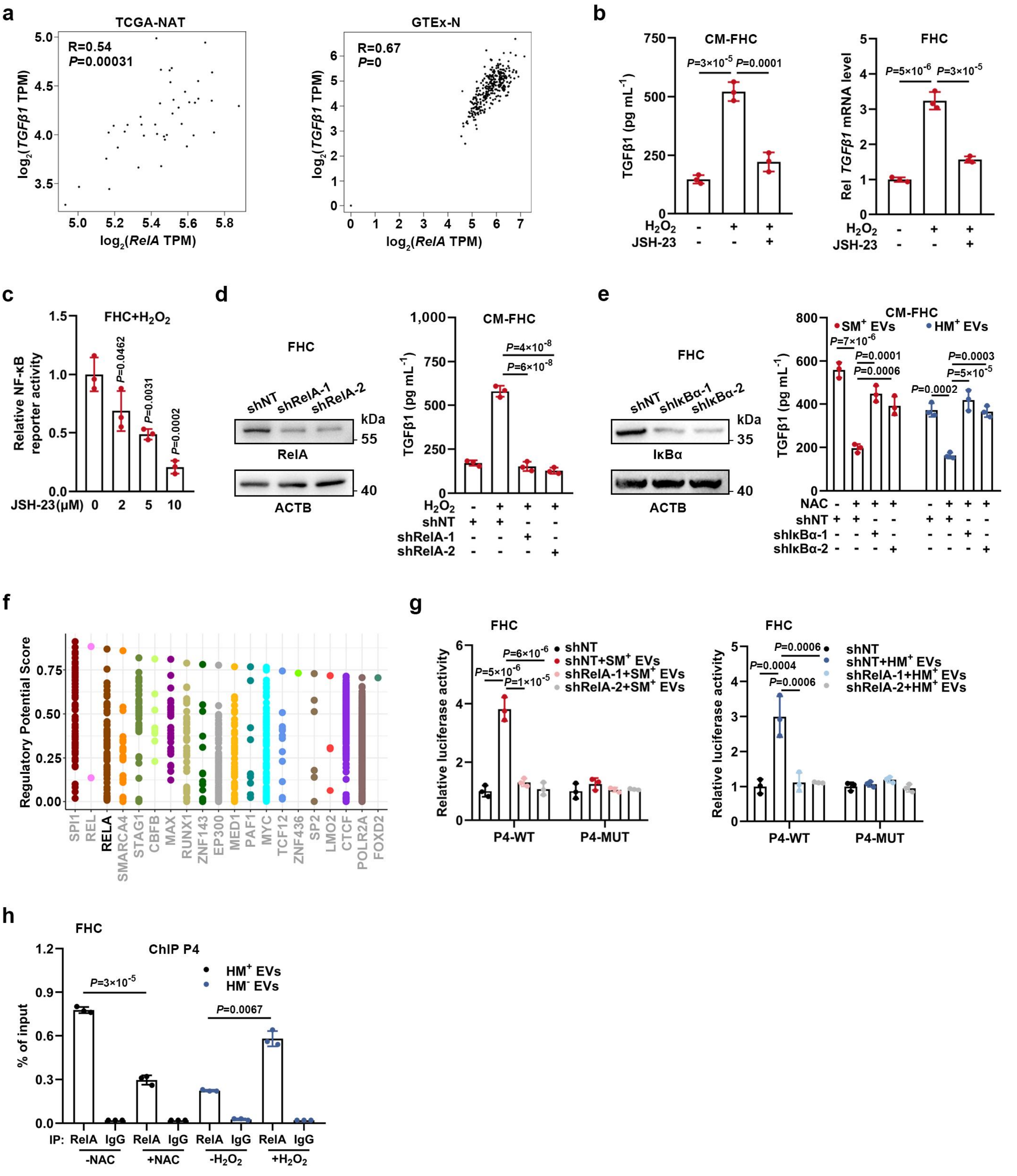

Supplementary Fig. 16. Nuclear translocation of RelA driven by ROS accelerates transcription of *TGFβ1* via interaction with *TGFβ1* promoter. a TCGA and GTEx RNA-sequencing data were analyzed to describe the correlation between *RelA* and *TGFβ1* in NAT or completely normal colonic mucosa using Pearson correlation analysis. **b** The effect of the NF-κB pathway on TGFβ1 expression at both protein and mRNA levels was evaluated in H_2O_2-stimulated FHC cells in the presence or absence of 10-μM JSH-23 treatment. Rel,

relative. n = 3 independent experiments. **c** The inhibitory effect of JSH-23 on the NF-κB pathway was verified. n = 3 independent experiments. **d** FHC cells expressing shNT or shRelA were stimulated with H_2O_2. TGFβ1 protein levels were then detected. The samples were derived from the same experiment and processed on the same gel. n = 3 biological replicates. **e** EV-educated FHC cells expressing shNT or shIκBα were treated with NAC. Subsequently, TGFβ1 protein levels were determined. The samples were derived from the same experiment and processed on the same gel. n = 3 biological replicates. **f** ChIP-seq data from public databases were analyzed to reveal the potential interaction between RelA and *TGFβ1* promoter. **g** Luciferase reporter plasmids containing the wild-type or mutant RelA binding site P4 were transfected into FHC cells stably expressing shNT or shRelA. Then, the luciferase activities were detected after indicated EV treatment. n = 3 biological replicates. **h** ChIP assays were performed to examine RelA enrichment on P4 in NAC- or H_2O_2-treated FHC cells educated with indicated EVs. n = 3 independent experiments. Data are means ± SD. Two-tailed t test (**h**). One-way ANOVA with Tukey's multiple comparisons test (**b-e, g**). Source data are provided as a Source Data file.

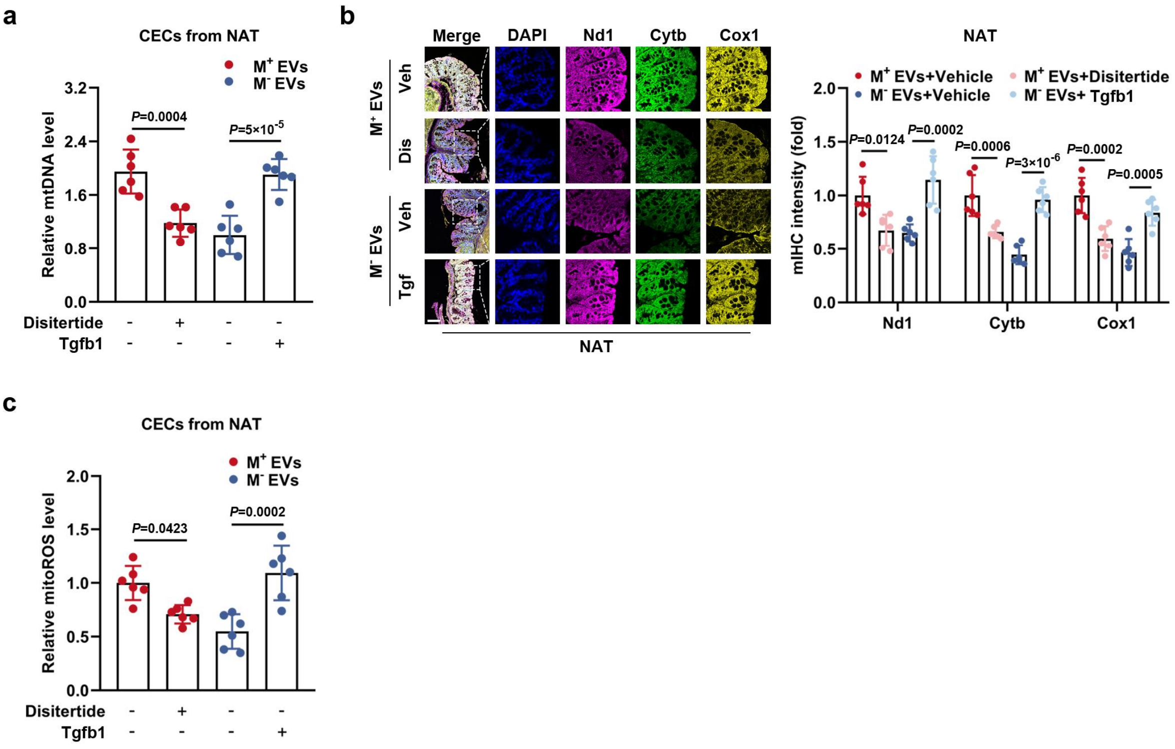

Supplementary Fig. 17. TGFβ1-dependent tumor progression enhanced OXPHOS phenotype in adjacent CECs. a The mtDNA content in CECs isolated from NAT of murine orthotopic CC model receiving indicated treatment were detected. n = 6 mice per group. **b** Representative images (left) of mIHC staining for DAPI (blue), Nd1 (magenta), Cytb (green), and Cox1 (yellow) in sections from NAT of murine orthotopic CC model receiving indicated treatment. Scale bar, 100 μm. The co-expression levels of these mitochondrial proteins were then determined by the quantification of fluorescent intensity (right). Veh, Vehicle; Dis, Disitertide; Tgf, Tgfb1. n = 6 mice per group. **c** Detection of the mitochondrial ROS levels in CECs isolated from NAT of murine orthotopic CC model receiving indicated treatment. n = 6 mice per group. Data are means ± SD. One-way ANOVA with Tukey's multiple comparisons test. Source data are provided as a Source Data file.

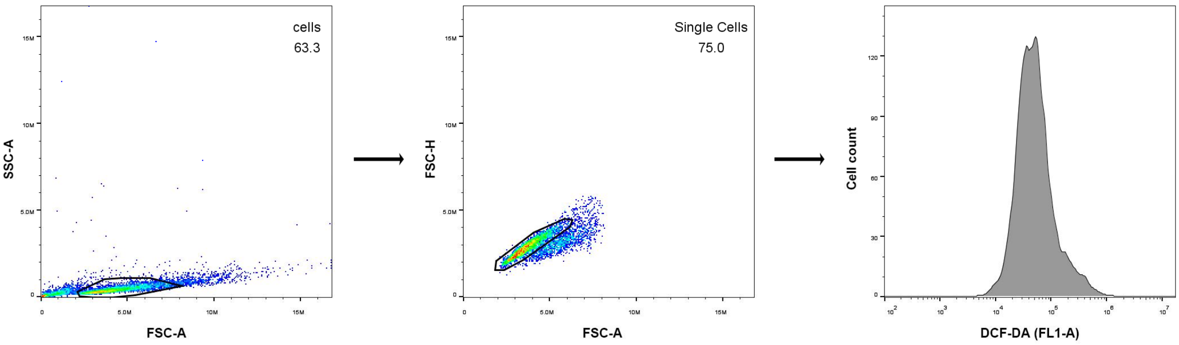

This figure exemplifies the gating strategy used for Flow cytometry analysis.

Supplementary Table 1 Relationships between serum EV-mtDNA level and clinical factors in 82 colon cancer patients

Characteristics	No. of patients (%)	Serum EV-mtDNA level		*P*
		Low n = 41 (%)	High n = 41 (%)	
**Age**				
<65	26 (31.7)	14 (17.1)	12 (14.6)	0.635
≥65	56 (68.3)	27 (32.9)	29 (35.4)	
**Gender**				
Female	32 (39.0)	17 (20.7)	15 (18.3)	0.651
Male	50 (61.0)	24 (29.3)	26 (31.7)	
**Location**				
Left	45 (54.9)	22 (26.8)	23 (28.0)	0.824
Right	37 (45.1)	19 (23.2)	18 (22.0)	
**pT stage**				
T1	6 (7.3)	5 (6.1)	1 (1.2)	0.010
T2	9 (11.0)	8 (9.8)	1 (1.2)	
T3	53 (64.6)	21 (25.6)	32 (39.0)	
T4	14 (17.1)	7 (8.5)	7 (8.5)	
**Lymph node metastasis**				
N0	40 (48.8)	25 (30.5)	15 (18.3)	0.047
N1	27 (32.9)	12 (14.6)	15 (18.3)	
N2	15 (18.3)	4 (4.9)	11 (13.4)	
**Distant metastasis**				
M0	62 (75.6)	36 (43.9)	26 (31.7)	0.010
M1	20 (24.4)	5 (6.1)	15 (18.3)	
**AJCC stage**				
I	13 (15.9)	12 (14.6)	1 (1.2)	0.002
II	24 (29.3)	13 (15.9)	11 (13.4)	
III	25 (30.5)	11 (13.4)	14 (17.1)	
IV	20 (24.4)	5 (6.1)	15 (18.3)	
**Differentiation**				
Well-moderate	67 (81.7)	33 (40.2)	34 (41.5)	0.775
Poor	15 (18.3)	8 (9.8)	7 (8.5)	

The two-sided Chi-square test was used.

Supplementary Table 2 Details of the antibodies used in this study

Primary antibody	Concentration	Specificity	Company
CD63	1:500 (IB)	Rabbit polyclonal	Proteintech (25682-1-AP)
CD9	1:2,000 (IB)	Rabbit polyclonal	Proteintech (20597-1-AP)
TSG101	1:10,000 (IB)	Rabbit polyclonal	Proteintech (28283-1-AP)
Calnexin	1:5,000 (IB)	Rabbit polyclonal	Proteintech (10427-2-AP)
ALIX	1:20,000 (IB)	Rabbit polyclonal	Proteintech (12422-1-AP)
GM130	1:10,000 (IB)	Rabbit polyclonal	Proteintech (11308-1-AP)
ACTB	1:5,000 (IB)	Rabbit polyclonal	Proteintech (20536-1-AP)
H3	1:10,000 (IB)	Rabbit polyclonal	Proteintech (17168-1-AP)
TFAM	1:20,000 (IB)	Rabbit polyclonal	Proteintech (22586-1-AP)
RelA	1:1,000 (IB);1:100 (ChIP); 1:800 (IF)	Rabbit monoclonal (D14E12)	Cell Signaling Technology (#8242)
THBS1	1:100 (IF)	Rabbit monoclonal (EPR22927-54)	Abcam (ab267388)
ITGAV	1:500 (IF)	Rabbit monoclonal (EPR16800)	Abcam (ab179475)
IKKβ	1:1,000 (IB)	Rabbit monoclonal (D30C6)	Cell Signaling Technology (#8943)
p-IKKβ	1:500 (IB)	Rabbit polyclonal	ABclonal (AP1237)
IκBα	1:1,000 (IB)	Rabbit monoclonal (44D4)	Cell Signaling Technology (#4812)
p-IκBα	1:1,000 (IB)	Rabbit monoclonal (14D4)	Cell Signaling Technology (#2859)
ND1	1:1,000 (IB);1:1,000 (mIHC)	Rabbit polyclonal	ABclonal (A17967)
CYTB	1:1,000 (IB);1:1,000 (mIHC)	Rabbit polyclonal	ABclonal (A17966)
COX1	1:500 (IB);1:2,000 (mIHC)	Rabbit polyclonal	ABclonal (A17889)
ATP5A1	1:5,000 (IB)	Rabbit polyclonal	Proteintech (14676-1-AP)
SDHA	1:5,000 (IB)	Rabbit polyclonal	Proteintech (14865-1-AP)
Smad2	1:1,000 (IB)	Rabbit monoclonal (D43B4)	Cell Signaling Technology (#5339)
p-Smad2	1:1,000 (IB)	Rabbit monoclonal (E8F3R)	Cell Signaling Technology (#18338)
Smad3	1:1,000 (IB)	Rabbit monoclonal (C67H9)	Cell Signaling Technology (#9523)
p-Smad3	1:1,000 (IB)	Rabbit monoclonal (C25A9)	Cell Signaling Technology (#9520)
E-cadherin	1:1,000 (IB);1:2,000 (mIHC)	Rabbit monoclonal (24E10)	Cell Signaling Technology (#3195)
Vimentin	1:1,000 (IB);1:1,000 (mIHC)	Rabbit polyclonal	ABclonal (A2584)
Snai1	1:1,000 (IB);1:500 (mIHC)	Rabbit polyclonal	ABclonal (A5243)
TIM23	1:2,000 (IB)	Rabbit polyclonal	Proteintech (11123-1-AP)
HNRNPU	1:5,000 (IB)	Rabbit polyclonal	Proteintech (14599-1-AP)
Cleaved-PARP	1:1,000 (IB)	Rabbit monoclonal (D64E10)	Cell Signaling Technology (#5625)
Cleaved-Caspase3	1:1,000 (IB)	Rabbit monoclonal (5A1E)	Cell Signaling Technology (#9664)
Calreticulin	1:1,000 (IB)	Rabbit polyclonal	Proteintech (10292-1-AP)
F4/80	1:20,000 (mIHC)	Rabbit polyclonal	Proteintech (29414-1-AP)
Ly6G	1:5,000 (mIHC)	Rabbit monoclonal (EPR22909-135)	Abcam (ab238132)
Epcam	1:1,000 (IF)	Rabbit polyclonal	Proteintech (21050-1-AP)
**Secondary antibody**	**Concentration**	**Specificity**	**Company**
CY3-conjugated	1:200 (IF)	goat anti-rabbit IgG	Affinity Biosciences (#S0011)
HRP-conjugated	1:5,000 (IB)	goat anti-rabbit IgG	Affinity Biosciences (#S0001)
HRP-conjugated	ready-to-use (mIHC)	goat anti-rabbit IgG	Panovue (10079100050)

IB: immunoblotting; mIHC: multiplex immunohistochemistry; IF: immunofluorescence
ChIP: chromatin immunoprecipitation

Supplementary Table 3 Primers for qPCR

Gene	Sequence	
TGFβ1 (cDNA)	F 5'CTCGCCCTGTACAACAGCAC3'	R 5'GGGTTTCCACCATTAGCAC3'
ACTB (cDNA)	F 5'ACAGAGCCTCGCCTTTGCC3'	R 5'TGGGGTACTTCAGGGTGAGG3'
ND1 (mtDNA)	F 5'ATATACAACTACGCAAAGGCCC3'	R 5'TGGTAGATGTGGCGGGTTT3'
ACTB (gDNA)	F 5'CCGTGCTCGATGGGGTACTT3'	R 5'CAGGGCTTCTTGTCCTTTCCTTC3'
Tgfb1 (cDNA)	F 5'CTTCAATACGTCAGACATTCGGG3'	R 5'GTAACGCCAGGAATTGTTGCTA3'
Actb (cDNA)	F 5'CCACCATGTACCCAGGCATT3'	R 5'CAGCTCAGTAACAGTCCGCC3'
Nd4 (mtDNA)	F 5'TCGCCTACTCCTCAGTTAGCCACA3'	R 5'TGATGATGTGAGGCCATGTGCGA3'
Rps18 (gDNA)	F 5'TGTGTTAGGGGACTGGTGGACA3'	R 5'CATCACCCACTTACCCCCAAAA3'
ChIP-P4	F 5'CATCTCCCTCCCACCTCCCT3'	R 5'TGCTCCTCGGCGACTCCTT3'
TGFBR1 (cDNA)	F 5'ACGGCGTTACAGTGTTTCTG3'	R 5'GCACATACAAACGGCCTATCTC3'
SMAD3 (cDNA)	F 5'CCATCTCCTACTACGAGCTGAA3'	R 5'CACTGCTGCATTCCTGTTGAC3'
SNAI1 (cDNA)	F 5'TCGGAAGCCTAACTACAGCGA3'	R 5'AGATGAGCATTGGCAGCGAG3'
SLUG (cDNA)	F 5'CGAACTGGACACACATACAGTG3'	R 5'CTGAGGATCTCTGGTTGTGGT3'
CDH1 (cDNA)	F 5'ATTTTTCCCTCGACACCCGAT3'	R 5'TCCCAGGCGTAGACCAAGA3'
VIMENTIN (cDNA)	F 5'TGCCGTTGAAGCTGCTAACTA3'	R 5'CCAGAGGGAGTGAATCCAGATTA3'
ND1 (cDNA)	F 5'ATCAAACTCAAACTACGCCCTG3'	R 5'TGTTGTGATAAGGGTGGAGAGG3'
ND3 (cDNA)	F 5'GAAAAATCCACCCCTTACGAGTG3'	R 5'TTGTTTGTAGGGCTCATGGTAGG3'
NDUFV1 (cDNA)	F 5'TGACTGGTACAAGACAAAGGAGA3'	R 5'TGCCATCTGAGGGCTTATTCA3'
NDUFS2 (cDNA)	F 5'GTCCGATTGCCGATTCAGC3'	R 5'GCTTGGGTACATAACAGCTCC3'
SDHA (cDNA)	F 5'CAGCATGTGTTACCAAGCTGT3'	R 5'GGTGTCGTAGAAATGCCACCT3'
SDHB (cDNA)	F 5'ACAGCTCCCCGTATCAAGAAA3'	R 5'GCATGATCTTCGGAAGGTCAA3'
CYTB (cDNA)	F 5'ACAATTATACCCTAGCCAACCCC3'	R 5'TGGATAGTAATAGGGCAAGGACG3'
UQCRC1 (cDNA)	F 5'GGGGCACAAGTGCTATTGC3'	R 5'GTTGTCCAGCAGGCTAACC3'
COX1 (cDNA)	F 5'CACGAGCATATTTCACCTCCG3'	R 5'TTCATATTGCTTCCGTGGAGTG3'
COX2 (cDNA)	F 5'TCCTAGTCCTGTATGCCCTTTTC3'	R 5'GTAAAGGATGCGTAGGGATGG3'
COX4I1 (cDNA)	F 5'GGCGGCAGAATGTTGGCTA3'	R 5'AGCTCGTACACACACAGAGG3'
COX6A1 (cDNA)	F 5'GGTAGTTGGTGTGTCCTCGG3'	R 5'TGGTGCGACTTCAGGTACAC3'

Supplementary Table 4 Primers for mtDNA identification

**Human Mitochondrial Primers**		
45 amplicons	Sequence	
1	F 5'CACCCTATTAACCACTCACG3'	R 5'TGAGATTAGTAGTATGGGAG3'
2	F 5'ACAAAGAACCCTAACACCAGC3'	R 5'ACTTGGGTTAATCGTGTGACC3'
3	F 5'CATCAAGCACGCAGCAATG3'	R 5'AATCCACCTTCGACCCTTAAG3'
4	F 5'CTCACCACCTCTTGCTCAGC3'	R 5'GCCAGGTTTCAATTTCTATCG3'
5	F 5'TGCACTTGGACGAACCAGAG3'	R 5'TGTTGAGCTTGAACGCTTTC3'
6	F 5'GAGGAACAGCTCTTTGGACAC3'	R 5'AGAGACAGCTGAACCCTCGTG3'
7	F 5'CACTGTCAACCCAACACAGG3'	R 5'ATGTCCTGATCCAACATCGAG3'
8	F 5'CCCAACCTCCGAGCAGTACATG3'	R 5'AGAAGAGCGATGGTGAGAGC3'
9	F 5'ACTACAACCCTTCGCTGACG3'	R 5'TGAAGCCTGAGACTAGTTCGG3'
10	F 5'GCCTAGCCGTTTACTCAATCC3'	R 5'TGAGTTGGTCGTAGCGGAATC3'
11	F 5'TCAGGCTTCAACATCGAATACG3'	R 5'TTATGGTTCATTGTCCGGAGAG3'
12	F 5'TTGGTTATACCCTTCCCGTAC3'	R 5'GTTTAATCCACCTCAACTGCC3'
13	F 5'CCCTTTCACTTCTGAGTCCCAG3'	R 5'AGGGCTTTGAAGGCTCTTG3'
14	F 5'CACCATCACCCTCCTTAACC3'	R 5'GCTGAGTGAAGCATTGGACTG3'
15	F 5'TAAGCACCCTAATCAACTGGC3'	R 5'GCCTCCACTATAGCAGATGCG3'
16	F 5'CTCTAAGCCTCCTTATTCGAGC3'	R 5'ATAGTGATGCCAGCAGCTAGG3'
17	F 5'GCCATAACCCAATACCAAACG3'	R 5'TGGGCTACAACGTAGTACGTG3'
18	F 5'GGCTTCCTAGGGTTTATCGTG3'	R 5'TTTCATGTGGTGTATGCATCG3'
19	F 5'GAGGCTTCATTCACTGATTTCC3'	R 5'GGGCAGGATAGTTCAGACGG3'
20	F 5'TATCACCTTTCATGATCACGC3'	R 5'GACGATGGGCATGAAACTG3'
21	F 5'TGAACCTACGAGTACACCGACTAC3'	R 5'AAGTTAGCTTTACAGTGGGCTCTAG3'
22	F 5'CGGTCAATGCTCTGAAATCTGTG3'	R 5'CATTGTTGGGTGGTGATTAGTCG3'
23	F 5'CTGTTCGCTTCATTCATTGCC3'	R 5'GTGGCGCTTCCAATTAGGTG3'
24	F 5'CCCACTTCTTACCACAAGGC3'	R 5'GTGCTTTCTCGTGTTACATCG3'
25	F 5'TTTCACTTCCACTCCATAACGC3'	R 5'GAAAGTTGAGCCAATAATGACG3'
26	F 5'AGTCTCCCTTCACCATTTCCG3'	R 5'AAAGGAGGGCAATTTCTAGATC3'
27	F 5'ACTACCACAACTCAACGGCTAC3'	R 5'GGAGGATATGAGGTGTGAGCG3'
28	F 5'GGATTAGACTGAACCGAATTGG3'	R 5'CATCGGGTGATGATAGCCAAG3'
29	F 5'GTCTCAATCTCCAACACATATGG3'	R 5'TGTTGTGAGTGTAAATTAGTGCG3'
30	F 5'AACGCCACTTATCCAGTGAACC3'	R 5'CTGTTTGTCGTAGGCAGATGG3'
31	F 5'GACTCCCTAAAGCCCATGTCG3'	R 5'TTGATCAGGAGAACGTGGTTAC3'
32	F 5'ACGAACGCACTCACAGTCG3'	R 5'AAGCCTCTGTTGTCAGATTCAC3'
33	F 5'TGCTAGTAACCACGTTCTCCTG3'	R 5'GATATCGCCGATACGGTTG3'
34	F 5'TTCATCCCTGTAGCATTGTTCG3'	R 5'AGCGGATGAGTAAGAAGATTCC3'
35	F 5'TTGCTCATCAGTTGATGATACG3'	R 5'TTGAAGAAGGCGTGGGTACAG3'
36	F 5'CACTCTGTTCGCAGCAGTCTG3'	R 5'TCGAGTGCTATAGGCGCTTGTC3'
37	F 5'CATCATCGAAACCGCAAAC3'	R 5'TGTGATGCTAGGGTAGAATCCG3'
38	F 5'TTTCTCCAACATACTCGGATTC3'	R 5'TTAGCGATGGAGGTAGGATTGG3'
39	F 5'ACAAACAATGTTCAACCAGTAAC3'	R 5'TGAGGCGTCTGGTGAGTAGTGC3'
40	F 5'TCCAAAGACAACCATCATTCC3'	R 5'CGTGAAGGTAGCGGATGATTC3'
41	F 5'TACTCACCAGACGCCTCAAC3'	R 5'TTATCGGAATGGGAGGTGATTC3'
42	F 5'AGTCCCACCCTCACACGATTC3'	R 5'ACTGGTTGTCCTCCGATTCAGG3'
43	F 5'CGCCTACACAATTCTCCGATC3'	R 5'CGGTTGTTGATGGGTGAGTC3'
44	F 5'TTAACTCCACCATTAGCACC3'	R 5'GCGAGGAGAGTAGCACTCTTG3'
45	F 5'TACATTACTGCCAGCCACCATG3'	R 5'TTAAGTGCTGTGGCCAGAAG3'
Long-range PCR	Sequence	
LRP1	F 5'ACATAGCACATTACAGTCAAATCCCTTCTCGTCCCC3'	R 5'TGAGATTGTTTGGGCTACTGCTCGCAGTGC3'
LRP2	F 5'TACTCAATCCTCTGATCAGGGTGAGCATCAAACTC3'	R 5'GCTTGGATTAAGGCGACAGCGATTTCTAGGATAGT3'
LRP3	F 5'TCATTTTTATTGCCACAACTAACCTCCTCGGACTC3'	R 5'CGTGATGTCTTATTTAAGGGGAACGTGTGGGCTAT3'
**Murine Mitochondrial Primers**		
Gene	Sequence	
Cox3	F 5'ATGACCCACCAAACTCATGC3'	R 5'AGTATCATGCTGCGGCTTC3'
Cytb	F 5'CTCATTCATTGACCTACCTGCC3'	R 5'AGTATGAGATGGAGGCTAGTTGG3'
Nd2	F 5'ATAAATCCTATCACCCTTGCCATC3'	R 5'CTAGGTAATTAGTTGGGGGGCTAG3'
