# Supplementary material for: Mitochondrial genome transfer drives metabolic reprogramming in adjacent colonic epithelial cells promoting TGFβ1-mediated tumor progression
Source: Nat Commun. 2024 Apr 30;15:3653. doi: 10.1038/s41467-024-48100-y (PMC11061154; doi:10.1038/s41467-024-48100-y)
Supplement: Supplementary file 3 — Description of Additional Supplementary Files [file 41467_2024_48100_MOESM3_ESM.pdf]

Description of Additional Supplementary Files

File Name: Supplementary Data 1
Description: GSEA report of our RNA-sequencing data based on hallmark gene sets. GSEA was performed using a two-sided permutation test.

File Name: Supplementary Data 2
Description: The report of our RNA-sequencing data using KEGG pathway enrichment analysis based on a two-sided hypergeometric test.
