# Supplementary material for: Mitochondrial genome transfer drives metabolic reprogramming in adjacent colonic epithelial cells promoting TGFβ1-mediated tumor progression
Source: Nat Commun. 2024 Apr 30;15:3653. doi: 10.1038/s41467-024-48100-y (PMC11061154; doi:10.1038/s41467-024-48100-y)
Supplement: Supplementary file 6 — Reporting Summary [file 41467_2024_48100_MOESM6_ESM.pdf]

nature portfolio

Corresponding author(s): Dongwang Yan

Last updated by author(s): Mar 13, 2024

Reporting Summary

Nature Portfolio wishes to improve the reproducibility of the work that we publish. This form provides structure for consistency and transparency in reporting. For further information on Nature Portfolio policies, see our Editorial Policies and the Editorial Policy Checklist.

Statistics

For all statistical analyses, confirm that the following items are present in the figure legend, table legend, main text, or Methods section.

n/a	Confirmed	
☐	☒	The exact sample size (*n*) for each experimental group/condition, given as a discrete number and unit of measurement
☐	☒	A statement on whether measurements were taken from distinct samples or whether the same sample was measured repeatedly
☐	☒	The statistical test(s) used AND whether they are one- or two-sided<br>*Only common tests should be described solely by name; describe more complex techniques in the Methods section.*
☒	☐	A description of all covariates tested
☐	☒	A description of any assumptions or corrections, such as tests of normality and adjustment for multiple comparisons
☐	☒	A full description of the statistical parameters including central tendency (e.g. means) or other basic estimates (e.g. regression coefficient) AND variation (e.g. standard deviation) or associated estimates of uncertainty (e.g. confidence intervals)
☐	☒	For null hypothesis testing, the test statistic (e.g. *F*, *t*, *r*) with confidence intervals, effect sizes, degrees of freedom and *P* value noted<br>*Give P values as exact values whenever suitable.*
☒	☐	For Bayesian analysis, information on the choice of priors and Markov chain Monte Carlo settings
☒	☐	For hierarchical and complex designs, identification of the appropriate level for tests and full reporting of outcomes
☐	☒	Estimates of effect sizes (e.g. Cohen's *d*, Pearson's *r*), indicating how they were calculated

Our web collection on statistics for biologists contains articles on many of the points above.

Software and code

Policy information about availability of computer code

Data collection	The images of EVs and mitochondria were captured using an H7500 transmission electron microscope (Hitachi, Tokyo, Japan). NTA analysis was performed with a ZetaView PMX110 (Particle Metrix, Meerbusch, Germany). The protein bands were visualized using a Tanon 5200 Chemiluminescent Imaging System (Tanon, Shanghai, China). ELISA assays were performed using a Varioskan Multimode Microplate Reader (Thermo Fisher Scientific, Waltham, MA, USA). qPCR was carried out on a QuantStudio 6 Flex Real-Time PCR System (Applied Biosystems, Foster City, CA, USA). The mIHC slides were imaged by an SP8 confocal microscope (Leica Microsystems, Buffalo Grove, IL, USA). Canonical PCR was performed on a ProFlex PCR System (Applied Biosystems) and the PCR products were visualized using a Tanon 1600 Gel Imaging System (Tanon). The EdU images were captured by a DMi8 inverted microscope (Leica). CCK8, SRB, cell viability, and Caspase3 activity assays were performed using a Varioskan Multimode Microplate Reader (Thermo Fisher Scientific). The percentage of apoptotic cells was quantified by applying the TUNEL methods on a DMi8 inverted microscope (Leica) . Migrated or Invaded cell counting was performed under a DMi8 inverted microscope (Leica). Immunofluorescence and EV uptake were observed by an SP8 confocal microscope (Leica). The mitochondrial activity was detected using a Seahorse analyzer (Seahorse Bioscience, Billerica, MA, USA). Mitochondrial membrane potential, mitochondrial ROS, colon tissue ROS, respiratory complex activities, mitochondrial mass, and luciferase activities were determined using a Varioskan Multimode Microplate Reader (Thermo Fisher Scientific). Total ROS was measured on an Accuri C6 flow cytometer (BD Biosciences). Mitochondrial transfer was observed with a DMi8 inverted microscope (Leica). RNA sequencing was performed on a NovaSeq 6000 platform (Illumina, San Diego, CA, USA).
Data analysis	The mIHC fluorescence intensity was analyzed by ImageJ v1.52n (National Institutes of Health, Bethesda, MD, USA). The mtDNA sequence was analyzed using SnapGene v4.1.9 (GSL Biotech, Chicago, IL, USA). Flow cytometry data in FCS format, generated by BD Accuri C6 Software v1.0.264.21 (BD Biosciences), were analyzed utilizing FlowJo v10.0.7 (BD Biosciences). The raw data of RNA-seq were filtered with fastp v0.22.0 (OpenGene, GitHub). The filtered reads were mapped to the reference genome via HISAT2 v2.1.0 (Johns Hopkins University, Baltimore, MD, USA). Gene expression levels were calculated by HTSeq v0.9.1 (Python Package Index). Then difference expression of genes

was analyzed by DESeq v1.38.3 (Fred Hutchinson Cancer Research Center, Seattle, WA, USA). ClusterProfiler v4.6.0 (Southern Medical University, Guangzhou, China) was used to carry out the enrichment analysis of the KEGG pathway. GSEA v4.1.0 (Broad Institute, Cambridge, MA, USA) was used for GSEA enrichment analysis. Statistical analyses were performed using SPSS Statistics v25.0 (IBM, Chicago, IL, USA) and GraphPad Prism v8.4.3 (GraphPad Software, San Diego, CA, USA).

For manuscripts utilizing custom algorithms or software that are central to the research but not yet described in published literature, software must be made available to editors and reviewers. We strongly encourage code deposition in a community repository (e.g. GitHub). See the Nature Portfolio guidelines for submitting code & software for further information.

Data

Policy information about availability of data

All manuscripts must include a data availability statement. This statement should provide the following information, where applicable:

- Accession codes, unique identifiers, or web links for publicly available datasets
- A description of any restrictions on data availability
- For clinical datasets or third party data, please ensure that the statement adheres to our policy

The RNA-seq data generated in this study have been deposited in the GEO database under accession code GSE233326. Analyses involving data from TCGA and GTEx databases were conducted using the GEPIA portal, accessible at http://gepia.cancer-pku.cn. The interaction analysis between transcription factors and the TGFβ1 promoter utilized the publicly accessible databases Cistrome DB at http://cistrome.org/db/#/ and AliBaba2.1 at http://gene-regulation.com/pub/programs/alibaba2/index.html. Source data are provided with this paper. Uncropped gels are presented in the Source Data file.

Research involving human participants, their data, or biological material

Policy information about studies with human participants or human data. See also policy information about sex, gender (identity/presentation), and sexual orientation and race, ethnicity and racism.

Reporting on sex and gender	Serum and tissue samples from CC patients (female 32, male 50) or healthy donors (female 8, male 11) were obtained at Shanghai General Hospital (Shanghai, China). The gender was determined by self-reporting.
Reporting on race, ethnicity, or other socially relevant groupings	All human participants involved in this study are Chinese people who belong to the yellow race.
Population characteristics	Patients in this cohort ranged from 36-97 years old; the cohort included 50 males and 32 females, 13 cases in stage I, 24 cases in stage II, 25 cases in stage III, and 20 cases in stage IV. The healthy donors ranging from 34-68 years old included 8 females and 11 males. More detailed clinical information of individual participants, including age, sex, grade of differentiation, and tumor-node-metastasis (TNM) staging, are listed in the Supplementary Table 1.
Recruitment	These patients were all newly diagnosed patients with CC who underwent surgical resection and had received no prior treatment for this disease, including chemotherapy, radiotherapy, targeted therapy, or biological therapy. All the participants were recruited at Shanghai General Hospital from 2020 to 2022. There was no self-selection bias in this study.
Ethics oversight	This study was approved by the Ethics Committee of Shanghai General Hospital (Research Ethics Approval Code: 2020SQ150) and each participant provided informed consent before enrolling in the study.

Note that full information on the approval of the study protocol must also be provided in the manuscript.

Field-specific reporting

Please select the one below that is the best fit for your research. If you are not sure, read the appropriate sections before making your selection.

☒ Life sciences ☐ Behavioural & social sciences ☐ Ecological, evolutionary & environmental sciences

For a reference copy of the document with all sections, see nature.com/documents/nr-reporting-summary-flat.pdf

Life sciences study design

All studies must disclose on these points even when the disclosure is negative.

Sample size	No statistical methods were used to predetermine sample size. In each experiment, sample size were determined based on the same experiment with the same group number performed in the previous paper. Sample sizes were indicated in the legend of each Figure and Supplementary Figure. For cell line experiments, at least 3 biological replicates were used; For mice experiments, 6 biological replicates were used.
Data exclusions	No data were excluded from the analyses.
Replication	All data analysis and experimental findings are reproducible. Data reported in this manuscript were reproduced with at least 3 biologically independent replicates for the in vitro experiments and 6 independent mice per group.
Randomization	All cells and mice were randomly allocated into experimental groups. Male C57BL/6J mice of the same age and comparable body weight were

Randomization	randomly assigned to different experimental groups to ensure homogeneity across the conditions being tested. For human participants, this study is an observational study and does not need to adopt randomization.
Blinding	The investigators were not blinded to group allocation during data collection or analysis. Blinding was not required because the data are quantitative and do not require subjective analysis. Our experiments did not involve subjective outcome measures that could be influenced by the participants' or researchers' expectations. Therefore, the added complexity of a double-blind methodology would not significantly contribute to the validity of our results.

Reporting for specific materials, systems and methods

We require information from authors about some types of materials, experimental systems and methods used in many studies. Here, indicate whether each material, system or method listed is relevant to your study. If you are not sure if a list item applies to your research, read the appropriate section before selecting a response.

Materials & experimental systems

n/a	Involved in the study	
☐	☒	Antibodies
☐	☒	Eukaryotic cell lines
☒	☐	Palaeontology and archaeology
☐	☒	Animals and other organisms
☒	☐	Clinical data
☒	☐	Dual use research of concern
☒	☐	Plants

Methods

n/a	Involved in the study	
☒	☐	ChIP-seq
☐	☒	Flow cytometry
☒	☐	MRI-based neuroimaging

Antibodies

Antibodies used

Primary antibodies

CD63	1:500 (IB)	Rabbit polyclonal	Proteintech (25682-1-AP)
CD9	1:2,000 (IB)	Rabbit polyclonal	Proteintech (20597-1-AP)
TSG101	1:10,000 (IB)	Rabbit polyclonal	Proteintech (28283-1-AP)
Calnexin	1:5,000 (IB)	Rabbit polyclonal	Proteintech (10427-2-AP)
ALIX	1:20,000 (IB)	Rabbit polyclonal	Proteintech (12422-1-AP)
GM130	1:10,000 (IB)	Rabbit polyclonal	Proteintech (11308-1-AP)
ACTB	1:5,000 (IB)	Rabbit polyclonal	Proteintech (20536-1-AP)
H3	1:10,000 (IB)	Rabbit polyclonal	Proteintech (17168-1-AP)
TFAM	1:20,000 (IB)	Rabbit polyclonal	Proteintech (22586-1-AP)
RelA	1:1,000 (IB);1:100 (ChIP);1:800 (IF)	Rabbit monoclonal (D14E12)	Cell Signaling Technology (#8242)
THBS1	1:100 (IF)	Rabbit monoclonal (EPR22927-54)	Abcam (ab267388)
ITGAV	1:500 (IF)	Rabbit monoclonal (EPR16800)	Abcam (ab179475)
IKKβ	1:1,000 (IB)	Rabbit monoclonal (D30C6)	Cell Signaling Technology (#8943)
p-IKKβ	1:500 (IB)	Rabbit polyclonal	ABclonal (AP1237)
IκBα	1:1,000 (IB)	Rabbit monoclonal (44D4)	Cell Signaling Technology (#4812)
p-IκBα	1:1,000 (IB)	Rabbit monoclonal (14D4)	Cell Signaling Technology (#2859)
ND1	1:1,000 (IB);1:1,000 (mIHC)	Rabbit polyclonal	ABclonal (A17967)
CYTB	1:1,000 (IB);1:1,000 (mIHC)	Rabbit polyclonal	ABclonal (A17966)
COX1	1:500 (IB);1:2,000 (mIHC)	Rabbit polyclonal	ABclonal (A17889)
ATP5A1	1:5,000 (IB)	Rabbit polyclonal	Proteintech (14676-1-AP)
SDHA	1:5,000 (IB)	Rabbit polyclonal	Proteintech (14865-1-AP)
Smad2	1:1,000 (IB)	Rabbit monoclonal (D43B4)	Cell Signaling Technology (#5339)
p-Smad2	1:1,000 (IB)	Rabbit monoclonal (E8F3R)	Cell Signaling Technology (#18338)
Smad3	1:1,000 (IB)	Rabbit monoclonal (C67H9)	Cell Signaling Technology (#9523)
p-Smad3	1:1,000 (IB)	Rabbit monoclonal (C25A9)	Cell Signaling Technology (#9520)
E-cadherin	1:1,000 (IB);1:2,000 (mIHC)	Rabbit monoclonal (24E10)	Cell Signaling Technology (#3195)
Vimentin	1:1,000 (IB);1:1,000 (mIHC)	Rabbit polyclonal	ABclonal (A2584)
Snai1	1:1,000 (IB);1:500 (mIHC)	Rabbit polyclonal	ABclonal (A5243)
TIM23	1:2,000 (IB)	Rabbit polyclonal	Proteintech (11123-1-AP)
HNRNPU	1:5,000 (IB)	Rabbit polyclonal	Proteintech (14599-1-AP)
Cleaved-PARP	1:1,000 (IB)	Rabbit monoclonal (D64E10)	Cell Signaling Technology (#5625)
Cleaved-Caspase3	1:1,000 (IB)	Rabbit monoclonal (5A1E)	Cell Signaling Technology (#9664)
Calreticulin	1:1,000 (IB)	Rabbit polyclonal	Proteintech (10292-1-AP)
F4/80	1:20,000 (mIHC)	Rabbit polyclonal	Proteintech (29414-1-AP)
Ly6G	1:5,000 (mIHC)	Rabbit monoclonal (EPR22909-135)	Abcam (ab238132)
Epcam	1:1,000 (IF)	Rabbit polyclonal	Proteintech (21050-1-AP)

Secondary antibodies

CY3-conjugated	1:200 (IF)	goat anti-rabbit IgG	Affinity Biosciences (#S0011)
HRP-conjugated	1:5,000 (IB)	goat anti-rabbit IgG	Affinity Biosciences (#S0001)
HRP-conjugated	ready-to-use (mIHC)	goat anti-rabbit IgG	Panovue (10079100050)

Validation

These are all commercially obtained antibodies that had been validated by manufacturers. Validation statements and references can be found in the manufacturers' websites.

Primary antibodies
CD63 Rabbit polyclonal Proteintech (25682-1-AP) https://www.ptgcn.com/products/CD63-Antibody-25682-1-AP.htm
CD9 Rabbit polyclonal Proteintech (20597-1-AP) https://www.ptgcn.com/products/CD9-Antibody-20597-1-AP.htm
TSG101 Rabbit polyclonal Proteintech (28283-1-AP) https://www.ptgcn.com/products/TSG101-Antibody-28283-1-AP.htm
Calnexin Rabbit polyclonal Proteintech (10427-2-AP) https://www.ptgcn.com/products/CANX-Antibody-10427-2-AP.htm
ALIX Rabbit polyclonal Proteintech (12422-1-AP) https://www.ptgcn.com/products/PDCD6IP-Antibody-12422-1-AP.htm
GM130 Rabbit polyclonal Proteintech (11308-1-AP) https://www.ptgcn.com/products/GOLGA2,GM130-Antibody-11308-1-AP.htm
ACTB Rabbit polyclonal Proteintech (20536-1-AP) https://www.ptgcn.com/products/ACTB-Antibody-20536-1-AP.htm
H3 Rabbit polyclonal Proteintech (17168-1-AP) https://www.ptgcn.com/products/Histone-H3-Antibody-17168-1-AP.htm
TFAM Rabbit polyclonal Proteintech (22586-1-AP) https://www.ptgcn.com/products/TFAM-Antibody-22586-1-AP.htm
RelA Rabbit monoclonal Cell Signaling Technology (#8242) https://www.cellsignal.com/products/primary-antibodies/nf-kb-p65-d14e12-xp-174-rabbit-mab/8242
THBS1 Rabbit monoclonal Abcam (ab267388) https://www.abcam.cn/products/primary-antibodies/thrombospondin-1-antibody-epr22927-54-ab267388.html
ITGAV Rabbit monoclonal Abcam (ab179475) https://www.abcam.cn/products/primary-antibodies/integrin-alpha-v-antibody-epr16800-ab179475.html
IKKβ Rabbit monoclonal Cell Signaling Technology (#8943) https://www.cellsignal.com/products/primary-antibodies/ikkb-d30c6-rabbit-mab/8943
p-IKKβ Rabbit polyclonal ABclonal (AP1237) https://abclonal.com.cn/catalog/AP1237
IκBα Rabbit monoclonal Cell Signaling Technology (#4812) https://www.cellsignal.com/products/primary-antibodies/ikba-44d4-rabbit-mab/4812
p-IκBα Rabbit monoclonal Cell Signaling Technology (#2859) https://www.cellsignal.com/products/primary-antibodies/phospho-ikba-ser32-14d4-rabbit-mab/2859
ND1 Rabbit polyclonal ABclonal (A17967) https://abclonal.com.cn/catalog/A17967
CYTB Rabbit polyclonal ABclonal (A17966) https://abclonal.com.cn/catalog/A17966
COX1 Rabbit polyclonal ABclonal (A17889) https://abclonal.com.cn/catalog/A17889
ATP5A1 Rabbit polyclonal Proteintech (14676-1-AP) https://www.ptgcn.com/products/ATP5A1-Antibody-14676-1-AP.htm
SDHA Rabbit polyclonal Proteintech (14865-1-AP) https://www.ptgcn.com/products/SDHA-Antibody-14865-1-AP.htm
Smad2 Rabbit monoclonal Cell Signaling Technology (#5339) https://www.cellsignal.com/products/primary-antibodies/smad2-d43b4-xp-174-rabbit-mab/5339
p-Smad2 Rabbit monoclonal Cell Signaling Technology (#18338) https://www.cellsignal.com/products/primary-antibodies/phospho-smad2-ser465-ser467-e8f3r-rabbit-mab/18338
Smad3 Rabbit monoclonal Cell Signaling Technology (#9523) https://www.cellsignal.com/products/primary-antibodies/smad3-c67h9-rabbit-mab/9523
p-Smad3 Rabbit monoclonal Cell Signaling Technology (#9520) https://www.cellsignal.com/products/primary-antibodies/phospho-smad3-ser423-425-c25a9-rabbit-mab/9520
E-cadherin Rabbit monoclonal Cell Signaling Technology (#3195) https://www.cellsignal.com/products/primary-antibodies/e-cadherin-24e10-rabbit-mab/3195
Vimentin Rabbit polyclonal ABclonal (A2584) https://abclonal.com.cn/catalog/A2584
Snai1 Rabbit polyclonal ABclonal (A5243) https://abclonal.com.cn/catalog/A5243
TIM23 Rabbit polyclonal Proteintech (11123-1-AP) https://www.ptgcn.com/products/TIMM23-Antibody-11123-1-AP.htm
HNRNPU Rabbit polyclonal Proteintech (14599-1-AP) https://www.ptgcn.com/products/HNRNPU-Antibody-14599-1-AP.htm
Cleaved-PARP Rabbit monoclonal Cell Signaling Technology (#5625) https://www.cellsignal.com/products/primary-antibodies/cleaved-parp-asp214-d64e10-xp-174-rabbit-mab/5625
Cleaved-Caspase3 Rabbit monoclonal Cell Signaling Technology (#9664) https://www.cellsignal.com/products/primary-antibodies/cleaved-caspase-3-asp175-5a1e-rabbit-mab/9664
Calreticulin Rabbit polyclonal Proteintech (10292-1-AP) https://www.ptgcn.com/products/CALR-Antibody-10292-1-AP.htm
F4/80 Rabbit polyclonal Proteintech (29414-1-AP) https://www.ptgcn.com/products/F4-80-Antibody-29414-1-AP.htm
Ly6G Rabbit monoclonal Abcam (ab238132) https://www.abcam.cn/products/primary-antibodies/ly6g-antibody-epr22909-135-ab238132.html
Epcam Rabbit polyclonal Proteintech (21050-1-AP) https://www.ptgcn.com/products/EPCAM-Antibody-21050-1-AP.htm

Secondary antibodies
CY3-conjugated goat anti-rabbit IgG Affinity Biosciences (#S0011) https://www.affbiotech.cn/goods-15085-S0011-Goat_Anti_Rabbit_IgG_H_L_CY3_conjugated.html
HRP-conjugated goat anti-rabbit IgG Affinity Biosciences (#S0001) https://www.affbiotech.cn/goods-6302-S0001-Goat_Anti_Rabbit_IgG_H_L_HRP.html
HRP-conjugated goat anti-rabbit IgG Panovue (10079100050) https://u5mpootmgx.jiandaoyun.com/dash/611cc8d79bbb8a0008295ad3

Eukaryotic cell lines

Policy information about cell lines and Sex and Gender in Research

Cell line source(s)

Human embryonic kidney cell line 293T (CL-0005), human CC cell lines SW480 (CL-0223B), HCT116 (CL-0096), RKO (CL-0196), HT29 (CL-0118), and SW620 (CL-0225B) were provided by Procell (Wuhan, China). Human normal colonic epithelial cells FHC and murine CC cells MC38 were kindly gifted from Dr. Jikun Li (Shanghai Jiao Tong University School of Medicine).

Authentication

All cell lines were authenticated by STR profiling.

Mycoplasma contamination	All cell lines were confirmed negative for mycoplasma contamination.
Commonly misidentified lines (See ICLAC register)	No misdentified cell lines were used in this study.

Animals and other research organisms

Policy information about studies involving animals; ARRIVE guidelines recommended for reporting animal research, and Sex and Gender in Research

Laboratory animals	Mouse, C57BL/6J, male, 6 to 8 weeks old (GemPharmatech, Nanjing, China). Mice housed in SPF conditions were maintained in a temperature-regulated facility at 22 degrees Celsius with 50-60% humidity, adhering to a 12-hour light/dark cycle. They had ad libitum access to Picolab Rodent Diet 20 (product code #5053).
Wild animals	No wild animals were used in this study.
Reporting on sex	Sex was not considered in this study. Male mice were used due to the consistency of their hormone levels, which could reduce variability in experimental results.
Field-collected samples	The study did not involve samples collected from the field.
Ethics oversight	All animal experiments were approved by the Shanghai General Hospital, Shanghai Jiao Tong University School of Medicine Animal Care and Use Committee (IACUC No. 2022AW015).

Note that full information on the approval of the study protocol must also be provided in the manuscript.

Flow Cytometry

Plots

Confirm that:

☒ The axis labels state the marker and fluorochrome used (e.g. CD4-FITC).

☒ The axis scales are clearly visible. Include numbers along axes only for bottom left plot of group (a 'group' is an analysis of identical markers).

☒ All plots are contour plots with outliers or pseudocolor plots.

☒ A numerical value for number of cells or percentage (with statistics) is provided.

Methodology

Sample preparation	Total ROS levels were measured with a ROS Assay Kit (Beyotime, Shanghai, China) based on the manufacturer's instructions.
Instrument	Accuri C6 flow cytometer (BD Biosciences)
Software	BD Accuri C6 Software v1.0.264.21 (BD Biosciences) and FlowJo v10.0.7 (BD Biosciences)
Cell population abundance	In total, at least 10,000 events were recorded for each sample and analyzed using the above softwares.
Gating strategy	Dead cells and debris were removed based on SSC-A and FSC-A, and doublets were excluded based on FSC-A vs. FSC-H. Control cells without staining were used as negative control.

☒ Tick this box to confirm that a figure exemplifying the gating strategy is provided in the Supplementary Information.
