# Supplementary material for: Mitochondrial genome transfer drives metabolic reprogramming in adjacent colonic epithelial cells promoting TGFβ1-mediated tumor progression
Source: Nat Commun. 2024 Apr 30;15:3653. doi: 10.1038/s41467-024-48100-y (PMC11061154; doi:10.1038/s41467-024-48100-y)
Supplement: Supplementary file 2 — Peer Review File [file 41467_2024_48100_MOESM2_ESM.pdf]

nature portfolio

Peer Review File

Mitochondrial genome transfer drives metabolic reprogramming in adjacent colonic epithelial cells promoting TGFβ1-mediated tumor progression

Open Access This file is licensed under a Creative Commons Attribution 4.0 International License, which permits use, sharing, adaptation, distribution and reproduction in any medium or format, as long as you give appropriate credit to the original author(s) and the source, provide a link to the Creative Commons license, and indicate if changes were made. In the cases where the authors are anonymous, such as is the case for the reports of anonymous peer reviewers, author attribution should be to 'Anonymous Referee' followed by a clear attribution to the source work. The images or other third party material in this file are included in the article's Creative Commons license, unless indicated otherwise in a credit line to the material. If material is not included in the article's Creative Commons license and your intended use is not permitted by statutory regulation or exceeds the permitted use, you will need to obtain permission directly from the copyright holder. To view a copy of this license, visit http://creativecommons.org/licenses/by/4.0/.

REVIEWER COMMENTS

Reviewer #1 (Remarks to the Author); expert in EVs:

Mitochondrial genome transfer drives metabolic reprogramming 1 in adjacent colonic epithelial cells promoting TGFβ1-mediated tumor progression

Bingjie Guan, Youdong Liu, Bowen Xie, Abudushalamu Yalikun, Weiwei Chen, Menghua Zhou1, Qi Gu & Dongwang Yan

In this manuscript, the investigators report that intercellular transfer of complete circular mtDNA via cancer cell-derived EVs results in enhanced OXPHOS and mitochondrial function in adjacent normal CECs as well as in an increase in reactive oxygen species (ROS). Additionally, the investigators demonstrated that ROS activates the NF-κB signaling pathway and drives TGFβ1 transcription and secretion in normal CECs which in turn promotes tumor progression.

Overall, the investigators effectively demonstrate the role of EV-mtDNA in the enhancement of OXPHOS and ROS production in normal colonic epithelial cells, and propose a mechanism by which this drives tumor progression through a ROS-mediated NF-κB and TGFβ1-involved pathway. The manuscript contains a large amount of interesting work. Figures are legible and accurately described the data presented. Summaries and conclusions are clear. Additional strengths include the use of proper controls where possible and acceptable explanations when the proper control was not used. For example, normal colonic mucosa from healthy living individuals were not clinically available due to medical ethics. Instead, the investigators performed experiments assessing mtDNA content in healthy colonic mucosa in healthy mice.

However, several technical issues compromise the validity of most of the conclusions.

The method used to generate mtDNA-depleted (ρ0) cell lines should be described in some detail, and stronger evidence that the cells are stable should be provided. The EV isolation strategy and EV characterization methods, including for sizing and molecular content are suboptimal and do not allow to make any conclusion on what type of EVs are involved in this important process. Also the background does not take in account all the knowledge on EV heterogeneity and how this could affect the distribution of circular mtDNA in different populations of EVs.

Data presented in Figure 4C shows significantly decreased amounts of mtDNA in the SW480 and HCT116 ρ0 cell lines, however the mtDNA is not completely depleted and it is difficult to exclude that the remaining mtDNA is functional.

The investigators used and impressive number of cell lines and conditions to validate their reported fundings. However, no rationale for their use is provided. For example, they should describe why RKO and HT29 cells used for the co-culture experiments. As is, the use of these specific lines seems a bit random.

Reviewer #2 (Remarks to the Author); expert in colorectal cancer and tumour microenvironment:

In this paper, Gua et al study the relevance of tumour-cell derived extracellular vesicles (EVs) in colorectal cancer. They report transfer of intact mitochondrial DNA via EVs to adjacent colonic epithelial cells (CECs), which alters their expression of mitochondrial proteins. As a consequence, the authors find increased OXFOS and ROS which triggers Nfkb signalling to promote TGFB1 expression. In its turn, TGFB1 is secreted and affects the tumour cells to promote disease progression and induction of an EMT-like signature. The study is extensive and well presented. However, the proposed mechanism is not supported by the data and there are several concerns regarding the experimental models.

1. It is well established that extracellular vesicles can contain mtDNA, and that these can enter cells via endocytosis. However, it is unclear how a mitochondrial genome internalized as such will

become functional in host cells. For this to result in the expression of mitochondrial proteins and functional changes to the respiratory chain, this free mtDNA would need to cross the outer and inner mitochondrial membrane, for which no evidence is presented. Hence, all metabolic effects observed by the authors thereafter could be indirect and this entirely undermines the proposed mechanism. The data presented would be more in line with transfer of intact mitochondria but also no evidence is provided for this.

2. The authors show that conditioned medium of cancer cells on FHC cells increases expression of mtDNA encoded genes, and report an increase in OXPHOS. However, they also show that EVs do not affect the levels of nuclear DNA-encoded mitochondrial proteins which are also critical for OXPHOS. It is unclear how this imbalance in proteins can lead to a functional and increased oxidative phosphorylation.

3. The authors use FHC cells as a model for normal CEC, however these are tumorigenic cells with multiple genomic abnormalities (including mutations in TP53). Assays should be performed with primary murine organoids, derived from WT mice.

4. There are several questions/concerns around the in vivo studies. Specifically, regarding the AOM/DSS model
• The authors show increased mtDNA content in normal adjacent tissue (NAT) of tumour bearing animals, compared to healthy mucosa of non-tumour bearing animals. However, there is no data showing that tumours in this model release EVs containing mtDNA.
• The in vitro data suggest a proliferation advantage for CRC cells when cocultured with EV-exposed FHC cells. To validate this in vivo, the authors perform i.p. injection of EVs (3×10e9 EVs, once every 3 days for a total of 60 days) and show increased tumour burden (Figure 5E-G).
o AOM/DSS is a model of inflammation associated CRC. EVs have been shown to trigger an innate immune response. Can the authors show that their regime does not trigger an additional inflammatory response which can affect tumour development?
o Can the authors show that uptake of EVs and increased expression of mitochondrial proteins happens in CECs only, and not in tumour cells which would make it difficult to discriminate between 'CEC-Tumour communication' and direct effects on tumour epithelial cells?
o How representative is the level of such exposure of EVs to a patient setting? Similar question applies to the experiments injecting EVs from MC38 cells into WT mice (Figure 5A,B).
o Which other tissues are affected by these EVs?
• As per the model there should be a gradient of ROS in NAT with higher levels being observed in closer proximity to the tumour. This has not been shown in vivo or in patient samples.

Minor comments

1. The study is based on the literature observation that oxphos is higher in tumour-adjacent colon compared to healthy mucosa. However, this is not a well-established observation, and is only supported by transcriptional profiling, rather than by functional studies (reference 11).

2. What else makes up the EVs shedded by these models? Can this also affect metabolism of NAT?

3. In Figure 6A, it's unclear why a 3 cell-based model was used. RKO cells were cocultured with FHC pretreated with EV from SW480 cells. As per the proposed mechanism, simply co-culturing CRC cells with CEC cells should produce the same results (CRC releases EVs, CECs produce TGFB1). At very least, pretreatment should be done with EVs from the same cell line.

4. Data on shTFAM (Figure S3A) needs to be presented differently to also allow interpretation of shTFAM on cells that were not exposed to EVs (i.e. normalize all data shNT/PBS).

5. Can the authors clarify the results obtained with IMT1B (Fig 3A) and shTFAM (Fig S3A)? Both are strategies to impair mitochondrial replication, yet the results obtained seem opposite.

6. All Seahorse data needs to be normalized.

Reviewer #3 (Remarks to the Author); expert in mitochondria:

The manuscript by Guan et al., is a detailed study of extracellular vesicles (EVs) in terms of mitochondrial DNA (mtDNA) content in EVs from colon cancer cells and its effects in recipient cells, which are in this particular case, cells nearby the tumor. Therefore authors have explored the paracrine action of EVs and found that these EVs, enriched in mtDNA, are able of transferring functional genome that will impact in the content on mitochondrial respiration components in recipient cells, changing their metabolic function. The intra-peritoneal injection of the EVs has a clear impact in colon tumors in mice that are induced by chemical agents.
This is a well-executed work, which includes a number of different techniques to analyze the content and role of mtDNA in cancer development. A major issue here is that authors only envisage a component of EVs having a specific role in tumorigenesis, which has in turn been associated to the ability of other cells to act as a regulator of homeostasis, such as myeloid cells. Is mtDNA the only relevant component having a role here? In the model used by the authors, it seems that these EVs have only effect on the tumor; however, the effect of mtDNA as a DAMP (danger associated molecules) by immune cells (mainly myeloid) in the peritoneum of injected mice has not been addressed. It is required that authors demonstrate that the EVs only impact in the tumor-surrounding cells: do EVs bear a specific receptor or molecule that allow their binding to these cells? Are other cells decorated or modified by the EVs in these mice?

Several other points should be addressed by authors:

1.- An important question that authors address only partially in the manuscript is whether the role described for mtDNA is a general mechanism or a specific effect observed in the particular case of EVs produced by CC cells. A relevant control would be the comparative analysis of the capacity of circulating EVs from HC, which also includes mtDNA among their components.
2.- In general, it is difficult to detect differences in the histopathologic samples showed in the Ms (Fig 1b, 1d, 5c, 6g, 6h, 6j, 8d). Same applies for immunofluorescences (Fig. 2l, 4a, 5a, 7e, 7g). In general, the images showed are too small to observe any kind of difference: these are not evaluable (even if quantification is provided). Merged images without individual channels are not very informative.
3.- In Figure 6a, the authors perform a sequencing of samples treated with SM+ EVs vs PBS and observe an upregulation of the TGFb1 pathway. However, PBS here does not seem to be the best control, as one cannot rule of the effect that the EV itself or any other of its components is having when comparing to PBS. In this sense, the authors should have compared SM+ EVs with EVs that do not bear mitDNA. A validation including this sample, either by transcriptomics or by parallel validation by qPCR of the three conditions of TGFB1-related genes identified in that dataset should be performed.
4.- Can authors test the effect of TGB1 plus EVs? Is this treatment additive and/or synergic?

Minor points :

Which is the level of TFAM protein in the recipient cells of mtDNA?
In Fig 1d: there seems to be some bands missing (as compared to Fig. 1h).
In Figure 1d, why are there some fragments that are not amplified?
Is there any kind of typos in Fig 2h?
Statistical analysis should be provided for the differences in basal and spare respiratory capacities in figure 2i.
In line 38 a reference should be provided.
Lines 260-262 seem to need some editing.
Data not shown should not be included (line 427).

RESPONSE TO REVIEWERS' COMMENTS

Dear Reviewers,

Thank you very much for your professional comments regarding our manuscript. These comments are all valuable and very helpful for revising and improving our manuscript, as well as the important guiding significance to our research. We have studied the comments carefully and made detailed corrections to our manuscript. The reviewer comments are below, and specific concerns have been numbered. Our responses are given in blue, and changes/additions to the manuscript are shown in red text. As detailed below in our point-by-point responses which address all points raised, we believe that we have further strengthened our manuscript.

Yours sincerely,

Dongwang Yan

The responses to the comments point by point:

Reviewer comments:

Reviewer #1; expert in EVs:

In this manuscript, the investigators report that intercellular transfer of complete circular mtDNA via cancer cell-derived EVs results in enhanced OXPHOS and mitochondrial function in adjacent normal CECs as well as in an increase in reactive oxygen species (ROS). Additionally, the investigators demonstrated that ROS activates the NF-κB signaling pathway and drives TGFβ1 transcription and secretion in normal CECs which in turn promotes tumor progression.

Overall, the investigators effectively demonstrate the role of EV-mtDNA in the enhancement of OXPHOS and ROS production in normal colonic epithelial cells, and propose a mechanism by which this drives tumor progression through a ROS-mediated NF-κB and TGFβ1-involved pathway. The manuscript contains a

large amount of interesting work. Figures are legible and accurately described the data presented. Summaries and conclusions are clear. Additional strengths include the use of proper controls where possible and acceptable explanations when the proper control was not used. For example, normal colonic mucosa from healthy living individuals were not clinically available due to medical ethics. Instead, the investigators performed experiments assessing mtDNA content in healthy colonic mucosa in healthy mice.

However, several technical issues compromise the validity of most of the conclusions.

1. The method used to generate mtDNA-depleted (ρ0) cell lines should be described in some detail, and stronger evidence that the cells are stable should be provided. The EV isolation strategy and EV characterization methods, including for sizing and molecular content are suboptimal and do not allow to make any conclusion on what type of EVs are involved in this important process. Also the background does not take in account all the knowledge on EV heterogeneity and how this could affect the distribution of circular mtDNA in different populations of EVs.

Response: Thank you for your insightful comment. We have added the following details of the method used to generate ρ0 cells in lines 453-456 in the revised manuscript: The tumor cell lines devoid of mtDNA (ρ0) were established and cultured as described previously[39]. Briefly, parental cells were grown in medium supplemented with 100 ng mL^{-1} EtBr, 50 μg mL^{-1} uridine, and 1 mM pyruvate for 3 months, followed by verification of mtDNA depletion using qPCR and electron microscopy.

To further prove the stable depletion of mtDNA in the ρ0 cell lines, we continuously monitored mtDNA content in ρ0 and parental cells for 10 weeks. The results confirmed the stable and efficient removal of mtDNA in the ρ0 cell lines established by our method. We have re-supplemented these results in Supplementary Fig. 6 and have written them in lines 214-216 in the revised manuscript as follows: Stable mtDNA deletion, resulting in a typical vacuolar structure in mitochondria, had no effect on the cell proliferative potential (Fig. 4d and Supplementary Fig. 6a-c).

In our study, we obtained EVs by sequential ultracentrifugation, which has been described as a classical method widely used to isolate purified EVs. Referring to the Minimal Information for Studies of Extracellular Vesicles 2018 guidelines (MISEV2018), besides immunoblotting, NTA, and transmission electron microscopy analysis performed already, we additionally detected ALIX (an EV marker) and GM130 (a negative control) to meet the requirement of MISEV2018. Of note, NTA and transmission electron microscopy analysis indicated that most of the collected EVs were under 200 nm in size, in line with the fact that exosomes/small EVs (sEVs, <200 nm) are the main components in the products isolated using this technique [Crewe, C. et al. *Cell* **175**, 695-708.e13 (2018)] (Fig. 1a, e and Supplementary Fig. 1d-f). To further address the proposed issue, we performed sucrose density ultracentrifugation. The EV fractions had extremely lower concentrations of Histone 3 and calreticulin than parental cell lysates, implying that the EVs contained little to no contamination with cellular debris (Supplementary Fig. 2i-m). Intriguingly, EV-mtDNA and protein markers were mainly detected within the density range constant with that published for exosomes (F2-3, 1.13-1.19 g mL^{-1}) (Supplementary Fig. 2a-e). Moreover, GW4869, an exosome inhibitor, dramatically decreased the mtDNA content in the conditional medium of tumor cells (Supplementary Fig. 2f). These new findings provide strong evidence that mtDNA is primarily enriched in exosomes secreted by CC cells. Considering that exosomes consist of traditional subpopulations and non-membranous components termed “exomeres” [Zhang, H. et al. *Nat. Cell Biol*. **20**, 332-343 (2018)], we next investigated the mtDNA-associated exosomal subtype. The exomere markers IDH1 and MTHFD1 (two exomere-specific markers) were barely detected in our isolated EVs (Supplementary Fig. 2g). Besides, the apparent degradation of EV-mtDNA following incubation with DNase plus Triton X-100 indicates that EV-mtDNA is wrapped by the membrane (Fig. 1i). Thus, these results exhibit that mtDNA is mainly enriched in CC cell-secreted membranous exosomes. We realize that the underlying mechanisms of mtDNA-specific distribution in exosomes are appealing. Recent findings suggest the involvement of PINK1, Rab27, and Rab7 in the selective exosomal secretion of mitochondrial components.

However, in a preliminary experiment, we observed no change in exosomal mtDNA enrichment following the regulation of these proteins. Thus, the regulatory mechanism of EV-mtDNA secretion is an exciting subject requiring future dedicated studies.

The results have been supplemented in Fig. 1, Supplementary Fig. 1, and Supplementary Fig. 2, and clarified in lines 109-128 in the revised manuscript as follows: We further characterized the isolated EVs by sucrose density ultracentrifugation. Of note, EV-mtDNA and protein markers were mainly detected within the exosome-containing density range (F2-3, 1.13-1.19 g mL^{-1})[17], and inhibition of exosome secretion by GW4869 sharply decreased the mtDNA content in the tumor CM (Supplementary Fig. 2a-f). These findings, together with the above results that the majority of the collected EVs were under 200 nm in size, indicate that mtDNA is primarily enriched in exosomes secreted by CC cells. Despite exosomes consisting of traditional subpopulations and non-membranous components termed “exomeres”[18], the exomere markers IDH1 and MTHFD1 were barely detected in our isolated EVs (Supplementary Fig. 2g). Moreover, the obvious degradation of EV-mtDNA observed after incubation with DNase in combination with Triton X-100 indicates that EV-mtDNA is wrapped by the membrane, excluding the involvement of non-membranous exomeres (Fig. 1i). We then employed plasmid-safe DNase, which selectively hydrolyzes linear dsDNA and not closed circular dsDNA. Notably, treatment with Triton X-100 and plasmid-safe DNase did not decrease the EV-mtDNA content, strongly supporting the finding that CC cell-derived EV-mtDNA was present as a complete circular dsDNA molecule and not as broken DNA fragments (Fig. 1i). The degradative activity of plasmid-safe DNase toward linear dsDNA was verified (Supplementary Fig. 2h). In addition, the EVs had extremely lower concentrations of Histone 3 and calreticulin than parental cell lysates, implying that there was minimal to no cellular debris contamination of the EVs collected via this method (Supplementary Fig. 2i-m).

2. Data presented in Figure 4C shows significantly decreased amounts of mtDNA

in the SW480 and HCT116 ρ0 cell lines, however the mtDNA is not completely depleted and it is difficult to exclude that the remaining mtDNA is functional.

Response: Thanks for this important comment. To our knowledge, the classical method used in this study for establishing ρ0 cell lines is widely accepted and adopted. As you noted, residual mtDNA in ρ0 cells is commonly detected and challenging to eliminate completely. For example, Nakahira et al. reported that EtBr effectively depleted mtDNA content in macrophages employed for their following studies. However, they still detected a trace amount of mtDNA in ρ0 cells, as shown in Supplementary Fig. 3 of their article.

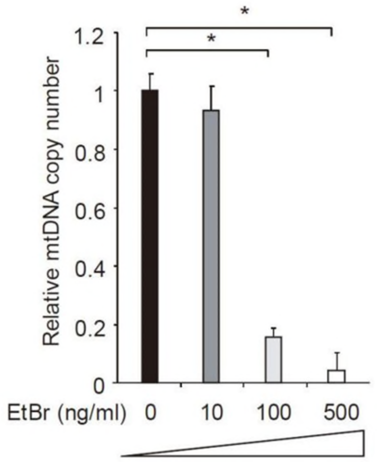

Supplementary Fig. 3 from Nakahira, K. et al. *Nat. Immunol.* **12**, 222-230 (2011)

Notably, ρ0-derived EVs had only about 0.01% of the mtDNA content of wild-type EVs, ensuring the validity of our approach (Fig. 4c). We apologize that we had truncated the vertical axis of histograms in the initial Fig. 4c, giving the impression that the mtDNA deletion efficiency was insufficient, which confused. Given that, we modified the Fig. 4c in the revised manuscript.

Moreover, we showed that mtDNA-depleted EVs lost their function to increase mtDNA copy number and mitochondrial OXPHOS in recipient cells (Fig. 4e-i and Supplementary Fig. 6g-k). To further confirm this, we performed an additional experiment: We incubated FHC cells with tumor ρ0 cell-derived EVs and sequenced the mtDNA in FHC cells. Unlike wild-type counterparts, mtDNA-depleted EVs failed to transfer mtDNA into recipient cells, as shown by the absence of tumor cell-type point mutations in FHC cells (Fig. 4b and Supplementary Fig. 6e, f). Our findings

clearly indicate that these mtDNA-deficient EVs are suitable for investigating the role of mtDNA in intercellular communication. We have added the new results in Supplementary Fig. 6 and have written them in lines 219-220 in the revised manuscript as follows: Sanger sequencing assays demonstrated that ρ0 cell-derived EVs lost the ability to transfer detectable mtDNA into CECs (Supplementary Fig. 6e, f).

3. The investigators used and impressive number of cell lines and conditions to validate their reported fundings. However, no rationale for their use is provided. For example, they should describe why RKO and HT29 cells used for the co-culture experiments. As is, the use of these specific lines seems a bit random.

Response: Thank you for this comment. We realize that the selective use of tumor cell lines should be explained to address this confusion. As shown in Fig. 1j, the mtDNA content in EVs derived from all five CC cell lines was remarkably higher than that in EVs from FHC. Moreover, SW480 or HCT116 secreted EVs with the highest or lowest mtDNA abundance among the tumor cell lines, respectively. Therefore, using EVs derived from SW480 and HCT116, to co-culture with FHC, we aimed to eliminate the possible confounding effect of EV-mtDNA abundance on the outcome of this study. On the other hand, we observed that stimulated FHC cells, in turn, promote the malignancy of tumor cells SW480 and HCT116, as well as another two CC cell lines RKO and HT29 (Supplementary Fig. 9 and Supplementary Fig. 10). To emphasize the generality of this phenomenon, we selected RKO and HT29 cells for further investigation, to exclude the potential concern that this mechanism was restricted to SW480 and HCT116 cells only. Incidentally, the strategy of alternating different tumor cell lines to varying stages of studies has been employed in previous reports [Zhao, S. et al. *J. Extracell. Vesicles* **11**, e12186 (2022)]. The experimental results of treated FHC promoting the malignancy of SW480 and HCT116 have been supplemented in Supplementary Fig. 10 and clarified in lines 262-266 in the revised manuscript as follows: The phenotypic assays suggested that FHC cells educated by mtDNA-sufficient EVs had the most pronounced promotive effects on the

proliferation, migration and invasion abilities of CC cells, which were weakened by EV-mtDNA depletion (Supplementary Fig. 9a-h). Same results were observed in SW480 and HCT116 cells, indicating a widespread phenomenon (Supplementary Fig. 10a-h).

Reviewer #2; expert in colorectal cancer and tumour microenvironment:

In this paper, Guan et al study the relevance of tumour-cell derived extracellular vesicles (EVs) in colorectal cancer. They report transfer of intact mitochondrial DNA via EVs to adjacent colonic epithelial cells (CECs), which alters their expression of mitochondrial proteins. As a consequence, the authors find increased OXFOS and ROS which triggers Nfkb signalling to promote TGFB1 expression. In its turn, TGFB1 is secreted and affects the tumour cells to promote disease progression and induction of an EMT-like signature. The study is extensive and well presented. However, the proposed mechanism is not supported by the data and there are several concerns regarding the experimental models.

1. It is well established that extracellular vesicles can contain mtDNA, and that these can enter cells via endocytosis. However, it is unclear how a mitochondrial genome internalized as such will become functional in host cells. For this to result in the expression of mitochondrial proteins and functional changes to the respiratory chain, this free mtDNA would need to cross the outer and inner mitochondrial membrane, for which no evidence is presented. Hence, all metabolic effects observed by the authors thereafter could be indirect and this entirely undermines the proposed mechanism. The data presented would be more in line with transfer of intact mitochondria but also no evidence is provided for this.

Response: Thanks a lot for this valuable suggestion. We strongly concur with your viewpoint that EV-mtDNA is required to localize to the recipient mitochondria to mediate transcriptional and translational regulation, thereby boosting oxidative

phosphorylation. To address this concern, we isolated mitochondria from the recipient cells co-culturing with murine or human tumor cell-derived EVs. Then we extracted the intramitochondrial mtDNA from the isolated mitochondria for PCR and sequencing. Sanger sequencing detection of murine mtDNA or human tumor cell-specific point mutations in mtDNA suggested that the exogenous mtDNA was actually delivered into the recipient mitochondria via EVs (Fig. 4b and Supplementary Fig. 4d, e). We have rewritten it in lines 197-207 in the revised manuscript as follows: To further confirm this result, we examined mtDNA point mutations by amplifying mtDNA fragments, which were obtained from isolated mitochondria, and then performing Sanger sequencing. Intriguingly, only upon education with EVs derived from SW480 or HCT116 cells did mtDNA in FHC cells' mitochondria exhibit the SW480 or HCT116 cell-type point mutations that were present in the CC cells and their corresponding EVs, providing strong evidence supporting the transfer of EV-mtDNA into mitochondria of CECs (Fig. 4b). Furthermore, FHC cells incubated with EVs derived from the murine CC cell line MC38 simultaneously exhibited intramitochondrial murine and human mtDNA, and the murine mtDNA fragments encoding Cox3, Cytb, and Nd2 were then verified by sequencing, further confirming the ability of CC cell-derived EVs to transfer mtDNA into mitochondria of CECs (Supplementary Fig. 4d, e).

To better address this issue visually, we performed confocal fluorescence microscopy, in which we observed a significant colocalization of exogenous EVs (labeled with PKH67), their carried mtDNA (labeled with EtBr), and the mitochondria of recipient cells (labeled with MitoTracker Red), providing direct evidence for transporting mtDNA into mitochondria by EVs (Fig. 4a). Taken together, we conclude that tumor cell-derived EV-mtDNA can be transferred into mitochondria of the recipient cells. Reasonably, the membranes of EVs can fuse with mitochondrial membranes, leading to the transfer of EVs' cargo into mitochondria. However, the molecular details of EV fusion with recipient organelles, including mitochondria, are interesting and complicated, requiring dedicated in-depth investigations. These new findings have been added in Fig. 4a and discussed in lines 195-197 and 437-439 in the revised

manuscript as follows: More importantly, we observed the colocalization of EVs, EV-carried mtDNA, and the mitochondria of FHC cells, implying that EVs can fuse with recipient cells' mitochondria, consequently transfer mtDNA into them (Fig. 4a). We have confirmed the ability of EVs to transfer mtDNA into recipient mitochondria, however, the detailed mechanism of this process needs further studies.

On the other hand, we investigated the potential for transfer of intact mitochondria from tumor cells to recipient cells. We labeled the mitochondria of tumor cells with two different staining methods and then co-cultured them with FHC cells for an adequate duration. Subsequently, we detected the fluorescence signals in FHC and tumor cell lines. We did not observe any mitochondrial fluorescence signal in the recipient cells. The presence of clear fluorescence signals in both staining methods in tumor cells ruled out the possibility of false negative results owing to fluorescence quenching (Supplementary Fig. 5a-d). Moreover, we incubated tumor cell-derived EVs with FHC cells in a time-dependent manner. We found no alteration in the mitochondrial mass/number in host cells (Supplementary Fig. 5e). These results indicate that colon cancer cells do not transfer intact mitochondria to recipient cells, or at least, the transfer amount was negligible and had no significant biological effects.

Additionally, to the best of our knowledge, the diameter of mitochondria ranging from 0.5 to 10 μm far exceeds the carrying capacity of the EVs we studied, identified as small EVs (< 200 nm in size)[Chen, H. et al. *Theranostics* **7**, 3781-3793 (2017)]. Thus, we demonstrate that the biologically active EVs we focus on in this study transport mtDNA to regulate the recipient cells. The related findings have been included in Supplementary Fig. 5 and addressed in lines 207-211 in the revised manuscript as follows: In addition, following coculture for indicated time periods, we did not observe transfer of tumor cells' mitochondria into recipient CECs, or increased mitochondrial mass/number in CECs, showing that EVs transport mtDNA, rather than the mitochondria themselves, into CECs (Supplementary Fig. 5a-e).

2. The authors show that conditioned medium of cancer cells on FHC cells

increases expression of mtDNA encoded genes, and report an increase in OXPHOS. However, they also show that EVs do not affect the levels of nuclear DNA-encoded mitochondrial proteins which are also critical for OXPHOS. It is unclear how this imbalance in proteins can lead to a functional and increased oxidative phosphorylation.

Response: We appreciate this insightful comment. Our results show that elevated levels of mtDNA-encoded proteins can promote OXPHOS. Previous studies also demonstrate that the changes in the content of mtDNA and the encoded proteins are the main cause of OXPHOS dysfunction in cancer [Xiao, Y. et al. *Cancer Res.* **80**, 3830-3840 (2020)]. Another study indicates that cellular respiration is effectively enhanced by the elevated translation levels of mtDNA-encoded ND1/ND6 independent of nuclear DNA-encoded mitochondrial proteins [Schöller, E. et al. *Mol. Cell* **81**, 4810-4825 (2021)]. This is most likely because the enzymatic reactions in OXPHOS are usually unsaturated. Consequently, following the increased expression of mtDNA-encoded proteins, the spare catalytic capacity of the existing enzymes encoded by nuclear DNA can quickly accommodate the increased metabolic flux upstream and downstream, thereby increasing the overall metabolic rate. In fact, this effect of accelerated metabolism induced by upregulated expression of individual proteins exists in various metabolic networks. Huppertz et al. report that regulation of enolase 1 activity affects glycolysis in cultured human cells [Huppertz, I. et al. *Mol. Cell* **82**, 2666-2680 (2022)]. Wu et al. demonstrate that activation of G6PD transcription promotes tumor cell proliferation by enhancing the pentose phosphate pathway[Wu, S. et al. *Cancer Res*. **78**, 4549-4562 (2018)]. We propose that this general and intrinsic mechanism allows cells to swiftly alter their metabolic profile in response to environmental stress. We have rewritten these discussions based on this phenomenon in lines 400-404 in the revised manuscript as follows: A reasonable explanation for this is that enzymatic reactions during OXPHOS, without stimulation, are usually unsaturated, thus providing the considerable reserve capacity for respiratory chain enzymes[30]. When several key components are elevated, the catalytic reserve capacity of other enzymes will adapt to the increased metabolic flux, raising

the total respiratory level.

3. The authors use FHC cells as a model for normal CEC, however these are tumorigenic cells with multiple genomic abnormalities (including mutations in TP53). Assays should be performed with primary murine organoids, derived from WT mice.

Response: Thanks for your kind suggestion, pointing out that the use of organoids can enhance the validity of our findings from studies via FHC cells. We constructed and cultured murine colonic epithelial organoids, the morphology and histology of which were shown in Fig. 5a. Epcam, a marker of epithelial cells, was also identified by whole mount immunofluorescence staining (Fig. 5b). Following co-culture with murine tumor cell-derived mtDNA-rich EVs, the organoids exhibited a remarkable increase in mtDNA copy number, and they also showed enhanced expression of mitochondrial-encoded proteins. As expected, the mitochondrial functions indicated by mitochondrial ROS and membrane potential were also significantly enhanced. Furthermore, the absence of mtDNA in EVs completely eliminated these effects, indicating that the transfer of mtDNA mediates the EV-induced changes in organoids (Fig. 5c and Supplementary Fig. 7a). In conjunction with the other in vitro studies, we reveal the intercellular delivery of mtDNA from colon cancer cells to normal colonic epithelial cells, leading to high-OXPHOS phenotype in recipient cells. We have presented these results in Fig. 5 and Supplementary Fig. 7, and described them in lines 234-239 in the revised manuscript as follows: Since organoids are frequently employed to imitate in vivo situations, we incubated murine colonic epithelial organoids (CEOs) with MC38-derived EVs. Organoid characterization was performed (Fig. 5a, b). Expectedly, CC cell-derived EVs dramatically increased the mtDNA content, mitochondrial proteins, ROS levels, and $\Delta\Psi$ m in CEOs, which was significantly attenuated by EV-mtDNA depletion (Fig. 5c and Supplementary Fig. 7a), supporting the conclusion that EV-mtDNA transfer enhances OXPHOS in CECs.

4. There are several questions/concerns around the in vivo studies. Specifically,

regarding the AOM/DSS model

• The authors show increased mtDNA content in normal adjacent tissue (NAT) of tumour bearing animals, compared to healthy mucosa of non-tumour bearing animals. However, there is no data showing that tumours in this model release EVs containing mtDNA.

Response: Thank you for this concern. To address this issue, we isolated and cultured tumor cells from the AOM/DSS model, purified their secreted EVs, and identified EV-packaged mtDNA via agarose gel electrophoresis of PCR products. As shown in Supplementary Fig. 3d, the presence of murine mtDNA fragments encoding Cox3, Cytb, and Nd2 indicates that tumors in this model release EVs containing mtDNA. The new data have been added in Supplementary Fig. 3 and written in lines 150-152 in the revised manuscript as follows: Moreover, mtDNA was also enriched in murine EVs derived from tumor cells, which were isolated from the murine orthotopic CC model (Supplementary Fig. 3d).

• The in vitro data suggest a proliferation advantage for CRC cells when cocultured with EV-exposed FHC cells. To validate this in vivo, the authors perform i.p. injection of EVs (3×10e9 EVs, once every 3 days for a total of 60 days) and show increased tumour burden (Figure 5E-G).

o AOM/DSS is a model of inflammation associated CRC. EVs have been shown to trigger an innate immune response. Can the authors show that their regime does not trigger an additional inflammatory response which can affect tumour development?

Response: Thanks for this constructive suggestion, as it is very helpful to improve the solidity of our work in this study. Therefore, we first statistically analyzed the colon length, an indicator of bowel inflammation severity, and observed no significant difference between groups with or without EV treatment (Supplementary Fig. 7e). Next, we performed HE staining and standardized pathological scoring of mouse colon tissues and revealed that the colonic inflammation score of animals that received EV treatment was not significantly altered relative to that of control

(Supplementary Fig. 7f). Moreover, ELISA of colonic tissue lysates also showed no significant changes in the levels of dominant pro-inflammatory cytokines in EV-injected mice (Supplementary Fig. 7g). The above results suggest that administration of EVs at the dose and duration we used promotes tumor progression independent of colonic inflammation in our animal model. We have added these findings in Supplementary Fig. 7 and rewritten them in lines 253-255 in the revised manuscript as follows: It is necessary to point out that the severity of colonic inflammation evaluated by colon length, pathological scores, and the levels of pro-inflammatory cytokines was not affected by EV administration (Supplementary Fig. 7e-g).

o Can the authors show that uptake of EVs and increased expression of mitochondrial proteins happens in CECs only, and not in tumour cells which would make it difficult to discriminate between 'CEC-Tumour communication' and direct effects on tumour epithelial cells?

Response: Thank you for the suggestion. We evaluated the mtDNA content in tumor cells in the in vivo model to address this issue. However, no obvious changes in mtDNA copy number were observed in tumors from EV-treated mice (Supplementary Fig. 7c). Moreover, neither mtDNA-rich nor mtDNA-deficient EVs altered the expression of mitochondrial-encoded proteins in tumor cells in vivo (Supplementary Fig. 7d). This may be because the high level of mtDNA in colon cancer cells compared with that in normal colonic epithelial cells, as shown in Fig. 2a and previous reports[Sun, X. et al. *Signal Transduct. Target. Ther.* **3**, 8 (2018)], had reached saturation and was sufficient for tumor survival. Therefore, tumor cells may protect themselves from the additional burden of oxidation. These results have been added in Supplementary Fig. 7 and written in lines 248-249 in the revised manuscript as follows: However, no change occurred in mtDNA copy number and the encoded protein levels extracted from tumor tissues (Supplementary Fig. 7c, d).

o How representative is the level of such exposure of EVs to a patient setting?

Similar question applies to the experiments injecting EVs from MC38 cells into WT mice (Figure 5A,B).

Response: Thank you for this concern. According to previous reports, the EV dose used in this study is commonly applied for chronic intraperitoneal injection in mice[Mendt, M. et al. *JCI Insight* **3**, e99263 (2018)]. In response to this question, we examined whether this dose of EVs was excessively high. We quantified the content of EVs in the NAT of mice that received EV injection. Our results showed no significant change in the content of EVs in the NAT, indicating that this dose was not unrealistic (Supplementary Fig. 7h). This observation may be because the amount of injected EVs is within the tolerance range of the cells, and they can be quickly taken up and processed by the colon epithelial cells. Therefore, the concentration of EVs in the tissue does not change significantly. Since there is a large difference in the EV concentration used for humans and mice, researchers need to re-evaluate it before conducting human clinical trials in the future. These results have been shown in Supplementary Fig. 7h in the revised manuscript.

o Which other tissues are affected by these EVs?

Response: Thanks for your concern. We observed no macroscopic changes in other organs (lung, liver, heart, kidney, and brain) in EV-treated mice. Pathological examination also revealed no suspected lesions in these major organs. The results have been supplemented in Supplementary Fig. 8 and described in lines 259-260 in the revised manuscript as follows: Moreover, the experimental dose of EVs had no obvious pathological effect on other major organs of mice (Supplementary Fig. 8).

• As per the model there should be a gradient of ROS in NAT with higher levels being observed in closer proximity to the tumour. This has not been shown in vivo or in patient samples.

Response: Thanks for this suggestion. As shown in Supplementary Fig. 3a, the ROS level in NAT (2 to 5 cm from tumor margin) was higher than that in paired distant colonic tissues (DT, > 5 cm from tumor margin) clinically, indicating a gradient of

increased ROS closer to the tumor. Moreover, in vivo studies also exhibited higher ROS in NAT than in healthy control (Supplementary Fig. 3b). The results have been added in Supplementary Fig. 3 and written in lines 136-139 and 146-149 in the revised manuscript as follows: Intriguingly, NAT also exhibited an increase in mtDNA content and ROS levels compared with paired DT in CC patients, strongly suggesting that tumor tissues may influence their surroundings (Fig. 2a and Supplementary Fig. 3a).

As expected, the mtDNA copy number and ROS levels were significantly increased in CECs isolated from NAT in tumor-bearing mice relative to those isolated from normal control mice (Fig. 2e and Supplementary Fig. 3b).

Minor comments

1. The study is based on the literature observation that oxphos is higher in tumour-adjacent colon compared to healthy mucosa. However, this is not a well-established observation, and is only supported by transcriptional profiling, rather than by functional studies (reference 11).

Response: Thank you for this kind reminder. As you noted, the previous literature only reported aberrant elevation of OXPHOS in NAT based on transcriptional profiling without experimental validation, due to limited knowledge of tumor-NAT crosstalk. As a complement, our initial investigation of clinical specimens revealed that NAT had higher mtDNA content than matched distal colon tissues (Fig. 2a). Also, mtDNA-encoded protein expression in NAT rose with EV-mtDNA level correlated with tumor stage (Fig. 2b, c). These observations are consistent with prior reports and motivate us further to elucidate the underlying mechanisms of this intriguing phenomenon. As a functional complement to the literature, we acknowledge that measuring the oxygen consumption rate of NAT-derived primary colonic epithelial cells by Seahorse assays may be the optimal choice. However, the isolation of primary cells involves many steps that have strong impacts on the cell metabolism, such as prolonged enzymatic digestion and repeated mechanical stimulation, which perturb the metabolic state of the primary cells, making it technically challenging to assess

their OXPHOS level by Seahorse assays accurately. Given that, in the revised manuscript, we supplemented tissue in situ ROS detection to demonstrate that the ROS level in NAT was elevated relative to that in matched distal colon tissues, implying significant functional alterations in oxidative respiration (Supplementary Fig. 3a). Moreover, the animal model also indicated increased ROS level in NAT (Supplementary Fig. 3b). Therefore, together with other in vitro and in vivo experiments, the foundation of this study is convinced.

2. What else makes up the EVs shedded by these models? Can this also affect metabolism of NAT?

Response: Indeed, EVs may harbor other metabolic regulators. For instance, EV-mediated TFAM mRNA transfer in recipient cells prevents inflammation and mitochondrial damage[Zhao, M. et al. *ACS Nano* **15**, 1519-1538 (2021)]. Trophoblast-derived EVs rewire glucose metabolism in NK cells to maintain pregnancy via HLA-E[Jiang, L. et al. *Int. J. Biol. Sci*. **17,** 4377-4395 (2021)]. We have previously reported that let-7a secreted via EVs regulates OXPHOS in colon cancer cells[Liu, Y. D. et al. *J. Exp. Clin. Cancer Res.* **40**, 31 (2021)]. However, the modulation of normal epithelial cells adjacent to colon cancer by EVs is scarcely reported. We initiated our study by observing aberrant elevation of mtDNA and OXPHOS in normal epithelial cells adjacent to colon cancer, and the significant association between EV-mtDNA levels and tumor stage. We verified that tumor-derived EVs are indispensable for enhancing OXPHOS in normal colonic epithelial cells, as the tumor cell conditioned medium devoid of EVs lost its stimulatory effect on recipient cells. Moreover, the employment of IMT1B and mtDNA-deficient EVs demonstrated that EV-mediated mtDNA intercellular transfer is the impetus and main contributor to this phenomenon. The impacts of various constituents in EVs on epithelial cells warrant further investigation, but at least, mtDNA is among the most crucial ones, and hence also the focal point of this study. We have incorporated the discussion in lines 409-415 in the revised manuscript as follows: Besides mtDNA, several other OXPHOS-related molecules have been

identified to be transferred by EVs. For instance, EV-mediated TFAM mRNA transfer in recipient cells prevented inflammation and mitochondrial damage[32]. Trophoblast-derived EVs rewired glucose metabolism in NK cells to maintain pregnancy via HLA-E[33]. We have previously reported that let-7a secreted via EVs regulates OXPHOS in CC cells[34]. This study reveals the metabolic crosstalk between CC cells and CECs occurring via EV-mtDNA transfer, adding to our knowledge of metabolic reprogramming within the intestinal TME.

3. In Figure 6A, it's unclear why a 3 cell-based model was used. RKO cells were cocultured with FHC pretreated with EV from SW480 cells. As per the proposed mechanism, simply co-culturing CRC cells with CEC cells should produce the same results (CRC releases EVs, CECs produce TGFB1). At very least, pretreatment should be done with EVs from the same cell line.

Response: We are grateful for this concern. Our study showed that two colon cancer cell lines, SW480 and HCT116, increased OXPHOS of CECs by transferring mtDNA via EVs. We then discovered that EV-educated CECs could boost the malignancy of SW480 and HCT116 and amplify the malignant phenotype of two other randomly selected colon cancer cell lines, RKO and HT29, implying that such intercellular communications are universal. To emphasize this universality, we used RKO and HT29 for subsequent experiments to address the concern that this interaction mechanism is limited to specific tumor cell lines (SW480 and HCT116) and CECs. This experimental strategy, that is, using two tumor cell lines to activate recipient cells and then co-culturing recipient cells with another two tumor cell lines to demonstrate the universality of the mechanism, has been employed in previous literature[Zhao, S. et al. *J. Extracell. Vesicles* **11**, e12186 (2022)]. The experimental results of treated FHC promoting the malignancy of SW480 and HCT116 have been added in Supplementary Fig. 10 and clarified in lines 262-266 in the revised manuscript as follows: The phenotypic assays suggested that FHC cells educated by mtDNA-sufficient EVs had the most pronounced promotive effects on the proliferation, migration and invasion abilities of CC cells, which were weakened by

EV-mtDNA depletion (Supplementary Fig. 9a-h). Same results were observed in SW480 and HCT116 cells, indicating a widespread phenomenon (Supplementary Fig. 10a-h).

4. Data on shTFAM (Figure S3A) needs to be presented differently to also allow interpretation of shTFAM on cells that were not exposed to EVs (i.e. normalize all data shNT/PBS).

Response: Thanks for your suggestion. We have displayed the processed data in Supplementary Fig. 4b, which illustrate the impact of shTFAM on mtDNA content. These findings reveal that despite the fact that TFAM knockdown diminished the endogenous mtDNA content, it did not abrogate the fold enhancement of mtDNA induced by EVs, implying that EVs can substantially restore the mtDNA copy number of recipient cells via an exogenous pathway.

5. Can the authors clarify the results obtained with IMT1B (Fig 3A) and shTFAM (Fig S3A)? Both are strategies to impair mitochondrial replication, yet the results obtained seem opposite.

Response: We apologize for the confusion that may have been caused by the initial presentation of data on shTFAM. In the revised manuscript, we have shown that the mtDNA alteration profile was similar in groups treated with IMT1B or shTFAM (Fig. 3a and Supplementary Fig. 4b).

6. All Seahorse data needs to be normalized.

Response: We should have clarified that the Seahorse data presented in the manuscript had been normalized. In the revised version, we have added the following statement in lines 595-597: Each well in the assay plates was verified to be sub-confluent and to have a comparable cell density on the day of assay, and the results were normalized by cell number.

Reviewer #3; expert in mitochondria:

The manuscript by Guan et al., is a detailed study of extracellular vesicles (EVs) in terms of mitochondrial DNA (mtDNA) content in EVs from colon cancer cells and its effects in recipient cells, which are in this particular case, cells nearby the tumor. Therefore authors have explored the paracrine action of EVs and found that these EVs, enriched in mtDNA, are able of transferring functional genome that will impact in the content on mitochondrial respiration components in recipient cells, changing their metabolic function. The intra-peritoneal injection of the EVs has a clear impact in colon tumors in mice that are induced by chemical agents.

This is a well-executed work, which includes a number of different techniques to analyze the content and role of mtDNA in cancer development. A major issue here is that authors only envisage a component of EVs having a specific role in tumorigenesis, which has in turn been associated to the ability of other cells to act as a regulator of homeostasis, such as myeloid cells. Is mtDNA the only relevant component having a role here? In the model used by the authors, it seems that these EVs have only effect on the tumor; however, the effect of mtDNA as a DAMP (danger associated molecules) by immune cells (mainly myeloid) in the peritoneum of injected mice has not been addressed. It is required that authors demonstrate that the EVs only impact in the tumor-surrounding cells: do EVs bear a specific receptor or molecule that allow their binding to these cells? Are other cells decorated or modified by the EVs in these mice?

Response: Thank you very much for your insightful comment. As you pointed out, EVs contain various bioactive molecules with complex functions. For instance, EV-mediated TFAM mRNA transfer in recipient cells prevents inflammation and mitochondrial damage[Zhao, M. et al. *ACS Nano* **15**, 1519-1538 (2021)]. Trophoblast-derived EVs rewire glucose metabolism in NK cells to maintain pregnancy via HLA-E[Jiang, L. et al. *Int. J. Biol. Sci.* **17**, 4377-4395 (2021)]. However, we acknowledge that assessing the impact of each molecule in EVs on

tumor progression is challenging in a single study. So, we focus on the major effectors, especially those less studied. Our in vitro and in vivo experiments showed that EVs' effect was greatly attenuated after removing their mtDNA, which at least indicates that mtDNA in EVs is one of the indispensable mediators for their function. Therefore, EV-mtDNA naturally became the main point of this study. The in vitro experiments demonstrated that without other types of cells, the communication between tumor cells and normal colonic epithelial cells mediated by EV-mtDNA was sufficient to induce a significant pro-cancer phenotype. The in vivo experiments also supported this conclusion.

Your valuable suggestion reminds us that immune cells, represented by myeloid cells, may also play a potential role. Given this, we standardized the assessment of colonic inflammation by measuring the colon length and inflammation score. We found no significant effect of EV-mtDNA on them (Supplementary Fig. 7e, f). Then, we further examined the infiltration of macrophages and neutrophils in the tumor by tissue immunofluorescence, and the results suggested that EV-mtDNA did not significantly affect the infiltration number of myeloid cells (Fig. 5j, k). Furthermore, we measured the levels of major pro-inflammatory cytokines secreted by immune cells in the colon tissue lysates. The results also suggested that EV-mtDNA did not significantly interfere with the immune inflammatory response (Supplementary Fig. 7g). Therefore, under the dose and time conditions we used to treat mice with EVs, the effect on myeloid cells and intestinal inflammatory immunity was minimal. Additionally, we found that EVs did not cause noticeable pathological changes in other major organs of mice (Supplementary Fig. 8).

We fully agree with your view that EVs also have regulatory effects on different types of cells because it is a widely accepted fact that EVs can be taken up and utilized by various cells. However, our results revealed that mtDNA transfer between tumor cells and adjacent normal epithelial cells is one of the essential pro-cancer mechanisms that does not require immune system mediation. The role of EVs in communication between tumor cells and other types of cells, such as myeloid cells, is another exciting and valuable topic worth more dedicated research. We appreciate this suggestion that

has important guidance for our future investigations. The supplementary results have been added in Fig. 5, Supplementary Fig. 7, and Supplementary Fig. 8 and written in lines 253-260 as follows: It is necessary to point out that the severity of colonic inflammation evaluated by colon length, pathological scores, and the levels of pro-inflammatory cytokines was not affected by EV administration (Supplementary Fig. 7e-g). Meanwhile, macrophage and neutrophil infiltration in the tumors, as well as the content of colonic EVs, were unchanged upon EV injection (Fig. 5j, k and Supplementary Fig. 7h). These results suggest that colonic inflammation is not involved in the tumor-promoting effects of EVs used in moderation. Moreover, the experimental dose of EVs had no obvious pathological effect on other major organs of mice (Supplementary Fig. 8).

Several other points should be addressed by authors:

1.- An important question that authors address only partially in the manuscript is whether the role described for mtDNA is a general mechanism or a specific effect observed in the particular case of EVs produced by CC cells. A relevant control would be the comparative analysis of the capacity of circulating EVs from HC, which also includes mtDNA among their components.

Response: Thanks for this suggestion. We co-cultured FHC cells with circulating EVs from HC or CC patients to address this issue. A pronounced increase in mtDNA content was observed in FHC cells educated with EVs from CC patients. Although a similar increasing trend was present in FHC cells educated with EVs from HC, the difference was insignificant. This phenomenon may be caused by the different abundance of mtDNA in circulating EVs, as identified in Fig. 1b. The results were shown in Supplementary Fig. 4a and rewritten in lines 188-190 in the revised manuscript as follows: Notably, education with serum EVs derived from CC patients, instead of healthy individuals, significantly increased the mtDNA content in FHC cells, suggesting the potential mtDNA transfer by mtDNA-sufficient EVs (Supplementary Fig. 4a).

2.- In general, it is difficult to detect differences in the histopathologic samples showed in the Ms (Fig 1b, 1d, 5c, 6g, 6h, 6j, 8d). Same applies for immunofluorescences (Fig. 2l, 4a, 5a, 7e, 7g). In general, the images showed are too small to observe any kind of difference: these are not evaluable (even if quantification is provided). Merged images without individual channels are not very informative.

Response: Thanks for this comment. We have improved the resolution of the images in the revised manuscript. All major fluorescence results are presented as individual channels and merged images simultaneously. All figures are provided as high-resolution separate files, providing greater clarity for the Reviewer.

3.- In Figure 6a, the authors perform a sequencing of samples treated with SM+ EVs vs PBS and observe an upregulation of the TGFb1 pathway. However, PBS here does not seem to be the best control, as one cannot rule of the effect that the EV itself or any other of its components is having when comparing to PBS. In this sense, the authors should have compared SM+ EVs with EVs that do not bear mitDNA. A validation including this sample, either by transcriptomics or by parallel validation by qPCR of the three conditions of TGFB1-related genes identified in that dataset should be performed.

Response: Thanks for this suggestion. The qPCR analysis of several TGFβ1-related genes has been performed to address this issue. The results were shown in Supplementary Fig. 11a and clarified in lines 274-275 in the revised manuscript as follows: This was further confirmed by qPCR analysis of several TGFβ-related genes upon indicated treatment (Supplementary Fig. 11a).

4.- Can authors test the effect of TGB1 plus EVs? Is this treatment additive and/or synergic?

Response: We observed that the combination of TGFβ1 and CM from EV-pretreated FHC cells promoted tumor cell proliferation, migration and invasion more

significantly than either alone, indicating their synergic effect on tumor progression. The results were shown in Supplementary Fig. 13 and clarified in lines 304-306 in the revised manuscript as follows: Of note, exogenous TGFβ1 and CM from FHC cells pretreated with mtDNA-rich EVs synergistically promoted tumor progression (Supplementary Fig. 13a-d).

Minor points :

1.Which is the level of TFAM protein in the recipient cells of mtDNA?

Response: As shown in Supplementary Fig. 4b, the level of TFAM protein in the recipient cells of mtDNA was not altered obviously.

2. In Fig 1d: there seems to be some bands missing (as compared to Fig. 1h). In Figure 1d, why are there some fragments that are not amplified?

Response: As you mentioned, some patients have deletions in their amplified mtDNA, while others have intact mtDNA (Fig. 1d and Supplementary Fig. 1c). We hypothesize that this phenomenon is attributable to mutations occurring in the mtDNA of different individuals, including deletion and point mutation, which lead to the partial absence of amplified fragments.

3. Is there any kind of typos in Fig 2h?

Response: We have corrected it, thanks for your reminder.

4. Statistical analysis should be provided for the differences in basal and spare respiratory capacities in figure 2i.

Response: Statistical analysis of basal and spare respiratory capacities corresponding to Fig. 2i has been shown in Supplementary Fig. 3g, h.

5. In line 38 a reference should be provided.

Response: A reference has been provided for the sentence: Increasing evidence

suggests that nontumor cells educated by cancer cell-derived extracellular vesicles (EVs) play an essential role in tumor development[5].

6. Lines 260-262 seem to need some editing.

Response: We have re-written it in lines 289-294 in the revised manuscript as follows: In accordance with the previous studies that showed the TGFβ/SMAD signaling pathway as a key regulator of epithelial-mesenchymal transition (EMT)[21], we found that the CM from FHC cells educated by EV-mtDNA induced a TGFβ1-dependent EMT phenotype in tumor cells, characterized by increased levels of phosphorylated SMAD2/3, Vimentin, and Snai1 and decreased level of E-cadherin (Fig. 6f and Supplementary Fig. 11h).

7. Data not shown should not be included (line 427).

Response: We have deleted this sentence in the revised manuscript.

REVIEWER COMMENTS

Reviewer #1 (Remarks to the Author):

The authors have addressed some of the issues. However, there are still some concerns about the methodology and the rigor of the analyses. Statements such as "Considering that exosomes consist of traditional subpopulations and non-membranous components termed "exomeres" are erroneous. Exomeres are not a class of exosomes. In fact they are not even extracellular vesicles because they do not have a membrane. New data comparing exosomes to exomeres using gradient centrifugation to purify the EVs are interesting, but it is unclear why they decided to compare with exosomes with exomeres, when the question actually was: have you considered much larger EVs as the ones that carry mitochondria or mitochondria's stuff? I would assume exomeres are too small to package mitochondria. I am also still concerned about the method to make ρ0 cell lines. It is well known that the method offers significant challenges and comes with limitations, but this is not acknowledged. However, it is difficult to imagine that not only the ρ0 cells are alive, but that they also produce EVs without mitochondria without additional data.

Reviewer #2 (Remarks to the Author):

For the revision of this work, the authors have substantiated their findings with additional experiments and by including references to existing literature. The following questions remain:

1. The mechanism of transfer of intact mitochondrial DNA into recipient host mitochondria via EVs remains a non-trivial observation that requires further research and this has not been better understood during the revisions of this paper. This needs to be acknowledged explicitly in the discussion.

2. The authors state that enzymatic reactions relevant to oxphos are typically unsaturated and that increased expression of the mitochondrial components is therefore sufficient to increase cellular respiration. One could argue that this is mostly the case if the mtDNA components are rate limiting. Proteins that are often rate limiting and part of the electron transport chain (e.g. cytochrome c oxidase) consist of multiple mitochondrially encoded and nuclear encoded subunits. In this case, the authors only show data for one mitochondrial subunit (cox1). What happened to the other subunits? Can this be discussed?

3. The authors have chosen to not analyse the effects of EVs on the metabolism of other tissues and only mention no obvious histopathological effects on major organs. However, as far as the data indicate there are also no major histopathological effects of these EVs on the intestine, yet significant metabolic rewiring is described. This open question needs to be mentioned in the manuscript.

Reviewer #3 (Remarks to the Author):

Authors have properly addressed all my queries raised in the previous review.

RESPONSE TO REVIEWERS' COMMENTS

Reviewer: 1

The authors have addressed some of the issues. However, there are still some concerns about the methodology and the rigor of the analyses.

1. Statements such as "Considering that exosomes consist of traditional subpopulations and non-membranous components termed "exomeres" are erroneous. Exomeres are not a class of exosomes. In fact they are not even extracellular vesicles because they do not have a membrane. New data comparing exosomes to exomeres using gradient centrifugation to purify the EVs are interesting, but it is unclear why they decided to compare with exosomes with exomeres, when the question actually was: have you considered much larger EVs as the ones that carry mitochondria or mitochondria's stuff? I would assume exomeres are too small to package mitochondria.

Response: We highly appreciate your insightful suggestions. Exomeres lack membrane structures and have minimal diameters, limiting their influence on our study. We removed the content related to exomere identification to avoid confusing or distracting the readers. We also recognize your important observation regarding the possibility of large EVs (lEVs) carrying mitochondria or mitochondrial components. Therefore, we further compared the mtDNA content in lEVs and small EVs (sEVs) derived from colon cancer cells. Both gel electrophoresis qualitative analysis and qPCR quantitative detection revealed that mtDNA was predominantly enriched in sEVs rather than lEVs (Supplementary Fig. 2m, n). We also assessed the levels of several representative mitochondrial proteins in lEVs and sEVs. We discovered that the levels of mitochondrial proteins in both EV subtypes were very low or even undetectable, although their respective markers were explicitly enriched (Supplementary Fig. 2o). Based on this, we infer that the distribution of mitochondrial components in EVs may vary depending on the tissue or cell type. As evidence for this inference, a mass spectrometry study found that mitochondrial proteins (such as ND4, SDHA, SDHB, UQCRC2, and COX1) were absent in sEVs and lEVs derived

from human colon cancer cells SW620 but were highly enriched in EVs secreted by human glioma cells U87 [Rai A, et al. *J Extracell Vesicles*. 2021, 10(13):e12164].

Notably, our co-culture experiments directly exclude the possibility of colon cancer cells transferring mitochondria to normal colonic epithelial cells (Supplementary Fig. 5). Furthermore, complete conditional medium (CM) from tumor cells markedly increased the levels of oxidative phosphorylation in FHC cells. After removing the pre-characterized sEVs with mtDNA enrichment, the CM lost its ability to trigger the phenotype in FHC cells. These findings show that sEVs, not lEVs or the other components, play a dominant role (Fig. 2i-k and Supplementary Fig. 3h-k). Meanwhile, following the inhibition of the sEV production, the total amount of mtDNA secreted by tumor cells declined sharply (Supplementary Fig. 2f). Combining these results with the other findings in this manuscript, we show that the transport of mtDNA by sEVs derived from colon cancer cells, rather than the transfer of mitochondria, is one of the most important mechanisms for modulating the oxidative phosphorylation of normal colonic epithelial cells. The mechanism of selective secretion of mitochondrial components by vesicles is complex and fascinating and also an important scientific question that warrants more future research. We have added the new results in Supplementary Fig. 2 and have written them in lines 120-125 in the revised manuscript as follows: Since lEVs may also carry mitochondrial components, we further compared the levels of mitochondrial constituents in lEVs and sEVs secreted by CC cells[18]. We verified that mtDNA was predominantly enriched in sEVs rather than lEVs and that both vesicle types contained negligible amounts of mitochondrial proteins, indicating that the selective enrichment of mtDNA in sEVs was not a random occurrence (Supplementary Fig. 2m-o). Moreover, we have addressed the following point in the discussion section in lines 440-442 in the revised manuscript: MtDNA is enriched in CC cell-derived EVs, but the mechanism by which mtDNA is selectively released into EVs remains obscure.

2. I am also still concerned about the method to make ρ0 cell lines. It is well known that the method offers significant challenges and comes with limitations,

but this is not acknowledged. However, it is difficult to imagine that not only the ρ0 cells are alive, but that they also produce EVs without mitochondria without additional data.

Response: We are very grateful for your valuable comment. We acknowledge that, as you have pointed out, this model has some limitations. We have incorporated the following discussion in lines 445-447 in the revised manuscript: Existing methods are still challenging to remove mtDNA from cells fully. Hence, more effective ρ0 cell construction technologies are needed for future mtDNA studies. However, we note that EtBr induction, employed in this study, is a classic technique for establishing mtDNA-deficient ρ0 cell lines, with a relatively mature operational procedure and broad acceptance. We have observed that numerous high-quality studies have employed this method to produce ρ0 cells for research purposes, such as Tan AS et al. (*Cell Metab*. 2015, 21:81-94) and Nakahira K et al. (*Nat Immunol*. 2011, 12:222-230). It can be seen that this technique is widely utilized under the current technical circumstances.

It is challenging to eliminate mtDNA completely, so detecting mtDNA content in ρ0 cells is crucial for evaluating their suitability. We achieved a significant reduction of the mtDNA copy number in the ρ0 cells, reaching only about 1% of the wild-type cells (Fig. 4c). This is superior to the acceptance level of many published papers (about 5%, Nakahira K, et al. *Nat Immunol*. 2011, 12:222-230). Furthermore, the mtDNA content in ρ0-derived EVs is merely about 0.01% of that in wild-type EVs (Fig. 4c). Sanger sequencing also confirmed that such a trace amount of mtDNA transfer was negligible (Supplementary Fig. 7b, c). Therefore, it can serve as a mtDNA depletion control for wild-type EVs.

We also investigated the survival status of ρ0 cells. The experimental results revealed that ρ0 cells had similar proliferation potential and cell viability to parental cells (Supplementary Fig. 6b, c). Moreover, we detected no significant difference in the apoptosis level of ρ0 cells and parental cells by using TUNEL, Caspase3 activity, and apoptotic protein content assays (Supplementary Fig. 6d-g). The parental and ρ0 cells also exhibited comparable sensitivity to apoptosis inducers (Supplementary Fig.

6d-g). Consistent with our results, previous reports indicated that colon cancer ρ0 cells maintained similar proliferation potential and cell viability as parental cells [Mou JJ, et al. *Biomed Pharmacother*. 2018, 103:729-737]. We further characterized the EVs derived from ρ0 cells and confirmed that these EVs displayed typical features under electron microscopy, size distribution, and EV markers (Supplementary Fig. 6h-j). In line with this, ρ0 cells can secrete typical EVs as shown in previous literature [Sansone P, et al. *Proc Natl Acad Sci U S A*. 2017, 114:E9066-E9075]. Regarding your concern, the mechanism of how ρ0 cells can survive is a topic of interest for many researchers. Some studies have proposed that mtDNA-deficient cells can adapt to metabolic stress and secure the bioenergetics required for survival [West AP, et al. *Nature*. 2015, 520:553-557]. ROS-induced mtDNA fragmentation is a crucial trigger for cell apoptosis, and mtDNA deficiency protect ρ0 cells to some degree [Tann AW, et al. *J Biol Chem*. 2011, 286:31975-31983]. Another study verified that ρ0 cells incapable of aerobic ATP synthesis could survive by reorganizing the mitochondrial proteome [Guerrero-Castillo S, et al. *EMBO J*. 2021, 40:e108648]. These new findings have been added in Supplementary Fig. 6 and written in lines 211-215 in the revised manuscript: Stable mtDNA deletion, resulting in a typical vacuolar structure in mitochondria, did not affect the cell proliferation, viability, and apoptosis, indicating that ρ0 cells retained their survival capacities (Fig. 4d and Supplementary Fig. 6a-g). Numerous ρ0 cell-derived EVs have also been identified (Supplementary Fig. 6h-j).

It should be mentioned that ρ0 cell types cannot be established by all cells, possibly due to their high reliance on mitochondrial oxidative phosphorylation and their vulnerability to mtDNA content variations [King MP, et al. *Methods Enzymol*. 1996, 264:304-313]. We have included the following discussion in lines 447-450 in the revised manuscript: Moreover, some kinds of cells that are critically dependent on mitochondrial energy supply or highly susceptible to mtDNA content changes are unlikely to survive as ρ0 cells[39], necessitating the development of novel research methods for these cell types.

Thanks again for your insightful comment, which is very helpful for improving

the rigor and depth of our research.

Reviewer: 2

For the revision of this work, the authors have substantiated their findings with additional experiments and by including references to existing literature. The following questions remain:

1. The mechanism of transfer of intact mitochondrial DNA into recipient host mitochondria via EVs remains a non-trivial observation that requires further research and this has not been better understood during the revisions of this paper. This needs to be acknowledged explicitly in the discussion.

Response: Thanks for your nice suggestion. We realize that this observation is important and requires further studies to elucidate the detailed molecular mechanisms. We have incorporated the discussion in lines 442-445 in the revised manuscript: We have demonstrated that EVs can deliver mtDNA to recipient mitochondria, which might involve membrane fusion between EVs and mitochondria, allowing EV cargo to enter the organelles. However, the detailed mechanisms of EV-organelle fusion, especially with mitochondria, remain elusive and warrant further investigation.

2. The authors state that enzymatic reactions relevant to oxphos are typically unsaturated and that increased expression of the mitochondrial components is therefore sufficient to increase cellular respiration. One could argue that this is mostly the case if the mtDNA components are rate limiting. Proteins that are often rate limiting and part of the electron transport chain (e.g. cytochrome c oxidase) consist of multiple mitochondrially encoded and nuclear encoded subunits. In this case, the authors only show data for one mitochondrial subunit (cox1). What happened to the other subunits? Can this be discussed?

Response: Thank you for raising this important and constructive issue. To address it, we performed qPCR assays and discovered that tumor cells preferentially enhanced the expression of mtDNA-encoded genes in recipient cells in an EV-dependent

manner (Supplementary Fig. 3g). Take cytochrome c oxidase (complex IV), for example, EVs augmented the expression of mtDNA-encoded COX1 and COX2, while the other two nuclear-encoded components (COX4I1 and COX6A1) stayed unchanged (Supplementary Fig. 3g). The rate-limiting catalytic center of complex IV consists of three core subunits, all of which are encoded by mtDNA (COX1, COX2, and COX3), whereas the nuclear-encoded subunits primarily facilitate the complex stability [Timón-Gómez A, et al. *Semin Cell Dev Biol*. 2018, 76:163-178]. This explains why the increased mtDNA-encoded subunits can regulate enzymatic reactions relevant to OXPHOS.

A further insight is that the assembly of mitochondrial complexes is a multistage process: mtDNA-encoded and nuclear-encoded subunits first combine into various intermediates with different ratios, and then these intermediates either join into complexes or directly contribute to the formation of supercomplexes, which modulate the activity of individual complexes [Vercellino I, et al. *Nat Rev Mol Cell Biol*. 2022, 23:141-161]. Consequently, when the ratio of mtDNA-encoded and nuclear-encoded subunits varies, the assembly efficiency of intermediates may improve, thus influencing the formation rate and function of complexes.

Another potential synergetic mechanism is that the activity of nuclear-encoded subunits could fluctuate via post-translational modifications (PTMs) even though their quantity remained constant. For example, acetylating the nuclear-encoded complex II subunit SDHA alters its activity [Li ST, et al. *Nat Metab*. 2020, 2:256-269]. EV-rewired mitochondrial metabolism may alter the concentrations of various metabolic intermediates, which could modulate protein activity via PTMs, such as acetylation and lactylation, affecting other modifications, such as SUMOylation [Liu W, et al. *Nature*. 2023, 616:790-797]. Consequently, the nuclear-encoded subunits with PTMs may cooperate with mtDNA-encoded counterparts.

Additional studies are required to corroborate these claims, suggesting many valuable avenues for our future research. We are grateful for the precious time and effort that you have spent on our manuscript. We have supplemented the new findings in Supplementary Fig. 3g and discussed it in lines 390-407 in the revised manuscript:

One reasonable explanation for this is that enzymatic reactions during OXPHOS, without stimulation, are usually unsaturated, thus providing a considerable reserve capacity for respiratory chain enzymes[27]. When several core subunits, frequently encoded by mtDNA[28], are elevated, the catalytic reserve capacity of other enzymes will adapt to the increased metabolic flux, raising the total respiratory level. One additional explanation is that the assembly of mitochondrial complexes is a multistage process: mtDNA-encoded and nuclear-encoded subunits initially merge into various intermediates with distinct proportions, and then these intermediates either assemble into complexes or participate in the formation of supercomplexes, which regulate the activity of individual complexes[28]. When the proportion of mtDNA-encoded and nuclear-encoded subunits changes, the assembly efficacy of intermediates may enhance, thereby affecting the formation and function of complexes. Another potential synergetic mechanism is that the activity of nuclear-encoded subunits could vary via post-translational modifications (PTMs). For instance, acetylating the nuclear-encoded complex II subunit SDHA modifies its activity[29]. EV-altered mitochondrial metabolism may change the levels of various metabolic intermediates, which could modulate protein activity via PTMs, such as acetylation and lactylation, influencing other modifications, such as SUMOylation[30]. Thus, the nuclear-encoded subunits with PTMs may collaborate with mtDNA-encoded counterparts to boost OXPHOS. These claims require further studies.

3. The authors have chosen to not analyse the effects of EVs on the metabolism of other tissues and only mention no obvious histopathological effects on major organs. However, as far as the data indicate there are also no major histopathological effects of these EVs on the intestine, yet significant metabolic rewiring is described. This open question needs to be mentioned in the manuscript.

Response: Thank you for this comment. We have added the following sentences in lines 259-263 in the revised manuscript: The metabolic effects of EVs on extra-intestinal organs are unclear, but we speculate that they have minimal impact on

OXPHOS in organs with intrinsic robust mitochondrial metabolism, such as the liver and brain. Moreover, the pathological environment surrounding the tumor may contribute to the regulatory function of EVs. However, these speculations warrant further research.

Reviewer: 3

Authors have properly addressed all my queries raised in the previous review.

Response: Thank you for your positive feedback and approval of our manuscript revisions.

REVIEWERS' COMMENTS

Reviewer #1 (Remarks to the Author):

The authors have addressed all remaining concerns.

Reviewer #2 (Remarks to the Author):

The authors have now addressed all of my comments.

RESPONSE TO REVIEWERS' COMMENTS

Reviewer #1: The authors have addressed all remaining concerns.

Response: We are glad we have addressed the reviewer's concerns.

Reviewer #2: The authors have now addressed all of my comments.

Response: We appreciate the positive comments from reviewer #2.
